# Supplementary material for: The Evolution and Characterization of the RNA Interference Pathways in Lophotrochozoa
Source: Genome Biol Evol. 2024 May 7;16(5):evae098. doi: 10.1093/gbe/evae098 (PMC11114477; doi:10.1093/gbe/evae098)
Supplement: evae098_Supplementary_Data [file evae098_supplementary_data.zip › Supplementary_materials.zip!/selected_miRNAs/NoGe_selected_miRNAs/mirdeep_pdf_files/NMRB01002521.1_Notospermus_geniculatus_strain_Ushimado_scaffold2521,_whole_genome_shotgun_sequence_38687.pdf]

Provisional ID	: NMRB01002521.1_Notospermus_geniculatus_strain_Ushimado_scaffold2521,_whole_genome_shotgun_sequence_38687
Score total	: 7330434.3
Score for star read(s)	: 3.9
Score for read counts	: 7330425.8
Score for mfe	: 1.5
Score for randfold	: 0
Score for cons. seed	: 3
Total read count	: 14378326
Mature read count	: 14378019
Loop read count	: 8
Star read count	: 299

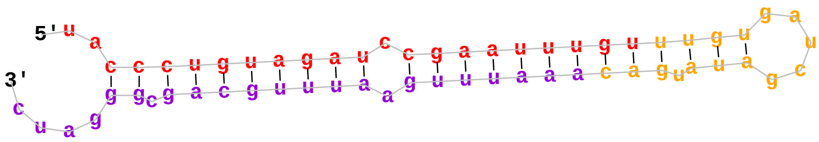

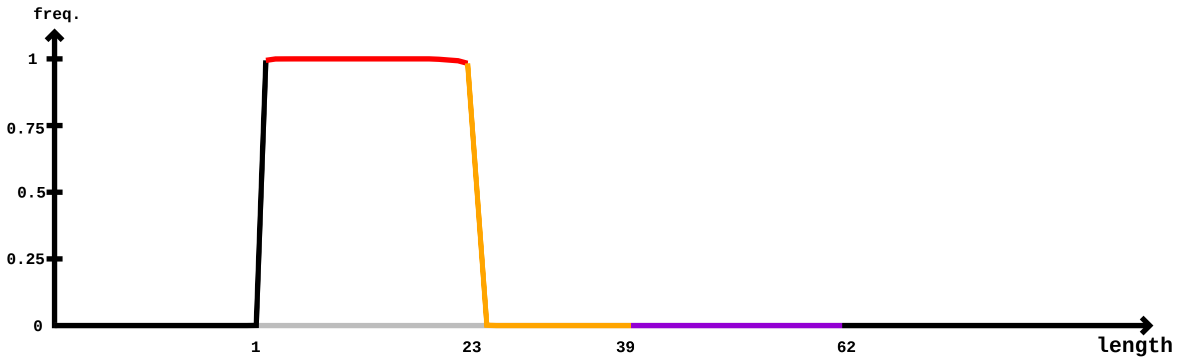

Mature Star

5'- agaguucgcccaucagcagauacccuguagauccgaauuuguuugugaucgauaugacaaauuugaauuugcagcgggaucugaaguuggguauaaucaacugacaggagga	-3'	obs		
agaguucgcccaucagcagauacccuguagauccgaauuuguuugugaucgauaugacaaauuugaauuugcagcgggaucugaaguuggguauaaucaacugacaggagga		exp		
.((....(((((.(..((((..(((((((((.((((((((((((.....))).))))))))).))))))).))..))))..).)))))....))..................	reads		mm	sample
...................auacccuguagaAccgaa...	4		1	Fm2
...................auacccuguagaAccgaau..	7		1	Fm2
...................auacccuguagauccgaCuuugu..	1		1	Fm2
...................Cuacccuguagauccgaauuugu..	217		1	Fm2
...................auacccuguagauccgaUuuugu..	1		1	Fm2
...................auacccuguagauccgaauuugu..	22		0	Fm2
...................auacccuguagaAccgaauuugu..	1		1	Fm2
...................Uuacccuguagauccgaauuugu..	1		1	Fm2
...................Cuacccuguagauccgaauuuguu...	9		1	Fm2
....................uaUccuguagauccgaau..	1		1	Fm2
....................uacccuguagGuccgaau..	2		1	Fm2
....................uacccuguagCuccgaau..	1		1	Fm2
....................uaccAuguagauccgaau..	1		1	Fm2
....................uacccugAagauccgaau..	1		1	Fm2
....................uacccuguagauccgaaC..	4		1	Fm2
....................uacccuguagauccgaau..	805		0	Fm2
....................uacccuguagauUcgaau..	1		1	Fm2
....................uacccuguagaucUgaau..	1		1	Fm2
....................Nacccuguagauccgaau..	1		1	Fm2
....................uacccuguagauccgaaA..	3		1	Fm2
....................uacccuguagauccgaaG..	5		1	Fm2
....................uacccuguagaAccgaau..	1		1	Fm2
....................uacccuguagUuccgaau..	1		1	Fm2
....................uacccuguagauccgaUu..	2		1	Fm2
....................uacUcuguagauccgaau..	1		1	Fm2
....................uacccuguagaucUgaauu...	4		1	Fm2
....................uacccuguagauccAaauu...	1		1	Fm2
....................uacccuguagaucAgaauu...	1		1	Fm2
....................uaGccuguagauccgaauu...	3		1	Fm2
....................uacccuguagauccgaUuu...	8		1	Fm2
....................uacccGguagauccgaauu...	1		1	Fm2
....................uacccuguagUuccgaauu...	3		1	Fm2
....................uacAcuguagauccgaauu...	1		1	Fm2

```
                    Mature                              Star
agaguucgcccaucagcagauacccuguagauccgaauuuguuugugaucgauaugacaaauuugaauuugcagcgggaucugaaguuggguauaaucaacugacaggagga
....................uacccuguagGuccgaauu.........................................................................    5      1    Fm2
....................uacccuguagauccgaauu.........................................................................    2952   0    Fm2
....................uacccuguagauccgaauA.........................................................................    118    1    Fm2
....................uCcccuguagauccgaauu.........................................................................    1      1    Fm2
....................Nacccuguagauccgaauu.........................................................................    2      1    Fm2
....................uacccuguUgauccgaauu.........................................................................    1      1    Fm2
....................uacccuguagauccgaaGu.........................................................................    19     1    Fm2
....................uacccuguagauccgaauG.........................................................................    1      1    Fm2
....................uacccuguagauccgaCuu.........................................................................    6      1    Fm2
....................uacccuguagCuccgaauu.........................................................................    3      1    Fm2
....................uacccuguagauccgaaCu.........................................................................    26     1    Fm2
....................uaccAuguagauccgaauu.........................................................................    1      1    Fm2
....................uacccuguagauccCaauu.........................................................................    1      1    Fm2
....................uacccNguagauccgaauu.........................................................................    1      1    Fm2
....................Cacccuguagauccgaauu.........................................................................    1      1    Fm2
....................uacccuguagauccgaaAu.........................................................................    14     1    Fm2
....................uacccuguagauccgaGuu.........................................................................    4      1    Fm2
....................uacccuguagauUcgaauu.........................................................................    2      1    Fm2
....................uacccuAuagauccgaauu.........................................................................    1      1    Fm2
....................uacccuUuagauccgaauu.........................................................................    1      1    Fm2
....................uacccuguagGuccgaauuu........................................................................    2      1    Fm2
....................uacccuguagauccgaauuu........................................................................    1469   0    Fm2
....................Nacccuguagauccgaauuu........................................................................    1      1    Fm2
....................uacccuguagauccgaGuuu........................................................................    1      1    Fm2
....................uacccuguagCuccgaauuu........................................................................    2      1    Fm2
....................uacccuguagauccgaCuuu........................................................................    3      1    Fm2
....................uacccuguagauUcgaauuu........................................................................    6      1    Fm2
....................uacccuguagauccgaUuuu........................................................................    1      1    Fm2
....................uacccuguagauccgaaCuu........................................................................    9      1    Fm2
....................uacccuguagauccgaauGu........................................................................    1      1    Fm2
....................uacccuguagaucUgaauuu........................................................................    1      1    Fm2
....................uUcccuguagauccgaauuu........................................................................    1      1    Fm2
....................uacccuguCgauccgaauuu........................................................................    2      1    Fm2
....................uacccuguagauccgaaGuu........................................................................    13     1    Fm2
....................uacccuguagauccgaauuG........................................................................    5      1    Fm2
....................uaNccuguagauccgaauuu........................................................................    1      1    Fm2
....................uaGccuguagauccgaauuu........................................................................    1      1    Fm2
....................uacccuguagaucAgaauuu........................................................................    1      1    Fm2
....................uacccuguagauccAaauuu........................................................................    1      1    Fm2
....................uacccuguagauccgaauAu........................................................................    10     1    Fm2
....................uacccuguagauccgaaAuu........................................................................    6      1    Fm2
....................uacccuguagauccgaauuA........................................................................    10     1    Fm2
....................uacccuguaUauccgaauuu........................................................................    1      1    Fm2
....................uacccuguaCauccgaauuug.......................................................................    2      1    Fm2
....................Aacccuguagauccgaauuug.......................................................................    3      1    Fm2
....................uacccuguagauccgaauuug.......................................................................    16049  0    Fm2
....................uacccuguagauUcgaauuug.......................................................................    3      1    Fm2
....................uacccCguagauccgaauuug.......................................................................    3      1    Fm2
....................uacccuguagauccgaGuuug.......................................................................    26     1    Fm2
....................uacccuguagauccgCauuug.......................................................................    1      1    Fm2
....................uacccAguagauccgaauuug.......................................................................    3      1    Fm2
....................uacccuNuagauccgaauuug.......................................................................    1      1    Fm2
....................uacccugAagauccgaauuug.......................................................................    2      1    Fm2
....................uaccAuguagauccgaauuug.......................................................................    2      1    Fm2
....................uacccuguagauccgaauuC........................................................................    1      1    Fm2
....................uacccuguagaucAgaauuug.......................................................................    6      1    Fm2
....................uacccuguagauccgaaGuug.......................................................................    82     1    Fm2
....................uacccuguagauccgGauuug.......................................................................    4      1    Fm2
....................uaccUuguagauccgaauuug.......................................................................    2      1    Fm2
....................uacccuguagauccgaauuuU.......................................................................    21     1    Fm2
....................uacccuguUgauccgaauuug.......................................................................    1      1    Fm2
....................uacccuguCgauccgaauuug.......................................................................    1      1    Fm2
....................uacccuguagaCccgaauuug.......................................................................    2      1    Fm2
....................uacGcuguagauccgaauuug.......................................................................    3      1    Fm2
....................uacccuguagauccgaauuGg.......................................................................    1      1    Fm2
....................uacccuguagauccgaauCug.......................................................................    2      1    Fm2
....................uacccuguagaucGgaauuug.......................................................................    3      1    Fm2
....................uacccuguagauccgaCuuug.......................................................................    48     1    Fm2
....................uacccuguagUuccgaauuug.......................................................................    12     1    Fm2
....................uacccuguagauccgaaCuug.......................................................................    97     1    Fm2
```

```
                    Mature                                      Star
agaguucgcccaucagcagauacccuguagauccgaauuuguuugugaucgauaugacaaauuugaauuugcagcgggaucugaaguuggguauaaucaacugacaggagga
....................uacccuguagauccgaUuuug.......................................................................	19	1	Fm2
....................uacUcuguagauccgaauuug.......................................................................	2	1	Fm2
....................uacccugCagauccgaauuug.......................................................................	1	1	Fm2
....................uacccNguagauccgaauuug.......................................................................	1	1	Fm2
....................uacccuAuagauccgaauuug.......................................................................	4	1	Fm2
....................Gacccuguagauccgaauuug.......................................................................	1	1	Fm2
....................Nacccuguagauccgaauuug.......................................................................	4	1	Fm2
....................uUcccuguagauccgaauuug.......................................................................	2	1	Fm2
....................uaAccuguagauccgaauuug.......................................................................	1	1	Fm2
....................uaGccuguagauccgaauuug.......................................................................	5	1	Fm2
....................uacccuguagaucUgaauuug.......................................................................	16	1	Fm2
....................uacccuguaAauccgaauuug.......................................................................	1	1	Fm2
....................uacccuguagGuccgaauuug.......................................................................	17	1	Fm2
....................uacccuguagauccCaauuug.......................................................................	1	1	Fm2
....................uacccuguagCuccgaauuug.......................................................................	24	1	Fm2
....................uacccuguaUauccgaauuug.......................................................................	3	1	Fm2
....................uacccuguagauGcgaauuug.......................................................................	1	1	Fm2
....................uacccuguagauccgaauuuA.......................................................................	103	1	Fm2
....................uCcccuguagauccgaauuug.......................................................................	1	1	Fm2
....................uacccuUuagauccgaauuug.......................................................................	3	1	Fm2
....................uacccuguagauccgaaAuug.......................................................................	79	1	Fm2
....................uacccuguGgauccgaauuug.......................................................................	6	1	Fm2
....................uacAcuguagauccgaauuug.......................................................................	5	1	Fm2
....................uaccGuguagauccgaauuug.......................................................................	1	1	Fm2
....................Cacccuguagauccgaauuug.......................................................................	3	1	Fm2
....................uacccuguagaGccgaauuug.......................................................................	1	1	Fm2
....................uacccuCuagauccgaauuug.......................................................................	3	1	Fm2
....................uaUccuguagauccgaauuug.......................................................................	5	1	Fm2
....................uGcccuguagauccgaauuug.......................................................................	7	1	Fm2
....................uacccugGagauccgaauuug.......................................................................	1	1	Fm2
....................uacccuguagauccgaauAug.......................................................................	1	1	Fm2
....................uacccuguagauccgaauuCg.......................................................................	3	1	Fm2
....................uacccuguagauccgUauuug.......................................................................	2	1	Fm2
....................uacccuguagauAcgaauuugu......................................................................	174	1	Fm2
....................uacccuguagauccgaauNugu......................................................................	1	1	Fm2
....................uGcccuguagauccgaauuugu......................................................................	474	1	Fm2
....................Cacccuguagauccgaauuugu......................................................................	402	1	Fm2
....................uacccuguagaAccgaauuugu......................................................................	219	1	Fm2
....................uacccuguagauccgaauAugu......................................................................	84	1	Fm2
....................uacccuguaCauccgaauuugu......................................................................	81	1	Fm2
....................uacccuguagauccgaGuuugu......................................................................	3678	1	Fm2
....................uacccuguagaCccgaauuugu......................................................................	275	1	Fm2
....................uCcccuguagauccgaauuugu......................................................................	129	1	Fm2
....................uacccuguagGuccgaauuugu......................................................................	2372	1	Fm2
....................uacAcuguagauccgaauuugu......................................................................	922	1	Fm2
....................uacccuguagauccgaauuuCu......................................................................	92	1	Fm2
....................uacccuUuagauccgaauuugu......................................................................	180	1	Fm2
....................uacccuguagauccgaaAuugu......................................................................	9750	1	Fm2
....................uacccuguagauccgaaNuugu......................................................................	2	1	Fm2
....................uacccuguagauccgaauuugC......................................................................	348	1	Fm2
....................uaccAuguagauccgaauuugu......................................................................	636	1	Fm2
....................uacccAguagauccgaauuugu......................................................................	145	1	Fm2
....................uacccuguaUauccgaauuugu......................................................................	221	1	Fm2
....................uacccuguagauccgaNuuugu......................................................................	1	1	Fm2
....................uacccuCuagauccgaauuugu......................................................................	160	1	Fm2
....................uacccGguagauccgaauuugu......................................................................	103	1	Fm2
....................uacUcuguagauccgaauuugu......................................................................	455	1	Fm2
....................uacccuguagaucUgaauuugu......................................................................	2242	1	Fm2
....................uacccuguUgauccgaauuugu......................................................................	335	1	Fm2
....................uacccugCagauccgaauuugu......................................................................	279	1	Fm2
....................uacccuguagaGccgaauuugu......................................................................	42	1	Fm2
....................uaGccuguagauccgaauuugu......................................................................	538	1	Fm2
....................uaccGuguagauccgaauuugu......................................................................	139	1	Fm2
....................uacccuguagauccgaauuugu......................................................................	2140328	0	Fm2
....................uacGcuguagauccgaauuugu......................................................................	232	1	Fm2
....................Aacccuguagauccgaauuugu......................................................................	186	1	Fm2
....................uNcccuguagauccgaauuugu......................................................................	8	1	Fm2
....................uacccuguagCuccgaauuugu......................................................................	2086	1	Fm2
....................uacccuguagauccgaauuugA......................................................................	1061	1	Fm2
....................uacccuguagauccgaauuGgu......................................................................	56	1	Fm2
```

```
                    Mature                                  Star
agaguucgcccaucagcagauacccuguagauccgaauuuguuugugaucgauaugacaaauuugaauuugcagcgggaucugaaguuggguauaaucaacugacaggagga
....................uacccuguagauccUaauuugu......................................................................   222     1   Fm2
....................uaccUuguagauccgaauuugu......................................................................   299     1   Fm2
....................uacccNguagauccgaauuugu......................................................................   356     1   Fm2
....................uacccuguagauGcgaauuugu......................................................................   73      1   Fm2
....................uacccuguagaucAgaauuugu......................................................................   724     1   Fm2
....................uacccugGagauccgaauuugu......................................................................   57      1   Fm2
....................Gacccuguagauccgaauuugu......................................................................   38      1   Fm2
....................uacccuguagauccgaaCuugu......................................................................   12994   1   Fm2
....................uacccuguagauccgUauuugu......................................................................   538     1   Fm2
....................uacccuguagauccCaauuugu......................................................................   77      1   Fm2
....................uacccuguagauccgaauCugu......................................................................   200     1   Fm2
....................uaUccuguagauccgaauuugu......................................................................   414     1   Fm2
....................uaNccuguagauccgaauuugu......................................................................   10      1   Fm2
....................uacccuguagauUcgaauuugu......................................................................   857     1   Fm2
....................uUcccuguagauccgaauuugu......................................................................   390     1   Fm2
....................uacccuguGgauccgaauuugu......................................................................   279     1   Fm2
....................uacccuAuagauccgaauuugu......................................................................   287     1   Fm2
....................uacccuguagauccgCauuugu......................................................................   188     1   Fm2
....................uacccuguagauccgGauuugu......................................................................   273     1   Fm2
....................uacccuguagauccgaauGugu......................................................................   52      1   Fm2
....................uacccuguagUuccgaauuugu......................................................................   1811    1   Fm2
....................uacccuguagauccgaauuAgu......................................................................   152     1   Fm2
....................uacccuNuagauccgaauuugu......................................................................   132     1   Fm2
....................uacccuguagauccAaauuugu......................................................................   313     1   Fm2
....................Nacccuguagauccgaauuugu......................................................................   1019    1   Fm2
....................uacccuguagauccgaauuugG......................................................................   9110    1   Fm2
....................uacccugAagauccgaauuugu......................................................................   166     1   Fm2
....................uacccuguagauccgaauuuAu......................................................................   267     1   Fm2
....................uacccuguCgauccgaauuugu......................................................................   76      1   Fm2
....................uacccuguagauccgaCuuugu......................................................................   5380    1   Fm2
....................uacccuguagauccgaaGuugu......................................................................   11187   1   Fm2
....................uacccCguagauccgaauuugu......................................................................   257     1   Fm2
....................uaAccuguagauccgaauuugu......................................................................   474     1   Fm2
....................uacccuguaAauccgaauuugu......................................................................   130     1   Fm2
....................uacccuguagauccgaUuuugu......................................................................   3224    1   Fm2
....................uacccuguagauccgaauuuUu......................................................................   367     1   Fm2
....................uacccuguagauccgaauuCgu......................................................................   195     1   Fm2
....................uacccuguagaucGgaauuugu......................................................................   143     1   Fm2
....................uacccuguagauccgUauuuguu.....................................................................   18      1   Fm2
....................uacccuguCgauccgaauuuguu.....................................................................   3       1   Fm2
....................uGcccuguagauccgaauuuguu.....................................................................   12      1   Fm2
....................Aacccuguagauccgaauuuguu.....................................................................   7       1   Fm2
....................uacccuguagauccgCauuuguu.....................................................................   8       1   Fm2
....................uCcccuguagauccgaauuuguu.....................................................................   5       1   Fm2
....................uaccUuguagauccgaauuuguu.....................................................................   6       1   Fm2
....................uacccuguagUuccgaauuuguu.....................................................................   37      1   Fm2
....................uacccuguagauccgaaCuuguu.....................................................................   253     1   Fm2
....................uacccuguagauccgaauuCguu.....................................................................   4       1   Fm2
....................uacccuguagauccgaCuuuguu.....................................................................   131     1   Fm2
....................uacccuguGgauccgaauuuguu.....................................................................   8       1   Fm2
....................uacccuguagauccgaauuuCuu.....................................................................   2       1   Fm2
....................uacccugGagauccgaauuuguu.....................................................................   1       1   Fm2
....................uacccuCuagauccgaauuuguu.....................................................................   8       1   Fm2
....................uacccuUuagauccgaauuuguu.....................................................................   3       1   Fm2
....................uacccuguagauccgaUuuuguu.....................................................................   62      1   Fm2
....................uaccAuguagauccgaauuuguu.....................................................................   18      1   Fm2
....................uacccuguagauccgaauuuguu.....................................................................   44301   0   Fm2
....................uacccuguaAauccgaauuuguu.....................................................................   2       1   Fm2
....................uacccuguagauccgaauuuguA.....................................................................   5976    1   Fm2
....................uacccuguagauccgaauuugAu.....................................................................   3       1   Fm2
....................Cacccuguagauccgaauuuguu.....................................................................   3       1   Fm2
....................uUcccuguagauccgaauuuguu.....................................................................   7       1   Fm2
....................uaUccuguagauccgaauuuguu.....................................................................   7       1   Fm2
....................uacccuguagaucUgaauuuguu.....................................................................   167     1   Fm2
....................uacUcuguagauccgaauuuguu.....................................................................   11      1   Fm2
....................uacccuguagauccgGauuuguu.....................................................................   3       1   Fm2
....................uacccuguUgauccgaauuuguu.....................................................................   3       1   Fm2
....................uacccuguagauUcgaauuuguu.....................................................................   19      1   Fm2
....................uacccuguagauccgaauCuguu.....................................................................   2       1   Fm2
....................uacccuguagaucAgaauuuguu.....................................................................   9       1   Fm2
```

```
                    Mature                                 Star
agaguucgcccaucagcagauacccuguagauccgaauuuguuugugaucgauaugacaaauuugaauuugcagcgggaucugaaguuggguauaaucaacugacaggagga
....................uacccugCagauccgaauuuguu.....................................................................    6      1   Fm2
....................uacccuguagauGcgaauuuguu.....................................................................    2      1   Fm2
....................uacccuguagaCccgaauuuguu.....................................................................    8      1   Fm2
....................uacccuguaUauccgaauuuguu.....................................................................    1      1   Fm2
....................uacccuguagauccgaauuuAuu.....................................................................    3      1   Fm2
....................uacccuguagauccCaauuuguu.....................................................................    1      1   Fm2
....................uacccuguagauAcgaauuuguu.....................................................................    4      1   Fm2
....................uacccuguagauccgaauuGguu.....................................................................    3      1   Fm2
....................uacccuguagauccUaauuuguu.....................................................................    8      1   Fm2
....................uaAccuguagauccgaauuuguu.....................................................................    15     1   Fm2
....................uacccuguagauccgaauuAguu.....................................................................    2      1   Fm2
....................uacccCguagauccgaauuuguu.....................................................................    7      1   Fm2
....................uacccuguagGuccgaauuuguu.....................................................................    44     1   Fm2
....................uacccGguagauccgaauuuguu.....................................................................    4      1   Fm2
....................uacccuguagaucGgaauuuguu.....................................................................    4      1   Fm2
....................uacccuNuagauccgaauuuguu.....................................................................    1      1   Fm2
....................Gacccuguagauccgaauuuguu.....................................................................    2      1   Fm2
....................uacccuguagauccgaauuugCu.....................................................................    1      1   Fm2
....................uacAcuguagauccgaauuuguu.....................................................................    26     1   Fm2
....................uacccuguagauccAaauuuguu.....................................................................    4      1   Fm2
....................uacccuguagaAccgaauuuguu.....................................................................    2      1   Fm2
....................uacccNguagauccgaauuuguu.....................................................................    9      1   Fm2
....................uacGcuguagauccgaauuuguu.....................................................................    5      1   Fm2
....................uacccuguagauccgaGuuuguu.....................................................................    59     1   Fm2
....................uacccAguagauccgaauuuguu.....................................................................    2      1   Fm2
....................uacccuguagauccgaauuuUuu.....................................................................    2      1   Fm2
....................uacccuAuagauccgaauuuguu.....................................................................    4      1   Fm2
....................uacccuguagauccgaauuuguC.....................................................................    25     1   Fm2
....................uacccuguagauccgaauuuguG.....................................................................    34     1   Fm2
....................uacccuguagCuccgaauuuguu.....................................................................    50     1   Fm2
....................uaGccuguagauccgaauuuguu.....................................................................    13     1   Fm2
....................uacccuguagaGccgaauuuguu.....................................................................    1      1   Fm2
....................Nacccuguagauccgaauuuguu.....................................................................    25     1   Fm2
....................uacccuguagauccgaaAuuguu.....................................................................    189    1   Fm2
....................uacccugAagauccgaauuuguu.....................................................................    6      1   Fm2
....................uacccuguagauccgaaGuuguu.....................................................................    240    1   Fm2
....................uacccuguagauccgaauuuguuA....................................................................    611    1   Fm2
....................uacccuguagauccgaaAuuguuu....................................................................    17     1   Fm2
....................uacccuguagauccgaaGuuguuu....................................................................    17     1   Fm2
....................uacccuguagauccgaaCuuguuu....................................................................    13     1   Fm2
....................uacccuguagauccgaGuuuguuu....................................................................    4      1   Fm2
....................uacccuguagauccgaauuuguuu....................................................................    2585   0   Fm2
....................uaccAuguagauccgaauuuguuu....................................................................    1      1   Fm2
....................uacccuguagaucAgaauuuguuu....................................................................    2      1   Fm2
....................uacccuguagauccgaauuuguAu....................................................................    2668   1   Fm2
....................Cacccuguagauccgaauuuguuu....................................................................    1      1   Fm2
....................uacccuguagauccgaauuuguGu....................................................................    33     1   Fm2
....................uacccuguagauccgaauuuguCu....................................................................    22     1   Fm2
....................uacccuguagGuccgaauuuguuu....................................................................    1      1   Fm2
....................uacccuguagauccgUauuuguuu....................................................................    1      1   Fm2
....................uacccuguagaucUgaauuuguuu....................................................................    14     1   Fm2
....................uacccuguagauccgaauuugCuu....................................................................    1      1   Fm2
....................uacccuguagauccgaCuuuguuu....................................................................    5      1   Fm2
....................uacccuguagauGcgaauuuguuu....................................................................    1      1   Fm2
....................uacccuguagCuccgaauuuguuu....................................................................    2      1   Fm2
....................uacAcuguagauccgaauuuguuu....................................................................    4      1   Fm2
....................uacccuguagauccgaUuuuguuu....................................................................    7      1   Fm2
....................uacccNguagauccgaauuuguuu....................................................................    2      1   Fm2
....................uacccuguagUuccgaauuuguuu....................................................................    2      1   Fm2
....................Nacccuguagauccgaauuuguuu....................................................................    2      1   Fm2
....................uacccuguagaAccgaauuuguuu....................................................................    1      1   Fm2
....................uacccuguagauccgaauuuguuuU...................................................................    7      1   Fm2
....................uacccuguagauccgaauuuguuug...................................................................    17     0   Fm2
....................uacccuguagauccgaauuuguuugu..................................................................    7      0   Fm2
....................uacccuguagauccgaauuuguuuguga................................................................    6      0   Fm2
....................uacccuguagauccgaauuuguuugugG................................................................    2      1   Fm2
....................uacccuguagauccgaauuuguuugugU................................................................    14     1   Fm2
....................uacccuguagauccgaauuuguuugugau...............................................................    2      0   Fm2
.....................acccuguagauccgaauu.........................................................................    7      0   Fm2
.....................acccuguagauccgaauC.........................................................................    1      1   Fm2
```

```
                     Mature                                      Star
agaguucgcccaucagcagauacccuguagauccgaauuuguuugugaucgauaugacaaauuugaauuugcagcgggaucugaaguuggguauaaucaacugacaggagga
.....................acccuguagauccgaauuu........................................................................ 5     0 Fm2
.....................acccuguagaucUgaauuu........................................................................ 1     1 Fm2
.....................acccuguaAauccgaauuug....................................................................... 1     1 Fm2
.....................acccuguagauccgaauuug....................................................................... 4     0 Fm2
.....................acccuUuagauccgaauuugu...................................................................... 1     1 Fm2
.....................acccuguagauccgaGuuugu...................................................................... 6     1 Fm2
.....................acccuguagaucAgaauuugu...................................................................... 3     1 Fm2
.....................acccuguagGuccgaauuugu...................................................................... 2     1 Fm2
.....................acccuguagUuccgaauuugu...................................................................... 3     1 Fm2
.....................acccuguagauccgaauuuUu...................................................................... 14    1 Fm2
.....................acccuguaCauccgaauuugu...................................................................... 1     1 Fm2
.....................acccuguagauUcgaauuugu...................................................................... 3     1 Fm2
.....................acccuguagauccgaauuuCu...................................................................... 10    1 Fm2
.....................acccugAagauccgaauuugu...................................................................... 1     1 Fm2
.....................acccuguagauccgaauuAgu...................................................................... 1     1 Fm2
.....................acccuguagaGccgaauuugu...................................................................... 10    1 Fm2
.....................acccuguagauGcgaauuugu...................................................................... 1     1 Fm2
.....................acccuguagaucGgaauuugu...................................................................... 1     1 Fm2
.....................aAccuguagauccgaauuugu...................................................................... 5     1 Fm2
.....................acccuguagauccUaauuugu...................................................................... 2     1 Fm2
.....................acAcuguagauccgaauuugu...................................................................... 2     1 Fm2
.....................acccugGagauccgaauuugu...................................................................... 1     1 Fm2
.....................acccuguagaAccgaauuugu...................................................................... 20    1 Fm2
.....................accGuguagauccgaauuugu...................................................................... 1     1 Fm2
.....................accAuguagauccgaauuugu...................................................................... 5     1 Fm2
.....................Ucccuguagauccgaauuugu...................................................................... 8     1 Fm2
.....................acccuguagauccgaauGugu...................................................................... 19    1 Fm2
.....................acccuguagauccgCauuugu...................................................................... 6     1 Fm2
.....................acccAguagauccgaauuugu...................................................................... 1     1 Fm2
.....................acGcuguagauccgaauuugu...................................................................... 3     1 Fm2
.....................acccuguagaucUgaauuugu...................................................................... 7     1 Fm2
.....................acccuguUgauccgaauuugu...................................................................... 2     1 Fm2
.....................acccuguagauccgaauuuAu...................................................................... 10    1 Fm2
.....................acccuguagauccgaUuuugu...................................................................... 2     1 Fm2
.....................acccuguagauccgaauuGgu...................................................................... 2     1 Fm2
.....................acccuguaAauccgaauuugu...................................................................... 2     1 Fm2
.....................aUccuguagauccgaauuugu...................................................................... 1     1 Fm2
.....................acccuguagauccgaaCuugu...................................................................... 2     1 Fm2
.....................acccuNuagauccgaauuugu...................................................................... 1     1 Fm2
.....................acccuguagauccAaauuugu...................................................................... 2     1 Fm2
.....................acccuAuagauccgaauuugu...................................................................... 1     1 Fm2
.....................acccuguagCuccgaauuugu...................................................................... 2     1 Fm2
.....................acccuguagauccCaauuugu...................................................................... 2     1 Fm2
.....................acccugCagauccgaauuugu...................................................................... 8     1 Fm2
.....................acccuguagauccgGauuugu...................................................................... 1     1 Fm2
.....................acccuguagauccgaauAugu...................................................................... 13    1 Fm2
.....................acUcuguagauccgaauuugu...................................................................... 1     1 Fm2
.....................acccCguagauccgaauuugu...................................................................... 3     1 Fm2
.....................acccuguaUauccgaauuugu...................................................................... 4     1 Fm2
.....................acccuguagauccgaauuCgu...................................................................... 1     1 Fm2
.....................acccuguagauccgaCuuugu...................................................................... 9     1 Fm2
.....................acccuguagauccgaaAuugu...................................................................... 1     1 Fm2
.....................acccGguagauccgaauuugu...................................................................... 3     1 Fm2
.....................acccuguagauccgaauuugu...................................................................... 5446  0 Fm2
.....................acccuguagauccgaauCugu...................................................................... 31    1 Fm2
.....................acccuguCgauccgaauuugu...................................................................... 1     1 Fm2
.....................acccuguagauccgaauuugG...................................................................... 35    1 Fm2
.....................acccuguagauccgaauuugA...................................................................... 1     1 Fm2
.....................Ncccuguagauccgaauuugu...................................................................... 1     1 Fm2
.....................acccuguagauccgUauuugu...................................................................... 5     1 Fm2
.....................acccuguagaCccgaauuugu...................................................................... 4     1 Fm2
.....................acccuCuagauccgaauuugu...................................................................... 2     1 Fm2
.....................acccuguagauccgaauuuguA..................................................................... 18    1 Fm2
.....................acccuguagaCccgaauuuguu..................................................................... 1     1 Fm2
.....................acccAguagauccgaauuuguu..................................................................... 1     1 Fm2
.....................acccuguagauccgaauGuguu..................................................................... 1     1 Fm2
.....................acccuguagauccgaauCuguu..................................................................... 1     1 Fm2
.....................acAcuguagauccgaauuuguu..................................................................... 1     1 Fm2
.....................acccuguagauccgaauuuguu..................................................................... 241   0 Fm2
.....................acccuguagauccgaaGuuguu..................................................................... 2     1 Fm2
```

Mature **Star**

agaguucgcccaucagcagauacccuguagauccgaauuuguuugugaucgauaugacaaauuugaauuugcagcgggaucugaaguuggguauaaucaacugacaggagga			
.....................acccuguCgauccgaauuuguu...	1	1	Fm2
.....................acccuguagauccgaauuuguu...	17	0	Fm2
.....................acccuguagauccgaauCuguu...	1	1	Fm2
.....................acccuguagauccgaauuuguAu..	9	1	Fm2
......................cccugAagauccgaauuug...	1	1	Fm2
......................Accuguagauccgaauuugu..	1	1	Fm2
......................cccuguagGuccgaauuugu..	1	1	Fm2
......................cccuguagauccgaauuugu..	161	0	Fm2
......................cccuguagauccgaauuAgu..	1	1	Fm2
......................cccuguagaucGgaauuugu..	2	1	Fm2
......................ccGuguagauccgaauuugu..	1	1	Fm2
......................cccuguagUuccgaauuugu..	2	1	Fm2
......................cccuguagauccgaUuuugu..	1	1	Fm2
......................cccuguagauccgaauuCgu..	1	1	Fm2
......................cccuguagauccgaauuuUu..	17	1	Fm2
......................Uccuguagauccgaauuugu..	4	1	Fm2
......................cccuguagauccgaauGuguu...	1	1	Fm2
......................cccuguagauccgaauuuguu...	5	0	Fm2
.......................ccuguagauccgaauuugu..	22	0	Fm2
........................cuguagauccgaauuugu..	7	0	Fm2
........................cuguagaucUgaauuugu..	1	1	Fm2
........................cuguagauccgaauuugC..	1	1	Fm2
....guugugaucgauaugacaaauuugaauuugcagcgggaCc...............................	1	1	Fm2
....uugugaucgauaugaca...	3	0	Fm2
....aaauuugaauuugcagcgggauc...............................	31	0	Fm2
....aaauCugaauuugcagcgggauc...............................	1	1	Fm2
....aaauuNgaauuugcagcgggauc...............................	1	1	Fm2
....aaauuugaauuugcagcAggauc...............................	1	1	Fm2
....aaauuugaauuugcagcgggaCc...............................	54	1	Fm2
....aauuugaauuugcagcgggaCc...............................	7	1	Fm2
..................gGuacccuguagauccgaauuugu..	6	1	Fm1
...................auacccuguagaAccgaa...	21	1	Fm1
...................auacccuguagaAccgaau..	32	1	Fm1
...................Cuacccuguagauccgaau..	2	1	Fm1
...................Cuacccuguagauccgaauuug...	4	1	Fm1
...................auacccuguagauccgaaCuugu..	1	1	Fm1
...................auacccuguagaAccgaauuugu..	4	1	Fm1
...................auacccuguagauccgaauuugu..	11	0	Fm1
...................Cuacccuguagauccgaauuugu..	190	1	Fm1
...................Cuacccuguagauccgaauuuguu...	2	1	Fm1
....................Aacccuguagauccgaau..	2	1	Fm1
....................uacccuguagauccgGau..	1	1	Fm1
....................uacccuguagaCccgaau..	1	1	Fm1
....................uacccuguagauccgaaC..	26	1	Fm1
....................uacccuguUgauccgaau..	1	1	Fm1
....................uacccuguagauccgaUu..	5	1	Fm1
....................uacccuguagUuccgaau..	2	1	Fm1
....................uacccuguagGuccgaau..	3	1	Fm1
....................uacccuguagauccgaaA..	33	1	Fm1
....................uacccuAuagauccgaau..	1	1	Fm1
....................uacccuguagauccgaGu..	2	1	Fm1
....................uacccuguagauccgaCu..	6	1	Fm1
....................uaNccuguagauccgaau..	1	1	Fm1
....................uacccuguagaucUgaau..	5	1	Fm1
....................uacccuguagauccgaaG..	12	1	Fm1
....................uacccuguagaAccgaau..	11	1	Fm1
....................uacccuguagauUcgaau..	16	1	Fm1
....................uacccuguagauccgaau..	2119	0	Fm1
....................Cacccuguagauccgaau..	1	1	Fm1
....................uacUcuguagauccgaau..	1	1	Fm1
....................uaccGuguagauccgaau..	1	1	Fm1
....................uacccuguagCuccgaau..	3	1	Fm1
....................uacAcuguagauccgaau..	1	1	Fm1
....................uacccGguagauccgaau..	1	1	Fm1
....................uGcccuguagauccgaau..	2	1	Fm1
....................uacccuguagaucAgaau..	2	1	Fm1
....................uacccNguagauccgaauu...	1	1	Fm1
....................uacccuguagaucGgaauu...	1	1	Fm1
....................uacccugCagauccgaauu...	2	1	Fm1

```
                    Mature                              Star
agaguucgcccaucagcagauacccuguagauccgaauuuguuugugaucgauaugacaaauuugaauuugcagcgggaucugaaguuggguauaaucaacugacaggagga
....................uacccuguagauccgaaCu.........................................................................   28     1   Fm1
....................uacccCguagauccgaauu.........................................................................   2      1   Fm1
....................uacccuguagauccgaauC.........................................................................   2      1   Fm1
....................uacccuguagauccgaaAu.........................................................................   37     1   Fm1
....................uaGccuguagauccgaauu.........................................................................   1      1   Fm1
....................uacccuNuagauccgaauu.........................................................................   1      1   Fm1
....................uacccuUuagauccgaauu.........................................................................   2      1   Fm1
....................uacccuguagUuccgaauu.........................................................................   6      1   Fm1
....................uacccuCuagauccgaauu.........................................................................   1      1   Fm1
....................uacccuguagauccgUauu.........................................................................   4      1   Fm1
....................uacccuguagCuccgaauu.........................................................................   6      1   Fm1
....................uacccuguagauccgaCuu.........................................................................   9      1   Fm1
....................uacccuguUgauccgaauu.........................................................................   2      1   Fm1
....................uacccuguagauccgGauu.........................................................................   1      1   Fm1
....................uacccuguagaucAgaauu.........................................................................   1      1   Fm1
....................uacccuguagauccgaauA.........................................................................   97     1   Fm1
....................uacccGguagauccgaauu.........................................................................   1      1   Fm1
....................uacccuguagaucUgaauu.........................................................................   4      1   Fm1
....................uacccuguaAauccgaauu.........................................................................   1      1   Fm1
....................uacccuguagauccgaaGu.........................................................................   22     1   Fm1
....................uaccUuguagauccgaauu.........................................................................   2      1   Fm1
....................uacccuguaCauccgaauu.........................................................................   1      1   Fm1
....................uacccuguagauccgaUuu.........................................................................   3      1   Fm1
....................uacccuguagauccgaauG.........................................................................   1      1   Fm1
....................uGcccuguagauccgaauu.........................................................................   1      1   Fm1
....................uacccugAagauccgaauu.........................................................................   1      1   Fm1
....................uacccuAuagauccgaauu.........................................................................   2      1   Fm1
....................uacccuguagauUcgaauu.........................................................................   17     1   Fm1
....................uacccuguagaAccgaauu.........................................................................   1      1   Fm1
....................uacccuguagauccgaauu.........................................................................   4643   0   Fm1
....................uacGcuguagauccgaauu.........................................................................   2      1   Fm1
....................uacccuguagauccgaGuu.........................................................................   4      1   Fm1
....................uacUcuguagauccgaauu.........................................................................   3      1   Fm1
....................Cacccuguagauccgaauu.........................................................................   3      1   Fm1
....................uacccuguagGuccgaauu.........................................................................   4      1   Fm1
....................uacccuguagauccgaCuuu........................................................................   2      1   Fm1
....................uacccuguagauccgaauGu........................................................................   1      1   Fm1
....................uacccuUuagauccgaauuu........................................................................   1      1   Fm1
....................uaUccuguagauccgaauuu........................................................................   1      1   Fm1
....................Aacccuguagauccgaauuu........................................................................   1      1   Fm1
....................uacccuguagauccgaaGuu........................................................................   12     1   Fm1
....................uacccuguagGuccgaauuu........................................................................   3      1   Fm1
....................uacccuguagauccgaGuuu........................................................................   5      1   Fm1
....................uaccAuguagauccgaauuu........................................................................   1      1   Fm1
....................uacccuguagauUcgaauuu........................................................................   5      1   Fm1
....................Nacccuguagauccgaauuu........................................................................   1      1   Fm1
....................uacccuguagauccgaauuC........................................................................   1      1   Fm1
....................uacccuguagauccgaauAu........................................................................   2      1   Fm1
....................uacccuguagaucUgaauuu........................................................................   19     1   Fm1
....................uaGccuguagauccgaauuu........................................................................   2      1   Fm1
....................uacccuguagauAcgaauuu........................................................................   1      1   Fm1
....................uacccuguagUuccgaauuu........................................................................   1      1   Fm1
....................uacccuguagauccgaauuu........................................................................   2300   0   Fm1
....................uacccuguagauccgaaAuu........................................................................   11     1   Fm1
....................uaAccuguagauccgaauuu........................................................................   2      1   Fm1
....................uacccuguagauccgaauuA........................................................................   10     1   Fm1
....................uacccuguagauccgaaCuu........................................................................   11     1   Fm1
....................uacccuCuagauccgaauuu........................................................................   1      1   Fm1
....................uacccuguagauccgaUuuu........................................................................   5      1   Fm1
....................uaccUuguagauccgaauuu........................................................................   1      1   Fm1
....................uacccuguagauccgaauuuU.......................................................................   22     1   Fm1
....................uaccUuguagauccgaauuug.......................................................................   1      1   Fm1
....................Cacccuguagauccgaauuug.......................................................................   2      1   Fm1
....................uacccuAuagauccgaauuug.......................................................................   1      1   Fm1
....................uacccuguagauccgaauuGg.......................................................................   2      1   Fm1
....................uacccuguagauccgCauuug.......................................................................   1      1   Fm1
....................uacccuguagCuccgaauuug.......................................................................   7      1   Fm1
....................uUcccuguagauccgaauuug.......................................................................   3      1   Fm1
....................uacccuguagaucAgaauuug.......................................................................   3      1   Fm1
....................uacccuguagauccgaaGuug.......................................................................   36     1   Fm1
```

Mature Star

agaguucgcccaucagcagauacccuguagauccgaauuuguuugugaucgauaugacaaauuugaauuugcagcgggaucugaaguuggguauaaucaacugacaggagga

....................uacccuguagauccgaaAuug...	36	1	Fm1
....................uacccuguagauccgaCuuug...	18	1	Fm1
....................uacccuguagauccgaauuCg...	3	1	Fm1
....................uacccCguagauccgaauuug...	1	1	Fm1
....................uacccuguagauccgaauuug...	7071	0	Fm1
....................uaGccuguagauccgaauuug...	5	1	Fm1
....................uacUcuguagauccgaauuug...	4	1	Fm1
....................uacccuguagauccgaauCug...	1	1	Fm1
....................uacccuguagauccgaauuuC...	1	1	Fm1
....................uaccAuguagauccgaauuug...	1	1	Fm1
....................uaccGuguagauccgaauuug...	1	1	Fm1
....................uacccuguagUuccgaauuug...	4	1	Fm1
....................uacccuguagauccgaaCuug...	56	1	Fm1
....................uacccuguagauccgaauuuA...	31	1	Fm1
....................uacccuguagauccgaUuuug...	11	1	Fm1
....................uacccuguaUauccgaauuug...	1	1	Fm1
....................uCcccuguagauccgaauuug...	3	1	Fm1
....................Aacccuguagauccgaauuug...	1	1	Fm1
....................uacccuguagauccgGauuug...	3	1	Fm1
....................uacccuguagaucUgaauuug...	22	1	Fm1
....................uacGcuguagauccgaauuug...	2	1	Fm1
....................uaUccuguagauccgaauuug...	3	1	Fm1
....................uacccuguagauccgaGuuug...	11	1	Fm1
....................uGcccuguagauccgaauuug...	4	1	Fm1
....................uacccugCagauccgaauuug...	3	1	Fm1
....................Nacccuguagauccgaauuug...	1	1	Fm1
....................uacccuguagGuccgaauuug...	9	1	Fm1
....................uacAcuguagauccgaauuug...	2	1	Fm1
....................uacccuguagCuccgaauuugu..	968	1	Fm1
....................uacccuguagauGcgaauuugu..	25	1	Fm1
....................uacccuguUgauccgaauuugu..	190	1	Fm1
....................uacccuguagauccgaauuCgu..	100	1	Fm1
....................uacccuguagauccgaNuuugu..	4	1	Fm1
....................uacccuAuagauccgaauuugu..	89	1	Fm1
....................uaUccuguagauccgaauuugu..	222	1	Fm1
....................uacccuguagauccgUauuugu..	322	1	Fm1
....................uacccuguagaucAgaauuugu..	236	1	Fm1
....................uacccuguagauccgaauuAgu..	89	1	Fm1
....................uacccuguagauccgaauCugu..	100	1	Fm1
....................uUcccuguagauccgaauuugu..	192	1	Fm1
....................uaAccuguagauccgaauuugu..	218	1	Fm1
....................uacccuguagauccgaauuuCu..	23	1	Fm1
....................uacccuguagauccgaauGugu..	54	1	Fm1
....................uacccuguaAauccgaauuugu..	67	1	Fm1
....................uacccuguagauAcgaauuugu..	45	1	Fm1
....................uacccuguagauccgaauuGgu..	37	1	Fm1
....................Gacccuguagauccgaauuugu..	22	1	Fm1
....................uCcccuguagauccgaauuugu..	64	1	Fm1
....................uacccuguagaucUgaauuugu..	1099	1	Fm1
....................uacccugAagauccgaauuugu..	62	1	Fm1
....................uacccuguagauccCaauuugu..	29	1	Fm1
....................uacGcuguagauccgaauuugu..	111	1	Fm1
....................uacccugCagauccgaauuugu..	125	1	Fm1
....................Nacccuguagauccgaauuugu..	522	1	Fm1
....................uacccuguagaucGgaauuugu..	62	1	Fm1
....................uacccuguagauccgaauuugu..	1001816	0	Fm1
....................uaccUuguagauccgaauuugu..	176	1	Fm1
....................uacccuguagaCccgaauuugu..	141	1	Fm1
....................uGcccuguagauccgaauuugu..	213	1	Fm1
....................uacccuguCgauccgaauuugu..	30	1	Fm1
....................uacccuguagauccgaaGuugu..	5303	1	Fm1
....................uacccuNuagauccgaauuugu..	53	1	Fm1
....................uNcccuguagauccgaauuugu..	5	1	Fm1
....................uacccuguaCauccgaauuugu..	41	1	Fm1
....................uacccuguagauccgaUuuugu..	1728	1	Fm1
....................uacccGguagauccgaauuugu..	53	1	Fm1
....................uaccAuguagauccgaauuugu..	161	1	Fm1
....................uacccCguagauccgaauuugu..	115	1	Fm1
....................uacUcuguagauccgaauuugu..	193	1	Fm1
....................Cacccuguagauccgaauuugu..	212	1	Fm1

```
                    Mature                                    Star
agaguucgcccaucagcagauacccuguagauccgaauuuguuugugaucgauaugacaaauuugaauuugcagcgggaucugaaguuggguauaaucaacugacaggagga
....................uacccuguagauUcgaauuugu......................................................................	587	1	Fm1
....................uacccuguagauccgaauuugC......................................................................	452	1	Fm1
....................uacccAguagauccgaauuugu......................................................................	53	1	Fm1
....................uacccugGagauccgaauuugu......................................................................	24	1	Fm1
....................uacccuguagauccUaauuugu......................................................................	92	1	Fm1
....................uacccuguaUauccgaauuugu......................................................................	84	1	Fm1
....................uacccuCuagauccgaauuugu......................................................................	43	1	Fm1
....................uacccuguagauccgaGuuugu......................................................................	2295	1	Fm1
....................uacccuguagauccgaCuuugu......................................................................	2630	1	Fm1
....................uacAcuguagauccgaauuugu......................................................................	448	1	Fm1
....................uaGccuguagauccgaauuugu......................................................................	215	1	Fm1
....................uacccuguagauccgaaCuugu......................................................................	6435	1	Fm1
....................uacccuguagauccAaauuugu......................................................................	118	1	Fm1
....................uaccGuguagauccgaauuugu......................................................................	60	1	Fm1
....................uacccuguagauccgaauAugu......................................................................	45	1	Fm1
....................uacccuguagaGccgaauuugu......................................................................	20	1	Fm1
....................uacccuguagaAccgaauuugu......................................................................	91	1	Fm1
....................uacccuguagauccgaauuugA......................................................................	461	1	Fm1
....................uaNccuguagauccgaauuugu......................................................................	6	1	Fm1
....................uacccuguagUuccgaauuugu......................................................................	792	1	Fm1
....................uacccuguagauccgGauuugu......................................................................	149	1	Fm1
....................uacccuguagauccgaauuugG......................................................................	3635	1	Fm1
....................uacccuguagauccgaauuuUu......................................................................	80	1	Fm1
....................uacccNguagauccgaauuugu......................................................................	136	1	Fm1
....................uacccuguagauccgCauuugu......................................................................	124	1	Fm1
....................Aacccuguagauccgaauuugu......................................................................	121	1	Fm1
....................uacccuguagauccgaauuuAu......................................................................	79	1	Fm1
....................uacccuguGgauccgaauuugu......................................................................	162	1	Fm1
....................uacccuUuagauccgaauuugu......................................................................	76	1	Fm1
....................uacccuguagauccgaaAuugu......................................................................	4770	1	Fm1
....................uacccuguagGuccgaauuugu......................................................................	1058	1	Fm1
....................uacccuUuagauccgaauuuguu.....................................................................	1	1	Fm1
....................uacccuCuagauccgaauuuguu.....................................................................	2	1	Fm1
....................uacAcuguagauccgaauuuguu.....................................................................	10	1	Fm1
....................uNcccuguagauccgaauuuguu.....................................................................	2	1	Fm1
....................uacccuguagauccgaauuAguu.....................................................................	1	1	Fm1
....................Nacccuguagauccgaauuuguu.....................................................................	10	1	Fm1
....................uacccuguagauccgUauuuguu.....................................................................	3	1	Fm1
....................uacccuAuagauccgaauuuguu.....................................................................	2	1	Fm1
....................uacccuguUgauccgaauuuguu.....................................................................	3	1	Fm1
....................uacccuguagauccgaauuCguu.....................................................................	2	1	Fm1
....................uacccuguagauccgaauuuUuu.....................................................................	2	1	Fm1
....................uacccuguaUauccgaauuuguu.....................................................................	2	1	Fm1
....................uUcccuguagauccgaauuuguu.....................................................................	3	1	Fm1
....................uacccuguCgauccgaauuuguu.....................................................................	3	1	Fm1
....................uacccuNuagauccgaauuuguu.....................................................................	2	1	Fm1
....................uacccuguagauccgaUuuuguu.....................................................................	35	1	Fm1
....................uacccuguagauccgGauuuguu.....................................................................	5	1	Fm1
....................uacccuguagauAcgaauuuguu.....................................................................	2	1	Fm1
....................uacccuguaAauccgaauuuguu.....................................................................	4	1	Fm1
....................uacccuguagaCccgaauuuguu.....................................................................	2	1	Fm1
....................uacccuguagauccgCauuuguu.....................................................................	1	1	Fm1
....................uacccGguagauccgaauuuguu.....................................................................	3	1	Fm1
....................uacccuguagauccgaauuuAuu.....................................................................	1	1	Fm1
....................uacccNguagauccgaauuuguu.....................................................................	3	1	Fm1
....................uCcccuguagauccgaauuuguu.....................................................................	6	1	Fm1
....................uacccugGagauccgaauuuguu.....................................................................	1	1	Fm1
....................uacccugAagauccgaauuuguu.....................................................................	2	1	Fm1
....................uacccuguagauccgaauCuguu.....................................................................	3	1	Fm1
....................uacccuguagauccgaauAuguu.....................................................................	1	1	Fm1
....................uaUccuguagauccgaauuuguu.....................................................................	10	1	Fm1
....................uacccuguagauccgaauuuguG.....................................................................	18	1	Fm1
....................uacccuguagauccgaCuuuguu.....................................................................	69	1	Fm1
....................uacUcuguagauccgaauuuguu.....................................................................	10	1	Fm1
....................uacccuguagauccAaauuuguu.....................................................................	2	1	Fm1
....................uGcccuguagauccgaauuuguu.....................................................................	8	1	Fm1
....................uacccuguagaAccgaauuuguu.....................................................................	2	1	Fm1
....................Cacccuguagauccgaauuuguu.....................................................................	9	1	Fm1
....................uacccuguagauccgaauuGguu.....................................................................	1	1	Fm1
....................Aacccuguagauccgaauuuguu.....................................................................	3	1	Fm1
```

```
                    Mature                                    Star
agaguucgcccaucagcagauacccuguagauccgaauuuguuugugaucgauaugacaaauuugaauuugcagcgggaucugaaguuggguauaaucaacugacaggagga
....................uacccuguagauUcgaauuuguu.....................................................................   22      1   Fm1
....................uacccuguagauccgaauuuguu.....................................................................   25056   0   Fm1
....................uacccuguagauccgaaGuuguu.....................................................................   120     1   Fm1
....................uacccuguagGuccgaauuuguu.....................................................................   27      1   Fm1
....................uacGcuguagauccgaauuuguu.....................................................................   2       1   Fm1
....................uacccuguagauccgaauuuguA.....................................................................   2017    1   Fm1
....................uacccuguagauccgaGuuuguu.....................................................................   45      1   Fm1
....................uacccuguGgauccgaauuuguu.....................................................................   1       1   Fm1
....................uacccuguagaucUgaauuuguu.....................................................................   90      1   Fm1
....................uaAccuguagauccgaauuuguu.....................................................................   7       1   Fm1
....................uacccuguagauccgaauGuguu.....................................................................   3       1   Fm1
....................uacccuguagauccgaauuugAu.....................................................................   3       1   Fm1
....................uacccuguagauccgaauuugCu.....................................................................   5       1   Fm1
....................uacccuguagaGccgaauuuguu.....................................................................   1       1   Fm1
....................uacccuguagaucAgaauuuguu.....................................................................   5       1   Fm1
....................uacccCguagauccgaauuuguu.....................................................................   8       1   Fm1
....................uaGccuguagauccgaauuuguu.....................................................................   6       1   Fm1
....................uacccuguagUuccgaauuuguu.....................................................................   27      1   Fm1
....................uaccUuguagauccgaauuuguu.....................................................................   3       1   Fm1
....................uacccuguagCuccgaauuuguu.....................................................................   28      1   Fm1
....................uacccuguagauccgaaCuuguu.....................................................................   160     1   Fm1
....................uacccuguagauccUaauuuguu.....................................................................   3       1   Fm1
....................uacccuguagauccgaauuugGu.....................................................................   1       1   Fm1
....................uacccuguagauccgaauuuguC.....................................................................   16      1   Fm1
....................uacccuguagauccgaaAuuguu.....................................................................   117     1   Fm1
....................uaccAuguagauccgaauuuguu.....................................................................   7       1   Fm1
....................uacccuguagauccgaaAuuguuu....................................................................   6       1   Fm1
....................uacccuguagauccgaauuuguuA....................................................................   202     1   Fm1
....................uaAccuguagauccgaauuuguuu....................................................................   1       1   Fm1
....................uacccuguagauccgaGuuuguuu....................................................................   3       1   Fm1
....................uacccuguagauccgaaGuuguuu....................................................................   4       1   Fm1
....................uacccuguagauccgaauuuguGu....................................................................   7       1   Fm1
....................uacccuguagauccgaauuuguuG....................................................................   3       1   Fm1
....................uacccuguagauccgaauuuguuu....................................................................   1289    0   Fm1
....................uacccuguagauccgaaCuuguuu....................................................................   9       1   Fm1
....................uacccuguagaCccgaauuuguuu....................................................................   1       1   Fm1
....................uacccuguUgauccgaauuuguuu....................................................................   2       1   Fm1
....................uacccuguagauccgaUuuuguuu....................................................................   2       1   Fm1
....................uacccuguagUuccgaauuuguuu....................................................................   2       1   Fm1
....................uacccuguagauccgaCuuuguuu....................................................................   5       1   Fm1
....................uacccuguagCuccgaauuuguuu....................................................................   1       1   Fm1
....................uacccAguagauccgaauuuguuu....................................................................   1       1   Fm1
....................uacccuguagGuccgaauuuguuu....................................................................   2       1   Fm1
....................uacccuguagauccgaauuAguuu....................................................................   1       1   Fm1
....................uacccuguagauccgaauuuguuC....................................................................   5       1   Fm1
....................uacccuguagaucUgaauuuguuu....................................................................   3       1   Fm1
....................uacccuguagauccgaauuuguAu....................................................................   1108    1   Fm1
....................uacccuguagauUcgaauuuguuu....................................................................   3       1   Fm1
....................uacccuguagauccgaauuuguCu....................................................................   24      1   Fm1
....................uacccuguagauccgaauuuAuuu....................................................................   2       1   Fm1
....................uacccuguagauccgaauuuguuug...................................................................   4       0   Fm1
....................uacccuguagauccgaauuuguuuU...................................................................   9       1   Fm1
....................uacccuguagauccgaauuuguuuA...................................................................   6       1   Fm1
....................uacccuguagauccgaauuuguuugu..................................................................   10      0   Fm1
....................uacccuguagauccgaauuuguuugugau...............................................................   9       0   Fm1
.....................acccuguagauccgaauu.........................................................................   19      0   Fm1
.....................acccuguagauccgaUuu.........................................................................   1       1   Fm1
.....................acccuguagauccgaauuu........................................................................   1       0   Fm1
.....................acccuguagauccgaauuug.......................................................................   8       0   Fm1
.....................acccuguagauccgaauGugu......................................................................   10      1   Fm1
.....................acccuguagauccgaauuuUu......................................................................   8       1   Fm1
.....................acccuguagauccgaauuugu......................................................................   3524    0   Fm1
.....................acccuguagaAccgaauuugu......................................................................   9       1   Fm1
.....................acccuguCgauccgaauuugu......................................................................   2       1   Fm1
.....................acccuguagauccgaaCuugu......................................................................   1       1   Fm1
.....................acccuguGgauccgaauuugu......................................................................   2       1   Fm1
.....................acccuguagauccgaauAugu......................................................................   12      1   Fm1
.....................acccuguagauGcgaauuugu......................................................................   1       1   Fm1
.....................acccuguagauccgaauuuCu......................................................................   2       1   Fm1
.....................Ucccuguagauccgaauuugu......................................................................   8       1   Fm1
```

Mature Star

agaguucgcccaucagcagauacccuguagauccgaauuuguuugugaucgauaugacaaauuugaauuugcagcgggaucugaaguuggguauaaucaacugacaggagga			
.....................acccuguUgauccgaauuugu..	1	1	Fm1
.....................acccCguagauccgaauuugu..	1	1	Fm1
.....................acccuguaCauccgaauuugu..	1	1	Fm1
.....................acccuguagauccgaGuuugu..	6	1	Fm1
.....................acccuguagauccgaauuGgu..	3	1	Fm1
.....................acccuguagauccgaUuuugu..	6	1	Fm1
.....................acccuguagaucAgaauuugu..	3	1	Fm1
.....................acccuguagaGccgaauuugu..	8	1	Fm1
.....................acccuUuagauccgaauuugu..	3	1	Fm1
.....................Ncccuguagauccgaauuugu..	1	1	Fm1
.....................acccuguagauccgaaAuugu..	5	1	Fm1
.....................acccuguagCuccgaauuugu..	1	1	Fm1
.....................acccuguagauccgaauuCgu..	1	1	Fm1
.....................acccuguagauccgaauuugA..	5	1	Fm1
.....................acccuguagaucGgaauuugu..	1	1	Fm1
.....................acccuguaAauccgaauuugu..	2	1	Fm1
.....................accAuguagauccgaauuugu..	8	1	Fm1
.....................acccuguagauccgaCuuugu..	7	1	Fm1
.....................acccuguagauccgaauCugu..	19	1	Fm1
.....................aAccuguagauccgaauuugu..	2	1	Fm1
.....................acccuguagauccgaauuAgu..	7	1	Fm1
.....................acccuguagauccgaauuugG..	16	1	Fm1
.....................acccuguagauccgaauuugC..	2	1	Fm1
.....................acccuAuagauccgaauuugu..	2	1	Fm1
.....................acccuguagauccgGauuugu..	2	1	Fm1
.....................acccuguagaCccgaauuugu..	7	1	Fm1
.....................accUuguagauccgaauuugu..	1	1	Fm1
.....................acccuguagUuccgaauuugu..	3	1	Fm1
.....................acGcuguagauccgaauuugu..	1	1	Fm1
.....................acNcuguagauccgaauuugu..	1	1	Fm1
.....................acccuguagauccgaauuuAu..	2	1	Fm1
.....................acAcuguagauccgaauuugu..	4	1	Fm1
.....................acccuguagauccgaaGuugu..	4	1	Fm1
.....................acccuguagauUcgaauuugu..	2	1	Fm1
.....................acccuguagauccCaauuugu..	1	1	Fm1
.....................acccuguagaucUgaauuugu..	13	1	Fm1
.....................acccuguagauccgUauuugu..	4	1	Fm1
.....................acccuguagauccgCauuugu..	3	1	Fm1
.....................acccuguaUauccgaauuugu..	1	1	Fm1
.....................accGuguagauccgaauuugu..	2	1	Fm1
.....................acccuguagauccAaauuugu..	4	1	Fm1
.....................acGcuguagauccgaauuuguu...	1	1	Fm1
.....................acccuguagaGccgaauuuguu...	2	1	Fm1
.....................acccuguagauccgaauuuguA...	2	1	Fm1
.....................acccuguagauccgaauuuguu...	234	0	Fm1
.....................acccuguagauccgaaAuuguu...	2	1	Fm1
.....................acccuguagauccgaauuuguuA..	8	1	Fm1
.....................acccuguagauccgaauuuguAu..	7	1	Fm1
.....................acccuguagauccgaauuuguuu..	3	0	Fm1
......................cccuguagauccgaauuCgu..	1	1	Fm1
......................cccuguagauccgaauuugu..	169	0	Fm1
......................cccuguagauccgaUuuugu..	1	1	Fm1
......................cccuguGgauccgaauuugu..	1	1	Fm1
......................cccuguaAauccgaauuugu..	2	1	Fm1
......................cccuguagauccgaauuGgu..	1	1	Fm1
......................cccuguagUuccgaauuugu..	1	1	Fm1
......................cccugAagauccgaauuugu..	1	1	Fm1
......................cccuguagauccgaauCugu..	1	1	Fm1
......................cccuguagauccgaauuuguu...	3	0	Fm1
.......................ccuguagauccgaauuugu..	57	0	Fm1
.......................ccuguagaucAgaauuugu..	1	1	Fm1
........................cuguagauccgaauuugu..	5	0	Fm1
........................cuguagauccgaaCuugu..	1	1	Fm1
....uguuugugaucgauaugacaaauuugaauuugcagcgggauc...............................	3	0	Fm1
....guuugugaucgauaugacaaauuugaauuugcagcgggauc...............................	1	0	Fm1
....caaauuugaauuugcagcgggau................................	6	0	Fm1
....caaauuugaauuugcagUgggau................................	1	1	Fm1
....caaauuugaauuugcagcgggauU...............................	2	1	Fm1
....caaauuugaauuugcagcgggauc...............................	1	0	Fm1
....caaauuugaauuugcagcgggauA...............................	2	1	Fm1

Mature Star

```
agaguucgcccaucagcagauacccuguagauccgaauuuguuugugaucgauaugacaaauuugaauuugcagcgggaucugaaguugggguauaaucaacugacaggagga
..........................................................aaauuugaauuugcagcgggau.................................   7     0    Fm1
..........................................................aaauuugaauuugcagcUggauc................................   1     1    Fm1
..........................................................aaauuugaauuugcagcgggauc................................   89    0    Fm1
..........................................................aaauuugaauuugcagAgggauc................................   1     1    Fm1
..........................................................aaauuugaauuugcagcgggaucu...............................   8     0    Fm1
...........................................................aauuugaauuugcagcgggauc................................   7     0    Fm1
..............................................................uugaauuugcagcgggauc................................   3     0    Fm1

.gaguucgcccaucagcaga.............................................................................................   1     0    Mm2
..................gGuacccuguagauccgaauuugu.......................................................................   1     1    Mm2
...................auacccuguagaAccgaa............................................................................   9     1    Mm2
...................auacccuguagaAccgaau...........................................................................   20    1    Mm2
...................Cuacccuguagauccgaau...........................................................................   1     1    Mm2
...................auacccuguagauccgaau...........................................................................   2     0    Mm2
...................auacccuguagaAccgaauu..........................................................................   4     1    Mm2
...................Cuacccuguagauccgaauuu.........................................................................   2     1    Mm2
...................Cuacccuguagauccgaauuug........................................................................   3     1    Mm2
...................auacccuguagauccgaauuug........................................................................   2     0    Mm2
...................auacccuguagauccgaGuuugu.......................................................................   1     1    Mm2
...................aAacccuguagauccgaauuugu.......................................................................   1     1    Mm2
...................auacccuguagauccgaaCuugu.......................................................................   3     1    Mm2
...................auacccuguaAauccgaauuugu.......................................................................   1     1    Mm2
...................auacccuguagauccgaauuugG.......................................................................   2     1    Mm2
...................auacccuguagauccgaCuuugu.......................................................................   1     1    Mm2
...................Guacccuguagauccgaauuugu.......................................................................   3     1    Mm2
...................Cuacccuguagauccgaauuugu.......................................................................   409   1    Mm2
...................auacccuguagaAccgaauuugu.......................................................................   32    1    Mm2
...................auacccuguagauccgaauuugu.......................................................................   269   0    Mm2
...................Cuacccuguagauccgaauuuguu......................................................................   3     1    Mm2
...................auacccuguagauccgaauuuguu......................................................................   1     0    Mm2
....................uacccuguagGuccgaau...........................................................................   1     1    Mm2
....................uUcccuguagauccgaau...........................................................................   1     1    Mm2
....................uacccuguagauccgaau...........................................................................   1712  0    Mm2
....................uacccuguagUuccgaau...........................................................................   5     1    Mm2
....................uacccuAuagauccgaau...........................................................................   1     1    Mm2
....................uacccuguagauccgUau...........................................................................   3     1    Mm2
....................uaUccuguagauccgaau...........................................................................   1     1    Mm2
....................uacccuguagaucUgaau...........................................................................   1     1    Mm2
....................uacccuguagaucAgaau...........................................................................   2     1    Mm2
....................uacccuguagauccgaaC...........................................................................   17    1    Mm2
....................uacccuguagauUcgaau...........................................................................   2     1    Mm2
....................uacccuUuagauccgaau...........................................................................   1     1    Mm2
....................uacccuguagauccgaaG...........................................................................   15    1    Mm2
....................uacccAguagauccgaau...........................................................................   1     1    Mm2
....................uacccuguagauccgaaA...........................................................................   47    1    Mm2
....................uacccuguagauccgaCu...........................................................................   5     1    Mm2
....................uacccuguagaAccgaau...........................................................................   5     1    Mm2
....................uacccuguagauccgCau...........................................................................   1     1    Mm2
....................uacccCguagauccgaau...........................................................................   1     1    Mm2
....................uacAcuguagauccgaau...........................................................................   2     1    Mm2
....................uacccuguagauccgaUu...........................................................................   2     1    Mm2
....................uacccuguagauccgaGu...........................................................................   3     1    Mm2
....................uacccuguagCuccgaau...........................................................................   1     1    Mm2
....................uacccuguUgauccgaau...........................................................................   1     1    Mm2
....................Nacccuguagauccgaau...........................................................................   1     1    Mm2
....................uaccUuguagauccgaauu..........................................................................   1     1    Mm2
....................uCcccuguagauccgaauu..........................................................................   1     1    Mm2
....................uaUccuguagauccgaauu..........................................................................   1     1    Mm2
....................Aacccuguagauccgaauu..........................................................................   1     1    Mm2
....................uacccuguagauccgaGuu..........................................................................   5     1    Mm2
....................uacccuguagUuccgaauu..........................................................................   1     1    Mm2
....................uacccuguagauccgaCuu..........................................................................   12    1    Mm2
....................uacccuguaUauccgaauu..........................................................................   1     1    Mm2
....................uacccGguagauccgaauu..........................................................................   1     1    Mm2
....................uacccuguagauccgaauA..........................................................................   143   1    Mm2
....................uaAccuguagauccgaauu..........................................................................   1     1    Mm2
....................uacccNguagauccgaauu..........................................................................   2     1    Mm2
....................uGcccuguagauccgaauu..........................................................................   1     1    Mm2
....................uacccuguagauccgaUuu..........................................................................   7     1    Mm2
....................uacccuguagCuccgaauu..........................................................................   8     1    Mm2
```

Mature **Star**

agaguucgcccaucagcagauacccuguagauccgaauuuguuugugaucgauaugacaaauuugaauuugcagcgggaucugaaguuggguauaaucaacugacaggagga			
....................Nacccuguagauccgaauu...	1	1	Mm2
....................uacccuguUgauccgaauu...	1	1	Mm2
....................uacccuguagauccgaaGu...	21	1	Mm2
....................uacccuguagaAccgaauu...	2	1	Mm2
....................uacccuguagauccgaaAu...	36	1	Mm2
....................uacccuguagauccgaauu...	3779	0	Mm2
....................uacccuguagauccgUauu...	1	1	Mm2
....................uacGcuguagauccgaauu...	1	1	Mm2
....................uacccuguagauccgaauG...	1	1	Mm2
....................uacccuAuagauccgaauu...	1	1	Mm2
....................uacccuguagaucUgaauu...	1	1	Mm2
....................uUcccuguagauccgaauu...	1	1	Mm2
....................uacccuguagaucAgaauu...	1	1	Mm2
....................uacccCguagauccgaauu...	2	1	Mm2
....................uacccuguaCauccgaauu...	1	1	Mm2
....................uacccuguagauccgaaCu...	29	1	Mm2
....................uacccuguagGuccgaauu...	3	1	Mm2
....................uacccuguagauUcgaauu...	5	1	Mm2
....................uacccuguagauccgaauuG..	1	1	Mm2
....................uaccAuguagauccgaauuu..	1	1	Mm2
....................uGcccuguagauccgaauuu..	2	1	Mm2
....................uacccuguagauccgaauCu..	1	1	Mm2
....................uacccuguagauccgaauuA..	17	1	Mm2
....................uacccuguagUuccgaauuu..	3	1	Mm2
....................uacccuguagauccgGauuu..	2	1	Mm2
....................uacccNguagauccgaauuu..	1	1	Mm2
....................uacAcuguagauccgaauuu..	2	1	Mm2
....................uacccuguagauccgaaCuu..	28	1	Mm2
....................Cacccuguagauccgaauuu..	1	1	Mm2
....................uacccuguagauccgaauGu..	1	1	Mm2
....................uacccuguagauccgaUuuu..	2	1	Mm2
....................uaGccuguagauccgaauuu..	3	1	Mm2
....................uacccuguagauccUaauuu..	1	1	Mm2
....................uacccuguagauccgaGuuu..	6	1	Mm2
....................uUcccuguagauccgaauuu..	3	1	Mm2
....................uacccuguagauccgaauuC..	1	1	Mm2
....................Aacccuguagauccgaauuu..	1	1	Mm2
....................uaUccuguagauccgaauuu..	2	1	Mm2
....................uacccuguagauUcgaauuu..	3	1	Mm2
....................uacccugCagauccgaauuu..	1	1	Mm2
....................uacccuguagauccgaCuuu..	15	1	Mm2
....................uacccuguagauccgaauAu..	10	1	Mm2
....................uacccuguagCuccgaauuu..	4	1	Mm2
....................uacccuNuagauccgaauuu..	1	1	Mm2
....................uacccAguagauccgaauuu..	1	1	Mm2
....................uacccuguagGuccgaauuu..	4	1	Mm2
....................uacccuguagauccgaauuu..	4071	0	Mm2
....................Nacccuguagauccgaauuu..	2	1	Mm2
....................uacUcuguagauccgaauuu..	1	1	Mm2
....................uacccuguagauccgaaGuu..	22	1	Mm2
....................uacccuguagauccgaaAuu..	19	1	Mm2
....................uacccuguagaucUgaauuu..	7	1	Mm2
....................uacGcuguagauccgaauuu..	1	1	Mm2
....................uCcccuguagauccgaauuu..	1	1	Mm2
....................uaccAuguagauccgaauuug...	3	1	Mm2
....................uacccuguagauccgaaGuug...	50	1	Mm2
....................uacccuguagauccgaUuuug...	18	1	Mm2
....................Nacccuguagauccgaauuug...	7	1	Mm2
....................uUcccuguagauccgaauuug...	1	1	Mm2
....................uacUcuguagauccgaauuug...	4	1	Mm2
....................uacccuguagauccgUauuug...	1	1	Mm2
....................uaAccuguagauccgaauuug...	2	1	Mm2
....................uaUccuguagauccgaauuug...	1	1	Mm2
....................uGcccuguagauccgaauuug...	2	1	Mm2
....................uacccAguagauccgaauuug...	2	1	Mm2
....................uacccuguagauccUaauuug...	2	1	Mm2
....................uacccuguagauccgaauuuA...	83	1	Mm2
....................uaGccuguagauccgaauuug...	8	1	Mm2
....................uacccuguagauccgaaAuug...	45	1	Mm2
....................uacccuguagauccgaCuuug...	21	1	Mm2

Mature   Star

```
agaguucgcccaucagcagauacccuguagauccgaauuuguuugugaucgauaugacaaauuugaauuugcagcgggaucugaaguuggguauaaucaacugacaggagga
....................uacccuguagauUcgaauuug.......................................................................  9      1  Mm2
....................uacccuguGgauccgaauuug.......................................................................  3      1  Mm2
....................uacccuguagauccgaauuuC.......................................................................  1      1  Mm2
....................uacccuguagauAcgaauuug.......................................................................  1      1  Mm2
....................uacccuguagUuccgaauuug.......................................................................  15     1  Mm2
....................uacccugCagauccgaauuug.......................................................................  1      1  Mm2
....................uacccuguagCuccgaauuug.......................................................................  23     1  Mm2
....................uaccUuguagauccgaauuug.......................................................................  6      1  Mm2
....................uacccuguagaucAgaauuug.......................................................................  2      1  Mm2
....................uacGcuguagauccgaauuug.......................................................................  1      1  Mm2
....................uacccuCuagauccgaauuug.......................................................................  1      1  Mm2
....................uacccuguagGuccgaauuug.......................................................................  21     1  Mm2
....................uacccuguagauccgaGuuug.......................................................................  18     1  Mm2
....................uacccuguagauccAaauuug.......................................................................  3      1  Mm2
....................uacccugGagauccgaauuug.......................................................................  1      1  Mm2
....................uacAcuguagauccgaauuug.......................................................................  2      1  Mm2
....................uacccuguagauccgaauuAg.......................................................................  1      1  Mm2
....................uacccuguagaucGgaauuug.......................................................................  1      1  Mm2
....................uacccuguagauccgaauuuU.......................................................................  50     1  Mm2
....................uacccuguagauccgaauuCg.......................................................................  1      1  Mm2
....................uacccuguagaAccgaauuug.......................................................................  1      1  Mm2
....................uacccuguagauccgaaCuug.......................................................................  100    1  Mm2
....................Cacccuguagauccgaauuug.......................................................................  2      1  Mm2
....................Aacccuguagauccgaauuug.......................................................................  1      1  Mm2
....................uacccuguagauccgaauCug.......................................................................  1      1  Mm2
....................uacccuguagauccgaauAug.......................................................................  1      1  Mm2
....................uacccugAagauccgaauuug.......................................................................  1      1  Mm2
....................uacccuguagauccgCauuug.......................................................................  1      1  Mm2
....................uacccuguagauccgaauuug.......................................................................  12118  0  Mm2
....................uacccuguagaucUgaauuug.......................................................................  9      1  Mm2
....................uacccuguaAauccgaauuug.......................................................................  2      1  Mm2
....................uacccCguagauccgaauuug.......................................................................  2      1  Mm2
....................uacccuguagauccgaauuGg.......................................................................  1      1  Mm2
....................uGcccuguagauccgaauuugu......................................................................  411    1  Mm2
....................uacccuNuagauccgaauuugu......................................................................  92     1  Mm2
....................uacccuguagauccgaauGugu......................................................................  56     1  Mm2
....................uacccuguaAauccgaauuugu......................................................................  101    1  Mm2
....................uacccuguagauccgaNuuugu......................................................................  1      1  Mm2
....................uaGccuguagauccgaauuugu......................................................................  400    1  Mm2
....................uacccuguagauccgUauuugu......................................................................  382    1  Mm2
....................uacccuguagauccgaaCuugu......................................................................  9863   1  Mm2
....................uacccuguagauccUaauuugu......................................................................  124    1  Mm2
....................uacccuguagUuccgaauuugu......................................................................  1251   1  Mm2
....................uacccuguaUauccgaauuugu......................................................................  124    1  Mm2
....................uacccCguagauccgaauuugu......................................................................  178    1  Mm2
....................uCcccuguagauccgaauuugu......................................................................  112    1  Mm2
....................uacccuguagauccgaGuuugu......................................................................  2885   1  Mm2
....................uacccugGagauccgaauuugu......................................................................  47     1  Mm2
....................uacccuguaCauccgaauuugu......................................................................  40     1  Mm2
....................uacccuguagaucGgaauuugu......................................................................  89     1  Mm2
....................uacccuguagauccgaauNugu......................................................................  1      1  Mm2
....................uacccuguagauccgaauuugG......................................................................  3431   1  Mm2
....................uacccuguagauUcgaauuugu......................................................................  554    1  Mm2
....................uaccGuguagauccgaauuugu......................................................................  77     1  Mm2
....................uacccuguagaucUgaauuugu......................................................................  839    1  Mm2
....................uacccuUuagauccgaauuugu......................................................................  140    1  Mm2
....................uacccuguagaucAgaauuugu......................................................................  364    1  Mm2
....................uacGcuguagauccgaauuugu......................................................................  132    1  Mm2
....................uacccuguagauccgaauuAgu......................................................................  92     1  Mm2
....................uacccuguagauccgaauuugA......................................................................  938    1  Mm2
....................uacccuguagaAccgaauuugu......................................................................  172    1  Mm2
....................uacccNguagauccgaauuugu......................................................................  221    1  Mm2
....................uacccuguagauccgaauuuCu......................................................................  54     1  Mm2
....................uacccuguagauAcgaauuugu......................................................................  69     1  Mm2
....................uacccugCagauccgaauuugu......................................................................  141    1  Mm2
....................uacccuguagauccgCauuugu......................................................................  118    1  Mm2
....................uaNccuguagauccgaauuugu......................................................................  9      1  Mm2
....................uacccuCuagauccgaauuugu......................................................................  55     1  Mm2
....................uacccugAagauccgaauuugu......................................................................  124    1  Mm2
....................uacAcuguagauccgaauuugu......................................................................  587    1  Mm2
```

```
                    Mature                                Star
agaguucgcccaucagcagauacccuguagauccgaauuuguuugugaucgauaugacaaauuugaauuugcagcgggaucugaaguuggguauaaucaacugacaggagga
....................uacccuguagauccgaaAuugu......................................................................   7234     1   Mm2
....................uacccuguagauccgaaGuugu......................................................................   7970     1   Mm2
....................uacccuguagauccgaCuuugu......................................................................   3860     1   Mm2
....................uacccuguagaGccgaauuugu......................................................................   31       1   Mm2
....................uacccuguagauccgaUuuugu......................................................................   2513     1   Mm2
....................uacccuguagauccgaauAugu......................................................................   75       1   Mm2
....................uacccuguagauccCaauuugu......................................................................   42       1   Mm2
....................uacccuguagauccgaauuugC......................................................................   691      1   Mm2
....................Nacccuguagauccgaauuugu......................................................................   741      1   Mm2
....................uacccuguagauccgaauCugu......................................................................   188      1   Mm2
....................uacccuguagauccgaauuugu......................................................................   1537579  0   Mm2
....................uacccuguagauccgaauuuUu......................................................................   143      1   Mm2
....................Gacccuguagauccgaauuugu......................................................................   25       1   Mm2
....................uacccuguagauccgGauuugu......................................................................   222      1   Mm2
....................uacccuguagGuccgaauuugu......................................................................   1648     1   Mm2
....................uacccuAuagauccgaauuugu......................................................................   160      1   Mm2
....................uacccuguCgauccgaauuugu......................................................................   25       1   Mm2
....................uaUccuguagauccgaauuugu......................................................................   322      1   Mm2
....................Aacccuguagauccgaauuugu......................................................................   324      1   Mm2
....................uacccuguGgauccgaauuugu......................................................................   170      1   Mm2
....................uacccuguagCuccgaauuugu......................................................................   1475     1   Mm2
....................uacccuguagaCccgaauuugu......................................................................   173      1   Mm2
....................uacUcuguagauccgaauuugu......................................................................   331      1   Mm2
....................uacccAguagauccgaauuugu......................................................................   81       1   Mm2
....................uacccuguagauccgaauuuAu......................................................................   113      1   Mm2
....................uUccuguagauccgaauuugu......................................................................   429      1   Mm2
....................uacccuguagauccgaauuCgu......................................................................   150      1   Mm2
....................uaccAuguagauccgaauuugu......................................................................   158      1   Mm2
....................uacccuguagauccAaauuugu......................................................................   185      1   Mm2
....................uacccGguagauccgaauuugu......................................................................   84       1   Mm2
....................uacccuguUgauccgaauuugu......................................................................   152      1   Mm2
....................uaAccuguagauccgaauuugu......................................................................   358      1   Mm2
....................uacccuguagauccgaaNuugu......................................................................   2        1   Mm2
....................uacccuguagauGcgaauuugu......................................................................   33       1   Mm2
....................uNccuguagauccgaauuugu......................................................................   7        1   Mm2
....................uaccUuguagauccgaauuugu......................................................................   270      1   Mm2
....................Cacccuguagauccgaauuugu......................................................................   274      1   Mm2
....................uacccuguagauccgaauuGgu......................................................................   53       1   Mm2
....................uacccuUuagauccgaauuuguu....................................................................   6        1   Mm2
....................uacccNguagauccgaauuuguu....................................................................   5        1   Mm2
....................uacccuguUgauccgaauuuguu....................................................................   2        1   Mm2
....................uacccugAagauccgaauuuguu....................................................................   1        1   Mm2
....................uUccuguagauccgaauuuguu....................................................................   9        1   Mm2
....................uacccuguagaCccgaauuuguu....................................................................   6        1   Mm2
....................uacccuguagauccgaauAuguu....................................................................   2        1   Mm2
....................uacccuguagauccgaauuugAu....................................................................   6        1   Mm2
....................uacccuguagauccgaaGuuguu....................................................................   104      1   Mm2
....................uGcccuguagauccgaauuuguu....................................................................   13       1   Mm2
....................uacccCguagauccgaauuuguu....................................................................   1        1   Mm2
....................uacccuCuagauccgaauuuguu....................................................................   1        1   Mm2
....................uacAcuguagauccgaauuuguu....................................................................   5        1   Mm2
....................uacccuguagauccgaaAuuguu....................................................................   116      1   Mm2
....................Nacccuguagauccgaauuuguu....................................................................   10       1   Mm2
....................uacccuguagauccgaGuuuguu....................................................................   41       1   Mm2
....................uacccuguagaAccgaauuuguu....................................................................   1        1   Mm2
....................uacccuguagauccgaCuuuguu....................................................................   49       1   Mm2
....................uacccuguagauccgaauuuguC....................................................................   13       1   Mm2
....................uacccuguagauccgaauuAguu....................................................................   1        1   Mm2
....................uacccuNuagauccgaauuuguu....................................................................   3        1   Mm2
....................uacccuguagauccgaauCuguu....................................................................   1        1   Mm2
....................uacccuguagauccgaauuuAuu....................................................................   1        1   Mm2
....................uacccuguagaucUgaauuuguu....................................................................   35       1   Mm2
....................uacccuguagauccUaauuuguu....................................................................   1        1   Mm2
....................uacccuguagUuccgaauuuguu....................................................................   14       1   Mm2
....................uaccGuguagauccgaauuuguu....................................................................   1        1   Mm2
....................uacUcuguagauccgaauuuguu....................................................................   1        1   Mm2
....................uacccuguagGuccgaauuuguu....................................................................   27       1   Mm2
....................uacccuguagauUcgaauuuguu....................................................................   5        1   Mm2
....................uacccGguagauccgaauuuguu....................................................................   1        1   Mm2
....................uacccuguagauccgaUuuuguu....................................................................   47       1   Mm2
```

Mature **Star**

```
agaguucgcccaucagcagauacccuguagauccgaauuuguuugugaucgauaugacaaauuugaauuugcagcgggaucugaaguuggguauaaucaacugacaggagga
....................uacccuguGgauccgaauuuguu.....................................................................   1      1   Mm2
....................uacccuguaUauccgaauuuguu.....................................................................   3      1   Mm2
....................uacccuguagauccgGauuuguu.....................................................................   4      1   Mm2
....................uaccUuguagauccgaauuuguu.....................................................................   3      1   Mm2
....................uacccuguagCuccgaauuuguu.....................................................................   29     1   Mm2
....................uaUccuguagauccgaauuuguu.....................................................................   5      1   Mm2
....................uaccAuguagauccgaauuuguu.....................................................................   1      1   Mm2
....................uacccuguagauccgaauuuguu.....................................................................   22554  0   Mm2
....................uacccuguagauccgaauuugCu.....................................................................   4      1   Mm2
....................uacccuguagauccgUauuuguu.....................................................................   3      1   Mm2
....................uCcccuguagauccgaauuuguu.....................................................................   2      1   Mm2
....................uacccAguagauccgaauuuguu.....................................................................   4      1   Mm2
....................uacccuguagauccgaauGuguu.....................................................................   1      1   Mm2
....................uacccuguagauccgaaCuuguu.....................................................................   145    1   Mm2
....................uaGccuguagauccgaauuuguu.....................................................................   2      1   Mm2
....................uacccuguagauccgaauuuguA.....................................................................   4047   1   Mm2
....................uacccuguagauccAaauuuguu.....................................................................   1      1   Mm2
....................uacccuguagauccgaauuCguu.....................................................................   3      1   Mm2
....................uacccuguagauccgaauuuUuu.....................................................................   3      1   Mm2
....................uacccuguagauAcgaauuuguu.....................................................................   1      1   Mm2
....................uacccugCagauccgaauuuguu.....................................................................   1      1   Mm2
....................uaAccuguagauccgaauuuguu.....................................................................   5      1   Mm2
....................Cacccuguagauccgaauuuguu.....................................................................   2      1   Mm2
....................Aacccuguagauccgaauuuguu.....................................................................   7      1   Mm2
....................uacccuguagaucAgaauuuguu.....................................................................   4      1   Mm2
....................uacccuguaAauccgaauuuguu.....................................................................   2      1   Mm2
....................uacccuguagauccgaauuuguG.....................................................................   20     1   Mm2
....................uaNccuguagauccgaauuuguu.....................................................................   2      1   Mm2
....................uaGccuguagauccgaauuuguuu....................................................................   1      1   Mm2
....................uacccuguagauccgaauuuguuG....................................................................   3      1   Mm2
....................uacccuguagauccgaauuuguAu....................................................................   916    1   Mm2
....................Nacccuguagauccgaauuuguuu....................................................................   2      1   Mm2
....................uacccuguagauccgaauuuguGu....................................................................   5      1   Mm2
....................uacccuguagauccgaCuuuguuu....................................................................   2      1   Mm2
....................uacccuguagauccgaaAuuguuu....................................................................   5      1   Mm2
....................uacccuguagauccgaGuuuguuu....................................................................   2      1   Mm2
....................uacccuguagaucUgaauuuguuu....................................................................   2      1   Mm2
....................uacccuguagauccgaauuuguCu....................................................................   15     1   Mm2
....................uacccuguagUuccgaauuuguuu....................................................................   1      1   Mm2
....................uacccuguagauccgaaCuuguuu....................................................................   3      1   Mm2
....................uacccuguagauccgaauuuguuC....................................................................   2      1   Mm2
....................uacccuguagGuccgaauuuguuu....................................................................   1      1   Mm2
....................uacccuguagauccgaauuuguuA....................................................................   280    1   Mm2
....................uacccuguagauccgaauuuguuu....................................................................   615    0   Mm2
....................uacccuguagauccgaaGuuguuu....................................................................   1      1   Mm2
....................uacccuguagauccAaauuuguuu....................................................................   1      1   Mm2
....................uacccuguagauccgaauuuguuuU...................................................................   3      1   Mm2
....................uacccuguagauccgaauuuguuug...................................................................   4      0   Mm2
....................uacccuguagauccgaauuuguuuA...................................................................   1      1   Mm2
....................uacccuguagauccgaauuuguuugu..................................................................   6      0   Mm2
....................uacccuguagauccgaauuuguuugug.................................................................   1      0   Mm2
....................uacccuguagCuccgaauuuguuugugau...............................................................   1      1   Mm2
....................uacccuguagauccgaauuuguuugugau...............................................................   12     0   Mm2
....................uacccuguagauccgaauuuguuugugaucgauaugac......................................................   1      0   Mm2
.....................acccuguagauccgaauC.........................................................................   1      1   Mm2
.....................acccuguagauccgaauu.........................................................................   14     0   Mm2
.....................acccuguagauccgaauuu........................................................................   17     0   Mm2
.....................acccuUuagauccgaauuu........................................................................   1      1   Mm2
.....................acccuCuagauccgaauuug.......................................................................   1      1   Mm2
.....................Ucccuguagauccgaauuug.......................................................................   1      1   Mm2
.....................acccuguagauccgaauuuU.......................................................................   1      1   Mm2
.....................acccuguagauccgaauuCg.......................................................................   1      1   Mm2
.....................acccuguagauccgaauuuA.......................................................................   1      1   Mm2
.....................acccuguagauccgaauuug.......................................................................   27     0   Mm2
.....................acccuguagauccgaauuCgu......................................................................   10     1   Mm2
.....................acccuguagaucUgaauuugu......................................................................   5      1   Mm2
.....................acccuguagauccgaauGugu......................................................................   19     1   Mm2
.....................acccuNuagauccgaauuugu......................................................................   2      1   Mm2
.....................acccuguagauccCaauuugu......................................................................   3      1   Mm2
.....................acccuguagauAcgaauuugu......................................................................   2      1   Mm2
```

Mature Star

```
agaguucgcccaucagcagauacccuguagauccgaauuuguuugugaucgauaugacaaauuugaauuugcagcgggaucugaaguuggguauaaucaacugacaggagga
.....................acGcuguagauccgaauuugu......................................................................   4     1   Mm2
.....................acccuguagauccgaauuuUu......................................................................   8     1   Mm2
.....................acccuguagauccgaGuuugu......................................................................   8     1   Mm2
.....................acccuguagaucAgaauuugu......................................................................   7     1   Mm2
.....................acccuguagauccgaaAuugu......................................................................   4     1   Mm2
.....................acccuguagGuccgaauuugu......................................................................   2     1   Mm2
.....................acccuguagauccgaauuugu......................................................................   6992  0   Mm2
.....................acccuguagauccgGauuugu......................................................................   2     1   Mm2
.....................acUcuguagauccgaauuugu......................................................................   4     1   Mm2
.....................acccuguagauccgaCuuugu......................................................................   16    1   Mm2
.....................acccuCuagauccgaauuugu......................................................................   1     1   Mm2
.....................acccuguagauccgaauuugC......................................................................   4     1   Mm2
.....................acccuguagauccAaauuugu......................................................................   2     1   Mm2
.....................acccuguagauccgaaGuugu......................................................................   7     1   Mm2
.....................acccuguagauccgaauuGgu......................................................................   5     1   Mm2
.....................acccuguagaAccgaauuugu......................................................................   17    1   Mm2
.....................acccuguaAauccgaauuugu......................................................................   8     1   Mm2
.....................acccuguagauccgaauuugA......................................................................   5     1   Mm2
.....................acccuguagaGccgaauuugu......................................................................   7     1   Mm2
.....................acccuguagauccgaauuugG......................................................................   24    1   Mm2
.....................acccugGagauccgaauuugu......................................................................   2     1   Mm2
.....................acccuguagauccgaauuuAu......................................................................   3     1   Mm2
.....................acccuguagauccgaauAugu......................................................................   22    1   Mm2
.....................acccuguagCuccgaauuugu......................................................................   7     1   Mm2
.....................accAuguagauccgaauuugu......................................................................   3     1   Mm2
.....................acccuguagauccgaaCuugu......................................................................   4     1   Mm2
.....................acAcuguagauccgaauuugu......................................................................   3     1   Mm2
.....................acccuguagauccgUauuugu......................................................................   7     1   Mm2
.....................accUuguagauccgaauuugu......................................................................   1     1   Mm2
.....................acccuguagaCccgaauuugu......................................................................   11    1   Mm2
.....................acccuguagauccgaauuuCu......................................................................   5     1   Mm2
.....................acccuguagauccgaauCugu......................................................................   47    1   Mm2
.....................acccuguagauccgaauuAgu......................................................................   8     1   Mm2
.....................Ncccuguagauccgaauuugu......................................................................   4     1   Mm2
.....................Ucccuguagauccgaauuugu......................................................................   7     1   Mm2
.....................aGccuguagauccgaauuugu......................................................................   2     1   Mm2
.....................acccuguaUauccgaauuugu......................................................................   6     1   Mm2
.....................accGuguagauccgaauuugu......................................................................   1     1   Mm2
.....................aUccuguagauccgaauuugu......................................................................   3     1   Mm2
.....................acccugCagauccgaauuugu......................................................................   4     1   Mm2
.....................acccCguagauccgaauuugu......................................................................   1     1   Mm2
.....................acccuAuagauccgaauuugu......................................................................   6     1   Mm2
.....................acccuguagauccgaUuuugu......................................................................   2     1   Mm2
.....................aAccuguagauccgaauuugu......................................................................   4     1   Mm2
.....................acccuguagauccgCauuugu......................................................................   11    1   Mm2
.....................acccuguagUuccgaauuugu......................................................................   3     1   Mm2
.....................acccuUuagauccgaauuugu......................................................................   2     1   Mm2
.....................acccuguaCauccgaauuugu......................................................................   9     1   Mm2
.....................acccuguagauccUaauuugu......................................................................   1     1   Mm2
.....................acccuguagauUcgaauuugu......................................................................   11    1   Mm2
.....................Ccccuguagauccgaauuugu......................................................................   1     1   Mm2
.....................acccuguagaucGgaauuugu......................................................................   2     1   Mm2
.....................acccuguagauccgaaAuuguu.....................................................................   1     1   Mm2
.....................acccuguaUauccgaauuuguu.....................................................................   1     1   Mm2
.....................acccuguagauccgaauuuguu.....................................................................   160   0   Mm2
.....................acccuguagauccgaauuuguG.....................................................................   1     1   Mm2
.....................acccuguagauccgaauCuguu.....................................................................   1     1   Mm2
.....................acccuguagaAccgaauuuguu.....................................................................   1     1   Mm2
.....................acccuguagaucUgaauuuguu.....................................................................   3     1   Mm2
.....................acccuguagauccgaauuuguA.....................................................................   19    1   Mm2
.....................acccuguagaGccgaauuuguu.....................................................................   1     1   Mm2
.....................acccuguagauccgaauuuguuA....................................................................   3     1   Mm2
.....................acccuguagauccgaauuuguGu....................................................................   1     1   Mm2
.....................acccuguagauccgaauuuguuu....................................................................   3     0   Mm2
......................cccuUuagauccgaauuugu......................................................................   1     1   Mm2
......................cccuguagauccgaauCugu......................................................................   2     1   Mm2
......................cccuguagauccgaauuugG......................................................................   1     1   Mm2
......................cccuguagauccgaauuuUu......................................................................   6     1   Mm2
......................cccuguagauccgaauuugu......................................................................   265   0   Mm2
......................cccuguagauccgaauuGgu......................................................................   3     1   Mm2
```

```
                    Mature                               Star
agaguucgcccaucagcagauacccuguagauccgaauuuguuugugaucgauaugacaaauuugaauuugcagcgggaucugaaguuggguauaaucaacugacaggagga
......................cccuguagauccgaauuAgu......................................................................    3      1     Mm2
......................cccuguagauccgaauuCgu......................................................................    1      1     Mm2
......................cccuguGgauccgaauuugu......................................................................    2      1     Mm2
......................cccuguagUuccgaauuugu......................................................................    5      1     Mm2
......................cccuguaAauccgaauuugu......................................................................    2      1     Mm2
......................cccugAagauccgaauuugu......................................................................    1      1     Mm2
......................cccuguagauUcgaauuugu......................................................................    1      1     Mm2
......................cccuguagauccgaauuuguu.....................................................................    6      0     Mm2
.......................ccuguagauccgUauuugu......................................................................    1      1     Mm2
.......................ccuguagauccgaauuuCu......................................................................    1      1     Mm2
.......................ccuguagauccgaauuugu......................................................................    41     0     Mm2
........................cuguagauccgaauuugu......................................................................    6      0     Mm2
.........................uguagauccgaauuuguu.....................................................................    1      0     Mm2
..........................guagauccgaaCuuguuu....................................................................    1      1     Mm2
.........................................uuugugaucgauaugacG.....................................................    1      1     Mm2
.........................................................caaauuugaauuugcagcgggau................................    2      0     Mm2
.........................................................caaauuugaauuugcagcgggauc...............................    1      0     Mm2
.........................................................caaauuugaauuugcagcgggauU...............................    2      1     Mm2
..........................................................aaauuugaauuugcagcgggau................................    10     0     Mm2
..........................................................aaauuugaauuugcagcgUgauc...............................    1      1     Mm2
..........................................................aaauuugaauuugcagcgggauc...............................    26     0     Mm2
..........................................................aaauuugaauuugcagcgggGuc...............................    1      1     Mm2
..........................................................aaauuugaauuugcagcgggaucu..............................    3      0     Mm2

..................gGuacccuguagauccgaauuugu......................................................................    3      1     Mm1
...................auacccuguagaAccgaau..........................................................................    10     1     Mm1
...................auacccuguagaAccgaauuugu......................................................................    1      1     Mm1
...................auacccuguagauccgaauuugu......................................................................    4      0     Mm1
...................Cuacccuguagauccgaauuugu......................................................................    313    1     Mm1
....................uaGccuguagauccgaau..........................................................................    1      1     Mm1
....................uacAcuguagauccgaau..........................................................................    1      1     Mm1
....................uUcccuguagauccgaau..........................................................................    1      1     Mm1
....................uacccuguGgauccgaau..........................................................................    1      1     Mm1
....................uaccGuguagauccgaau..........................................................................    1      1     Mm1
....................uacccuguagCuccgaau..........................................................................    4      1     Mm1
....................uacccNguagauccgaau..........................................................................    1      1     Mm1
....................uacccuguagauccgaaC..........................................................................    17     1     Mm1
....................uacccuguagauccgaau..........................................................................    1958   0     Mm1
....................uacccuguagUuccgaau..........................................................................    3      1     Mm1
....................uaUccuguagauccgaau..........................................................................    1      1     Mm1
....................uacccuguagaucAgaau..........................................................................    1      1     Mm1
....................uacccuguagauUcgaau..........................................................................    3      1     Mm1
....................uacccuguagGuccgaau..........................................................................    2      1     Mm1
....................uacccuguagauccgaaG..........................................................................    13     1     Mm1
....................uacccuguagauccgaGu..........................................................................    5      1     Mm1
....................uacccCguagauccgaau..........................................................................    1      1     Mm1
....................uacccuguagauccgaUu..........................................................................    5      1     Mm1
....................uacccuguagauccgaaA..........................................................................    57     1     Mm1
....................uacccuguagaucUgaau..........................................................................    1      1     Mm1
....................uacccuguagauccgaCu..........................................................................    4      1     Mm1
....................uacccuguagauUcgaauu.........................................................................    4      1     Mm1
....................uacccuguaUauccgaauu.........................................................................    1      1     Mm1
....................uacccuguagGuccgaauu.........................................................................    4      1     Mm1
....................uGcccuguagauccgaauu.........................................................................    1      1     Mm1
....................uacUcuguagauccgaauu.........................................................................    1      1     Mm1
....................uacccuguagauccgaaGu.........................................................................    23     1     Mm1
....................uacccuguagauccgaaCu.........................................................................    19     1     Mm1
....................uacccuguagauccgaauA.........................................................................    107    1     Mm1
....................uacccuguagauccgaaAu.........................................................................    41     1     Mm1
....................uacccuguagCuccgaauu.........................................................................    5      1     Mm1
....................uacccuguagUuccgaauu.........................................................................    2      1     Mm1
....................Aacccuguagauccgaauu.........................................................................    1      1     Mm1
....................uacccuAuagauccgaauu.........................................................................    1      1     Mm1
....................uUcccuguagauccgaauu.........................................................................    3      1     Mm1
....................uacccuguagaucGgaauu.........................................................................    2      1     Mm1
....................uacAcuguagauccgaauu.........................................................................    1      1     Mm1
....................uacccuguagaucUgaauu.........................................................................    6      1     Mm1
....................uacccuguagauccgaGuu.........................................................................    6      1     Mm1
....................uacccuguagauccgaCuu.........................................................................    5      1     Mm1
....................uacccuguagauccgaauu.........................................................................    3324   0     Mm1
```

Mature **Star**

agaguucgcccaucagcagauacccuguagauccgaauuuguuugugaucgauaugacaaauuugaauuugcagcgggaucugaaguuggguauaaucaacugacaggagga			
....................uaAccuguagauccgaauu...	1	1	Mm1
....................uaUccuguagauccgaauu...	1	1	Mm1
....................uacccuguagauccgaUuu...	6	1	Mm1
....................uacccuguagauccgaauC...	1	1	Mm1
....................uaccUuguagauccgaauu...	1	1	Mm1
....................uacccuguagauccgaaGuu..	9	1	Mm1
....................uacccuguagauUcgaauuu..	1	1	Mm1
....................uacccuguagauccgaGuuu..	1	1	Mm1
....................Aacccuguagauccgaauuu..	1	1	Mm1
....................uacccuguagUuccgaauuu..	2	1	Mm1
....................uacAcuguagauccgaauuu..	1	1	Mm1
....................uacccuguGgauccgaauuu..	1	1	Mm1
....................uacUcuguagauccgaauuu..	1	1	Mm1
....................uacccuguagauccgaauAu..	28	1	Mm1
....................uacccuguagauccgaaAuu..	12	1	Mm1
....................uacccuguagauccgaauuu..	1607	0	Mm1
....................uacccuguagGuccgaauuu..	1	1	Mm1
....................uacccuguagauccgaCuuu..	5	1	Mm1
....................uacccuguagaucAgaauuu..	1	1	Mm1
....................uaAccuguagauccgaauuu..	1	1	Mm1
....................uacccuguagCuccgaauuu..	2	1	Mm1
....................uacccuguagauccgaUuuu..	5	1	Mm1
....................uacccuguagauccgUauuu..	1	1	Mm1
....................uacccuguagauccAaauuu..	1	1	Mm1
....................uaccAuguagauccgaauuu..	1	1	Mm1
....................uacccuguagauccgaaCuu..	8	1	Mm1
....................uacccuguagauccgaauuug...	4801	0	Mm1
....................uacccGguagauccgaauuug...	1	1	Mm1
....................uacccuguagauccgaaGuug...	12	1	Mm1
....................uacccuguagaucAgaauuug...	1	1	Mm1
....................uUcccuguagauccgaauuug...	2	1	Mm1
....................uacccAguagauccgaauuug...	2	1	Mm1
....................uacccuguagauccgaauuuU...	6	1	Mm1
....................uaccGuguagauccgaauuug...	1	1	Mm1
....................uacccuguagauccgaCuuug...	10	1	Mm1
....................Gacccuguagauccgaauuug...	1	1	Mm1
....................uacccuguagauccgGauuug...	4	1	Mm1
....................uacAcuguagauccgaauuug...	2	1	Mm1
....................uaAccuguagauccgaauuug...	1	1	Mm1
....................uacccuguagCuccgaauuug...	3	1	Mm1
....................uaGccuguagauccgaauuug...	1	1	Mm1
....................uacccuguagauccgaauuuA...	16	1	Mm1
....................uacccuguGgauccgaauuug...	1	1	Mm1
....................uacccuguagauccgaGuuug...	7	1	Mm1
....................uacccuguaUauccgaauuug...	1	1	Mm1
....................Cacccuguagauccgaauuug...	2	1	Mm1
....................uacccuguagGuccgaauuug...	3	1	Mm1
....................Nacccuguagauccgaauuug...	2	1	Mm1
....................uacccuguagauGcgaauuug...	1	1	Mm1
....................uaccAuguagauccgaauuug...	1	1	Mm1
....................uaUccuguagauccgaauuug...	1	1	Mm1
....................uacccuguagauccgaaAuug...	26	1	Mm1
....................uacccuguagauccgaUuuug...	10	1	Mm1
....................uacccuguagauccgaaCuug...	26	1	Mm1
....................uacccuguagaucUgaauuug...	7	1	Mm1
....................Aacccuguagauccgaauuug...	1	1	Mm1
....................uacccuguagUuccgaauuug...	2	1	Mm1
....................uacccugCagauccgaauuug...	1	1	Mm1
....................uacccuguagauUcgaauuug...	1	1	Mm1
....................uacccuguagauccgaauGugu..	33	1	Mm1
....................uacccuguagauccgaauuugA..	693	1	Mm1
....................Gacccuguagauccgaauuugu..	22	1	Mm1
....................uacccuguagauccgaauuuAu..	83	1	Mm1
....................uacccuguUgauccgaauuugu..	137	1	Mm1
....................uacccuCuagauccgaauuugu..	32	1	Mm1
....................uacccuguagUuccgaauuugu..	779	1	Mm1
....................uacccugCagauccgaauuugu..	131	1	Mm1
....................uacccuguagauccCaauuugu..	35	1	Mm1
....................uacccuguagauccgGauuugu..	117	1	Mm1
....................uCcccuguagauccgaauuugu..	70	1	Mm1

Mature **Star**

agaguucgcccaucagcagauacccuguagauccgaauuuguuugugaucgauaugacaaauuugaauuugcagcgggaucugaaguuggguauaaucaacugacaggagga			
....................uacccuUuagauccgaauuugu..	73	1	Mm1
....................uacccuguagauccgaaCuugu..	6485	1	Mm1
....................uacccuguagauccgaauuAgu..	66	1	Mm1
....................uaccAuguagauccgaauuugu..	180	1	Mm1
....................Cacccuguagauccgaauuugu..	201	1	Mm1
....................uacccuguagauccgaauNugu..	1	1	Mm1
....................uacccuguagauccgaaGuugu..	5368	1	Mm1
....................uacccCguagauccgaauuugu..	116	1	Mm1
....................uacccuguagauccgCauuugu..	83	1	Mm1
....................uacccuguagauccgaaAuugu..	4869	1	Mm1
....................uacccuguagGuccgaauuugu..	983	1	Mm1
....................uaNccuguagauccgaauuugu..	7	1	Mm1
....................uacccuguagauccgaauAugu..	43	1	Mm1
....................Nacccuguagauccgaauuugu..	495	1	Mm1
....................uacccuguGgauccgaauuugu..	135	1	Mm1
....................uacccuguagaucUgaauuugu..	475	1	Mm1
....................uacccuguagauAcgaauuugu..	45	1	Mm1
....................uacccuguagauccgaauuugG..	263	1	Mm1
....................uacccuguagaGccgaauuugu..	38	1	Mm1
....................uacccuguagauccgaauuuUu..	93	1	Mm1
....................uacccGguagauccgaauuugu..	54	1	Mm1
....................uacccuguaCauccgaauuugu..	28	1	Mm1
....................uacccuguagaucGgaauuugu..	47	1	Mm1
....................uacccuguaAauccgaauuugu..	71	1	Mm1
....................uacccuguagauccgaauuugu..	1021563	0	Mm1
....................uacccuguagauUcgaauuugu..	513	1	Mm1
....................uacccuguagaAccgaauuugu..	169	1	Mm1
....................uacccuguCgauccgaauuugu..	25	1	Mm1
....................uacccuguagauccgaauCugu..	81	1	Mm1
....................uacccuguagaCccgaauuugu..	113	1	Mm1
....................uacccuguaUauccgaauuugu..	91	1	Mm1
....................uacccuAuagauccgaauuugu..	95	1	Mm1
....................Aacccuguagauccgaauuugu..	85	1	Mm1
....................uacccuguagauccgaCuuugu..	2622	1	Mm1
....................uaccUuguagauccgaauuugu..	130	1	Mm1
....................uGcccuguagauccgaauuugu..	193	1	Mm1
....................uacccugAagauccgaauuugu..	71	1	Mm1
....................uNcccuguagauccgaauuugu..	9	1	Mm1
....................uacAcuguagauccgaauuugu..	414	1	Mm1
....................uacccuguagauccgaUuuugu..	1679	1	Mm1
....................uacccuguagCuccgaauuugu..	995	1	Mm1
....................uacccuguagauGcgaauuugu..	28	1	Mm1
....................uacccuguagauccgaauuGgu..	45	1	Mm1
....................uacUcuguagauccgaauuugu..	217	1	Mm1
....................uacccuguagauccUaauuugu..	84	1	Mm1
....................uacccNguagauccgaauuugu..	156	1	Mm1
....................uaUccuguagauccgaauuugu..	190	1	Mm1
....................uacccuguagauccgaGuuugu..	1775	1	Mm1
....................uacccuguagauccgaauuugC..	105	1	Mm1
....................uaccGuguagauccgaauuugu..	72	1	Mm1
....................uacccugGagauccgaauuugu..	28	1	Mm1
....................uaGccuguagauccgaauuugu..	258	1	Mm1
....................uacGcuguagauccgaauuugu..	85	1	Mm1
....................uacccAguagauccgaauuugu..	52	1	Mm1
....................uacccuNuagauccgaauuugu..	51	1	Mm1
....................uacccuguagauccgaaNuugu..	2	1	Mm1
....................uUcccuguagauccgaauuugu..	228	1	Mm1
....................uacccuguagauccgUauuugu..	279	1	Mm1
....................uacccuguagauccgaauuuCu..	24	1	Mm1
....................uacccuguagauccAaauuugu..	99	1	Mm1
....................uacccuguagauccgaauuCgu..	100	1	Mm1
....................uaAccuguagauccgaauuugu..	236	1	Mm1
....................uacccuguagaucAgaauuugu..	222	1	Mm1
....................uacccuUuagauccgaauuuguu...	4	1	Mm1
....................uacccuguagauccAaauuuguu...	1	1	Mm1
....................uacccuguagauccgaaGuuguu...	110	1	Mm1
....................uacccuguGgauccgaauuuguu...	3	1	Mm1
....................uacccuguagauccgaauuuguG...	1	1	Mm1
....................uacAcuguagauccgaauuuguu...	10	1	Mm1
....................uacGcuguagauccgaauuuguu...	1	1	Mm1

Mature Star

agaguucgcccaucagcagauacccuguagauccgaauuuguuugugaucgauaugacaaauuugaauuugcagcgggaucugaaguuggguauaaucaacugacaggagga			
....................uacccuguagauccgaauCuguu...	1	1	Mm1
....................uacccuguaCauccgaauuuguu...	1	1	Mm1
....................uacccuguaUauccgaauuuguu...	1	1	Mm1
....................uacccuguagauUcgaauuuguu...	6	1	Mm1
....................uacccuguagauccgaauuugGu...	1	1	Mm1
....................uacccuguagauccgaUuuuguu...	30	1	Mm1
....................uacccuguagauccgaauuuguC...	11	1	Mm1
....................uacccuguagCuccgaauuuguu...	23	1	Mm1
....................uacccuguagauccgaGuuuguu...	30	1	Mm1
....................uacccCguagauccgaauuuguu...	2	1	Mm1
....................uacccuguagauccgUauuuguu...	6	1	Mm1
....................uacccugAagauccgaauuuguu...	1	1	Mm1
....................uaccGuguagauccgaauuuguu...	1	1	Mm1
....................uUccuguagauccgaauuuguu...	5	1	Mm1
....................uacccuguagauccgGauuuguu...	1	1	Mm1
....................Cacccuguagauccgaauuuguu...	3	1	Mm1
....................uacccugCagauccgaauuuguu...	1	1	Mm1
....................uacccuguagauccgaauuuUuu...	5	1	Mm1
....................uacccuguCgauccgaauuuguu...	2	1	Mm1
....................uacccNguagauccgaauuuguu...	1	1	Mm1
....................uaAccuguagauccgaauuuguu...	3	1	Mm1
....................uacccuAuagauccgaauuuguu...	2	1	Mm1
....................uacccuguagauccgaauuugCu...	3	1	Mm1
....................uacccuguagauccgaauuugAu...	4	1	Mm1
....................uacccuguagauccgaauuGguu...	1	1	Mm1
....................uaUccuguagauccgaauuuguu...	10	1	Mm1
....................uacccuguagaAccgaauuuguu...	3	1	Mm1
....................Nacccuguagauccgaauuuguu...	5	1	Mm1
....................uaGccuguagauccgaauuuguu...	3	1	Mm1
....................uacccuguagauGcgaauuuguu...	2	1	Mm1
....................uCcccuguagauccgaauuuguu...	2	1	Mm1
....................uacccuguUgauccgaauuuguu...	6	1	Mm1
....................uacccuguagauccgaauuCguu...	5	1	Mm1
....................uacccuguagaGccgaauuuguu...	1	1	Mm1
....................uacccuguagaucUgaauuuguu...	21	1	Mm1
....................uacUcuguagauccgaauuuguu...	1	1	Mm1
....................uacccuCuagauccgaauuuguu...	3	1	Mm1
....................uacccuguagauccgaauuAguu...	2	1	Mm1
....................uacccuguagauccgaaCuuguu...	117	1	Mm1
....................uacccAguagauccgaauuuguu...	1	1	Mm1
....................uacccuguagauccgaaAuuguu...	100	1	Mm1
....................uaccUuguagauccgaauuuguu...	2	1	Mm1
....................uacccuguagauccgaauuuAuu...	3	1	Mm1
....................uacccuNuagauccgaauuuguu...	1	1	Mm1
....................uacccuguagUuccgaauuuguu...	10	1	Mm1
....................uacccuguagauccgaauuuguA...	1724	1	Mm1
....................uacccuguagauccgaauuuguu...	19124	0	Mm1
....................uGcccuguagauccgaauuuguu...	4	1	Mm1
....................uacccuguaAauccgaauuuguu...	2	1	Mm1
....................uacccuguagauccgaCuuuguu...	45	1	Mm1
....................uacccuguagGuccgaauuuguu...	10	1	Mm1
....................uacccuguagUuccgaauuuguuu..	1	1	Mm1
....................uGcccuguagauccgaauuuguuu..	1	1	Mm1
....................uacccuguagauccgaauuuguuA..	162	1	Mm1
....................uacccuguagauccgaaCuuguuu..	10	1	Mm1
....................uacccuguagauccgaauuuguAu..	1172	1	Mm1
....................uacccuguagauccgaUuuuguuu..	1	1	Mm1
....................uacccuguagauccgaGuuuguuu..	2	1	Mm1
....................uacccuguagauccgaauuuguCu..	15	1	Mm1
....................uacccuguagaucUgaauuuguuu..	1	1	Mm1
....................uacccuguagauccgaauuuguuu..	1029	0	Mm1
....................uacccuguagGuccgaauuuguuu..	1	1	Mm1
....................uacccuguagauccgaCuuuguuu..	4	1	Mm1
....................uacccuguagaCccgaauuuguuu..	1	1	Mm1
....................uacccuguagaucAgaauuuguuu..	1	1	Mm1
....................uacccuguagauccgaauuuguuG..	1	1	Mm1
....................uacccuguagauccgaaAuuguuu..	4	1	Mm1
....................uacccuguagauccgaaGuuguuu..	10	1	Mm1
....................uacccuguagauccgaauuuguGu..	8	1	Mm1
....................uacccuguagauccgaauuuguuuA...	2	1	Mm1

Mature Star

agaguucgcccaucagcagauacccuguagauccgaauuuguuugugaucgauaugacaaauuugaauuugcagcgggaucugaaguuggguauaaucaacugacaggagga			
....................uacccuguagauccgaauuuguuuU...	1	1	Mm1
....................uacccuguagauccgaauuuguuugu..	1	0	Mm1
....................uacccuguagauccgaauuuguuuguU...	1	1	Mm1
....................uacccuguagauccgaauuuguuugug...	1	0	Mm1
....................uacccuguagauccgaauuuguuugugau...	2	0	Mm1
.....................acccuguagauccgaauu...	8	0	Mm1
.....................accUuguagauccgaauu...	3	1	Mm1
.....................acccuguagauccgaauAu..	2	1	Mm1
.....................acccuguagaAccgaauuu..	1	1	Mm1
.....................acccuguagauccgaauuu..	6	0	Mm1
.....................acccuguagauccgaaCuug...	1	1	Mm1
.....................acccuguagauccgaauGug...	1	1	Mm1
.....................acccuguagauccgaauuug...	18	0	Mm1
.....................acccuguagauccgaauuuA...	1	1	Mm1
.....................acccuguagauccgaauGugu..	14	1	Mm1
.....................acccuguagauccgUauuugu..	7	1	Mm1
.....................acccCguagauccgaauuugu..	6	1	Mm1
.....................acccuguagauccCaauuugu..	2	1	Mm1
.....................acccuguagauccgaauuugC..	1	1	Mm1
.....................acccuguagauccgaauuuAu..	6	1	Mm1
.....................acccuguagauccgaauuuUu..	6	1	Mm1
.....................acAcuguagauccgaauuugu..	1	1	Mm1
.....................acccuguagauccgCauuugu..	5	1	Mm1
.....................acccugAagauccgaauuugu..	8	1	Mm1
.....................acccuguagauccgaauuugA..	15	1	Mm1
.....................acccuguagaucUgaauuugu..	4	1	Mm1
.....................acccuguagauccgaUuuugu..	7	1	Mm1
.....................accAuguagauccgaauuugu..	6	1	Mm1
.....................acccuguagauUcgaauuugu..	5	1	Mm1
.....................acccAguagauccgaauuugu..	1	1	Mm1
.....................accGuguagauccgaauuugu..	2	1	Mm1
.....................acccuguagauccgaauuuCu..	10	1	Mm1
.....................acccuguagUuccgaauuugu..	1	1	Mm1
.....................acccuUuagauccgaauuugu..	1	1	Mm1
.....................acccuguagauccgaauCugu..	38	1	Mm1
.....................acccugGagauccgaauuugu..	1	1	Mm1
.....................acccuAuagauccgaauuugu..	2	1	Mm1
.....................acccuguagaucGgaauuugu..	2	1	Mm1
.....................acccuguagauccgaauuugG..	4	1	Mm1
.....................aGccuguagauccgaauuugu..	1	1	Mm1
.....................acccuguagGuccgaauuugu..	1	1	Mm1
.....................acccuguagauccgaCuuugu..	20	1	Mm1
.....................acccuguCgauccgaauuugu..	1	1	Mm1
.....................acccuguGgauccgaauuugu..	4	1	Mm1
.....................acGcuguagauccgaauuugu..	3	1	Mm1
.....................Ucccuguagauccgaauuugu..	7	1	Mm1
.....................acccuCuagauccgaauuugu..	1	1	Mm1
.....................acccugCagauccgaauuugu..	16	1	Mm1
.....................acccuguagauccAaauuugu..	4	1	Mm1
.....................acccuguagauccgaauuCgu..	1	1	Mm1
.....................acccuguagauccgaauAugu..	10	1	Mm1
.....................acccuguagauccgaauuugu..	5416	0	Mm1
.....................acccuguagaGccgaauuugu..	16	1	Mm1
.....................acccuguaAauccgaauuugu..	2	1	Mm1
.....................acccuguagauccgaaAuugu..	4	1	Mm1
.....................acccuguagauccgaauuGgu..	3	1	Mm1
.....................acccuguagauccgaaGuugu..	8	1	Mm1
.....................acccuguagaAccgaauuugu..	16	1	Mm1
.....................acccuguagaucAgaauuugu..	8	1	Mm1
.....................acccuguaCauccgaauuugu..	4	1	Mm1
.....................acccuguagauccgGauuugu..	7	1	Mm1
.....................Ncccuguagauccgaauuugu..	3	1	Mm1
.....................acccuguUgauccgaauuugu..	1	1	Mm1
.....................acccuguagaCccgaauuugu..	1	1	Mm1
.....................acccuguagauccgaGuuugu..	10	1	Mm1
.....................accUuguagauccgaauuugu..	3	1	Mm1
.....................acccuguagauccgaaCuugu..	2	1	Mm1
.....................acccuNuagauccgaauuugu..	1	1	Mm1
.....................acccuguagauccUaauuugu..	1	1	Mm1
.....................acccuguaUauccgaauuuguu...	1	1	Mm1

```
                    Mature                                Star
agaguucgcccaucagcagauacccuguagauccgaauuuguuugugaucgauaugacaaauuugaauuugcagcgggaucugaaguuggguauaaucaacugacaggagga
.....................acccuguagauccgaauAuguu.....................................................................    1     1    Mm1
.....................acccuguagauccgaauuuguu.....................................................................    213   0    Mm1
.....................acAcuguagauccgaauuuguu.....................................................................    1     1    Mm1
.....................acccuguagauccgaauCuguu.....................................................................    4     1    Mm1
.....................acccuguagauccgaauuuguA.....................................................................    5     1    Mm1
.....................acccuguagaCccgaauuuguu.....................................................................    1     1    Mm1
.....................acccuguagauccgaauuuguAu....................................................................    16    1    Mm1
.....................acccuguagauccgaauuuguuA....................................................................    2     1    Mm1
.....................acccuguagauccgaauuuguuu....................................................................    7     0    Mm1
......................cccuguagauccgaauuug.......................................................................    2     0    Mm1
......................cccugCagauccgaauuugu......................................................................    1     1    Mm1
......................cccuguagCuccgaauuugu......................................................................    1     1    Mm1
......................cccuguagUuccgaauuugu......................................................................    4     1    Mm1
......................cccuguagaucGgaauuugu......................................................................    1     1    Mm1
......................cccuguagauccgaauuAgu......................................................................    1     1    Mm1
......................cccuguagauccgaauuugu......................................................................    286   0    Mm1
......................cccGguagauccgaauuugu......................................................................    3     1    Mm1
......................cccuguagauccgaUuuugu......................................................................    1     1    Mm1
......................cccuguagauccgaauuuUu......................................................................    8     1    Mm1
......................cccuguagauccgaauGugu......................................................................    1     1    Mm1
......................ccUuguagauccgaauuugu......................................................................    2     1    Mm1
......................cccuguagauccgaauuCgu......................................................................    2     1    Mm1
......................cccuguaAauccgaauuugu......................................................................    3     1    Mm1
......................cccuguagauccgaauuGgu......................................................................    4     1    Mm1
......................cccuguagauccgaauuuguu.....................................................................    9     0    Mm1
......................cccuguagauccgaauuuguA.....................................................................    1     1    Mm1
......................cccuguagauccgaauuuUuuu....................................................................    1     1    Mm1
......................cccuguagauccgaauuuguAu....................................................................    3     1    Mm1
......................cccuguagauccgaauuuguuu....................................................................    2     0    Mm1
.......................ccuguagauccgaauuugu......................................................................    29    0    Mm1

.gaguucgcccaucagcaga............................................................................................    7     0    Fm3
...................auacccuguagaAccgaa...........................................................................    35    1    Fm3
...................auacccuguagaAccgaau..........................................................................    50    1    Fm3
...................Cuacccuguagauccgaau..........................................................................    2     1    Fm3
...................auacccuguagaAccgaauu.........................................................................    5     1    Fm3
...................Cuacccuguagauccgaauu.........................................................................    11    1    Fm3
...................Cuacccuguagauccgaauuu........................................................................    9     1    Fm3
...................auacccuguagaAccgaauuu........................................................................    1     1    Fm3
...................Cuacccuguagauccgaauuug.......................................................................    22    1    Fm3
...................auacccuguagauccgaaCuugu......................................................................    1     1    Fm3
...................auacccuguagauccgUauuugu......................................................................    1     1    Fm3
...................Cuacccuguagauccgaauuugu......................................................................    1889  1    Fm3
...................auacccuguagauccgCauuugu......................................................................    1     1    Fm3
...................auacccuguagauccgaGuuugu......................................................................    1     1    Fm3
...................auacccuguagauccgaauuugu......................................................................    94    0    Fm3
...................auacccuguagaAccgaauuugu......................................................................    13    1    Fm3
...................Guacccuguagauccgaauuugu......................................................................    5     1    Fm3
...................Uuacccuguagauccgaauuugu......................................................................    3     1    Fm3
...................Cuacccuguagauccgaauuuguu.....................................................................    7     1    Fm3
....................uaAccuguagauccgaau..........................................................................    3     1    Fm3
....................uacccuguagauUcgaau..........................................................................    28    1    Fm3
....................uacccuguagauccgaaA..........................................................................    382   1    Fm3
....................uacccuguUgauccgaau..........................................................................    4     1    Fm3
....................uacAcuguagauccgaau..........................................................................    4     1    Fm3
....................Nacccuguagauccgaau..........................................................................    7     1    Fm3
....................uacccuguagauAcgaau..........................................................................    2     1    Fm3
....................uacccuguagaucAgaau..........................................................................    6     1    Fm3
....................uacccuguagaucGgaau..........................................................................    4     1    Fm3
....................Cacccuguagauccgaau..........................................................................    2     1    Fm3
....................Aacccuguagauccgaau..........................................................................    5     1    Fm3
....................uacccuguagauccAaau..........................................................................    1     1    Fm3
....................uacccuguagauccgaUu..........................................................................    28    1    Fm3
....................uacccuCuagauccgaau..........................................................................    2     1    Fm3
....................uacGcuguagauccgaau..........................................................................    1     1    Fm3
....................uacccuguagauccgGau..........................................................................    1     1    Fm3
....................uacccuguagGuccgaau..........................................................................    12    1    Fm3
....................uacccuguagauccgaaG..........................................................................    62    1    Fm3
....................uacccuguagauccgaCu..........................................................................    27    1    Fm3
....................uGcccuguagauccgaau..........................................................................    4     1    Fm3
```

```
                    Mature                              Star
agaguucgcccaucagcagauacccuguagauccgaauuuguuugugaucgauaugacaaauuugaauuugcagcgggaucugaaguuggguauaaucaacugacaggagga
....................uacccuguagaCccgaau..........................................................................  2      1  Fm3
....................uaccUuguagauccgaau..........................................................................  3      1  Fm3
....................uacccuguagauccgaau..........................................................................  11459  0  Fm3
....................uacccGguagauccgaau..........................................................................  5      1  Fm3
....................uacccugGagauccgaau..........................................................................  1      1  Fm3
....................uUcccuguagauccgaau..........................................................................  4      1  Fm3
....................uacccuguagaAccgaau..........................................................................  23     1  Fm3
....................uacccuguagauccUaau..........................................................................  1      1  Fm3
....................uacccuguGgauccgaau..........................................................................  2      1  Fm3
....................uacccugCagauccgaau..........................................................................  2      1  Fm3
....................uacccuguagauccgaGu..........................................................................  16     1  Fm3
....................uaUccuguagauccgaau..........................................................................  1      1  Fm3
....................uacccuguagUuccgaau..........................................................................  9      1  Fm3
....................uaccAuguagauccgaau..........................................................................  5      1  Fm3
....................uacUcuguagauccgaau..........................................................................  3      1  Fm3
....................uacccAguagauccgaau..........................................................................  1      1  Fm3
....................uacccuguaUauccgaau..........................................................................  1      1  Fm3
....................uacccuguagauccgaaC..........................................................................  55     1  Fm3
....................uaGccuguagauccgaau..........................................................................  3      1  Fm3
....................uCcccuguagauccgaau..........................................................................  2      1  Fm3
....................uacccuguagaucUgaau..........................................................................  45     1  Fm3
....................uacccuAuagauccgaau..........................................................................  4      1  Fm3
....................uacccuguaAauccgaau..........................................................................  1      1  Fm3
....................uacccNguagauccgaau..........................................................................  1      1  Fm3
....................uacccuguaCauccgaau..........................................................................  1      1  Fm3
....................uacccuguagCuccgaau..........................................................................  11     1  Fm3
....................uacccugAagauccgaau..........................................................................  1      1  Fm3
....................uacccugAagauccgaauu.........................................................................  2      1  Fm3
....................uacccuguagaucUgaauu.........................................................................  16     1  Fm3
....................uacccuAuagauccgaauu.........................................................................  2      1  Fm3
....................uacccuguagaucAgaauu.........................................................................  10     1  Fm3
....................uacccuguagauccgaCuu.........................................................................  36     1  Fm3
....................uacccNguagauccgaauu.........................................................................  3      1  Fm3
....................uacccuCuagauccgaauu.........................................................................  3      1  Fm3
....................uacccuguagGuccgaauu.........................................................................  17     1  Fm3
....................uacGcuguagauccgaauu.........................................................................  1      1  Fm3
....................uacccuguaUauccgaauu.........................................................................  1      1  Fm3
....................uaAccuguagauccgaauu.........................................................................  6      1  Fm3
....................uacccGguagauccgaauu.........................................................................  7      1  Fm3
....................uacccuguagauccgaaCu.........................................................................  110    1  Fm3
....................uacccuguagCuccgaauu.........................................................................  20     1  Fm3
....................uacccuguagauccgGauu.........................................................................  3      1  Fm3
....................uaUccuguagauccgaauu.........................................................................  6      1  Fm3
....................uCcccuguagauccgaauu.........................................................................  1      1  Fm3
....................Cacccuguagauccgaauu.........................................................................  1      1  Fm3
....................uacccuguUgauccgaauu.........................................................................  8      1  Fm3
....................uacccuguagauccgaauu.........................................................................  17784  0  Fm3
....................uacAcuguagauccgaauu.........................................................................  14     1  Fm3
....................uacccuguagauccgaaGu.........................................................................  113    1  Fm3
....................uacccuguaAauccgaauu.........................................................................  2      1  Fm3
....................uaGccuguagauccgaauu.........................................................................  3      1  Fm3
....................uGcccuguagauccgaauu.........................................................................  3      1  Fm3
....................Gacccuguagauccgaauu.........................................................................  1      1  Fm3
....................Aacccuguagauccgaauu.........................................................................  1      1  Fm3
....................uacccuguagauccAaauu.........................................................................  7      1  Fm3
....................uacccAguagauccgaauu.........................................................................  1      1  Fm3
....................uaccGuguagauccgaauu.........................................................................  2      1  Fm3
....................uacccuguagauUcgaauu.........................................................................  30     1  Fm3
....................uacccuguagauccgaauA.........................................................................  426    1  Fm3
....................uacccuguagauccgCauu.........................................................................  3      1  Fm3
....................uaccAuguagauccgaauu.........................................................................  9      1  Fm3
....................uUcccuguagauccgaauu.........................................................................  5      1  Fm3
....................uacccuguagauccgUauu.........................................................................  3      1  Fm3
....................Nacccuguagauccgaauu.........................................................................  11     1  Fm3
....................uaccUuguagauccgaauu.........................................................................  6      1  Fm3
....................uacccuguagaAccgaauu.........................................................................  3      1  Fm3
....................uacccuguagaucGgaauu.........................................................................  2      1  Fm3
....................uacccuguagaCccgaauu.........................................................................  3      1  Fm3
....................uacccuguagauccUaauu.........................................................................  4      1  Fm3
....................uacccuguagauccgaGuu.........................................................................  26     1  Fm3
```

Mature **Star**

agaguucgcccaucagcagauacccuguagauccgaauuuguuugugaucgauaugacaaauuugaauuugcagcgggaucugaaguuggguauaaucaacugacaggagga			
....................uacccuguagUuccgaauu...	15	1	Fm3
....................uacccuguagauccgaUuu...	31	1	Fm3
....................uacUcuguagauccgaauu...	6	1	Fm3
....................uacccuguagauccgaaAu...	164	1	Fm3
....................uacccuguagauccgaUuuu..	26	1	Fm3
....................uacccuguagauAcgaauuu..	6	1	Fm3
....................uacccuguagauccgaGuuu..	23	1	Fm3
....................uacccuAuagauccgaauuu..	4	1	Fm3
....................uacccuUuagauccgaauuu..	2	1	Fm3
....................uacGcuguagauccgaauuu..	1	1	Fm3
....................uacccuguagaAccgaauuu..	2	1	Fm3
....................uacccuguagauccgaauGu..	1	1	Fm3
....................uacccuguGgauccgaauuu..	4	1	Fm3
....................uaAccuguagauccgaauuu..	2	1	Fm3
....................Aacccuguagauccgaauuu..	5	1	Fm3
....................Nacccuguagauccgaauuu..	9	1	Fm3
....................uacccuguagaGccgaauuu..	1	1	Fm3
....................uacccuguagaucAgaauuu..	7	1	Fm3
....................uacccuguagauccAaauuu..	9	1	Fm3
....................uacccuguagauccgUauuu..	8	1	Fm3
....................uacccuguagauccgaCuuu..	51	1	Fm3
....................uacUcuguagauccgaauuu..	3	1	Fm3
....................uGcccuguagauccgaauuu..	4	1	Fm3
....................uacccuguagGuccgaauuu..	17	1	Fm3
....................uacccuguagauccgaauuC..	2	1	Fm3
....................uacccGguagauccgaauuu..	4	1	Fm3
....................uaccGuguagauccgaauuu..	2	1	Fm3
....................uacccuguagaucUgaauuu..	16	1	Fm3
....................uaGccuguagauccgaauuu..	7	1	Fm3
....................uacccuguagaCccgaauuu..	2	1	Fm3
....................uacccuguagaucGgaauuu..	1	1	Fm3
....................uacccuNuagauccgaauuu..	1	1	Fm3
....................Gacccuguagauccgaauuu..	1	1	Fm3
....................uacccuguagauGcgaauuu..	1	1	Fm3
....................uacccuguagauccgaauuA..	147	1	Fm3
....................uacccuguaUauccgaauuu..	2	1	Fm3
....................uacccuguagauccgGauuu..	2	1	Fm3
....................uacccuguagCuccgaauuu..	19	1	Fm3
....................uacccuguagauccUaauuu..	4	1	Fm3
....................uacccuguagauccgaauuu..	19231	0	Fm3
....................uacccuguagauccgaaGuu..	93	1	Fm3
....................uacccuguagauccgaauCu..	1	1	Fm3
....................uacAcuguagauccgaauuu..	12	1	Fm3
....................uacccuguUgauccgaauuu..	5	1	Fm3
....................uacccuguaAauccgaauuu..	2	1	Fm3
....................uacccugAagauccgaauuu..	4	1	Fm3
....................uacccuCuagauccgaauuu..	5	1	Fm3
....................uacccuguagauccgaauAu..	100	1	Fm3
....................uacccuguagauccgaaCuu..	121	1	Fm3
....................Cacccuguagauccgaauuu..	6	1	Fm3
....................uaUccuguagauccgaauuu..	3	1	Fm3
....................uacccuguagauccgaaAuu..	96	1	Fm3
....................uUcccuguagauccgaauuu..	7	1	Fm3
....................uacccuguagauccgCauuu..	1	1	Fm3
....................uCcccuguagauccgaauuu..	2	1	Fm3
....................uacccuguagauUcgaauuu..	35	1	Fm3
....................uacccuguagauccgaauuG..	7	1	Fm3
....................uacccugCagauccgaauuu..	2	1	Fm3
....................uacccCguagauccgaauuu..	4	1	Fm3
....................uaccAuguagauccgaauuu..	14	1	Fm3
....................uacccAguagauccgaauuu..	3	1	Fm3
....................uacccuguagUuccgaauuu..	17	1	Fm3
....................uacccuguGgauccgaauuug...	18	1	Fm3
....................uacccuguagauccgaauuuU...	166	1	Fm3
....................uacccAguagauccgaauuug...	3	1	Fm3
....................uGcccuguagauccgaauuug...	13	1	Fm3
....................uacccuguagaCccgaauuug...	9	1	Fm3
....................uacccuguagauccgaauuGg...	2	1	Fm3
....................uacccuguagauccgUauuug...	17	1	Fm3
....................uUcccuguagauccgaauuug...	12	1	Fm3

```
                    Mature                                Star
agaguucgcccaucagcagauacccuguagauccgaauuuguuugugaucgauaugacaaauuugaauuugcagcgggaucugaaguuggguauaaucaacugacaggagga
....................uacccuguagauccgaGuuug.......................................................................   111     1   Fm3
....................uacccuguagauccgaaGuug.......................................................................   381     1   Fm3
....................uacccuguagauccgaauuuC.......................................................................   4       1   Fm3
....................uacccuguagauccgaauuCg.......................................................................   9       1   Fm3
....................Gacccuguagauccgaauuug.......................................................................   2       1   Fm3
....................uacccuAuagauccgaauuug.......................................................................   10      1   Fm3
....................uaccUuguagauccgaauuug.......................................................................   10      1   Fm3
....................uacccuguagauccgaaCuug.......................................................................   469     1   Fm3
....................uacccNguagauccgaauuug.......................................................................   10      1   Fm3
....................uacccGguagauccgaauuug.......................................................................   8       1   Fm3
....................uNcccuguagauccgaauuug.......................................................................   2       1   Fm3
....................uacccuguagaucUgaauuug.......................................................................   73      1   Fm3
....................uacccuguagauccgGauuug.......................................................................   8       1   Fm3
....................uaAccuguagauccgaauuug.......................................................................   12      1   Fm3
....................uacccuguagUuccgaauuug.......................................................................   43      1   Fm3
....................uaccAuguagauccgaauuug.......................................................................   32      1   Fm3
....................uacGcuguagauccgaauuug.......................................................................   6       1   Fm3
....................uacccuguagaucGgaauuug.......................................................................   4       1   Fm3
....................uacccuguagauccgaUuuug.......................................................................   112     1   Fm3
....................Aacccuguagauccgaauuug.......................................................................   3       1   Fm3
....................uacccuCuagauccgaauuug.......................................................................   10      1   Fm3
....................uacccuguagauccgaauCug.......................................................................   9       1   Fm3
....................uaccGuguagauccgaauuug.......................................................................   9       1   Fm3
....................uacccuguagauGcgaauuug.......................................................................   2       1   Fm3
....................Nacccuguagauccgaauuug.......................................................................   32      1   Fm3
....................uaUccuguagauccgaauuug.......................................................................   21      1   Fm3
....................uaGccuguagauccgaauuug.......................................................................   17      1   Fm3
....................uacccuguagGuccgaauuug.......................................................................   81      1   Fm3
....................uacccuguagaAccgaauuug.......................................................................   7       1   Fm3
....................uacccuguaUauccgaauuug.......................................................................   10      1   Fm3
....................uacccuguagauUcgaauuug.......................................................................   35      1   Fm3
....................uacccuguagauccgaauuuA.......................................................................   306     1   Fm3
....................uacccugAagauccgaauuug.......................................................................   5       1   Fm3
....................uacccuguCgauccgaauuug.......................................................................   4       1   Fm3
....................uacccugCagauccgaauuug.......................................................................   12      1   Fm3
....................Cacccuguagauccgaauuug.......................................................................   19      1   Fm3
....................uacccuNuagauccgaauuug.......................................................................   7       1   Fm3
....................uacccuUuagauccgaauuug.......................................................................   7       1   Fm3
....................uacccCguagauccgaauuug.......................................................................   7       1   Fm3
....................uacUcuguagauccgaauuug.......................................................................   12      1   Fm3
....................uacAcuguagauccgaauuug.......................................................................   36      1   Fm3
....................uacccuguagCuccgaauuug.......................................................................   82      1   Fm3
....................uacccuguagauccgaCuuug.......................................................................   179     1   Fm3
....................uacccuguagauccgaauuug.......................................................................   71750   0   Fm3
....................uacccuguagauAcgaauuug.......................................................................   3       1   Fm3
....................uacccuguagauccAaauuug.......................................................................   12      1   Fm3
....................uacccuguagauccgaauGug.......................................................................   1       1   Fm3
....................uacccuguUgauccgaauuug.......................................................................   14      1   Fm3
....................uCcccuguagauccgaauuug.......................................................................   3       1   Fm3
....................uacccuguaCauccgaauuug.......................................................................   2       1   Fm3
....................uacccuguaAauccgaauuug.......................................................................   12      1   Fm3
....................uacccugGagauccgaauuug.......................................................................   1       1   Fm3
....................uacccuguagaucAgaauuug.......................................................................   40      1   Fm3
....................uacccuguagauccgCauuug.......................................................................   4       1   Fm3
....................uacccuguagauccgaaAuug.......................................................................   336     1   Fm3
....................uacccuguagauccCaauuug.......................................................................   6       1   Fm3
....................uacccuguagauccUaauuug.......................................................................   7       1   Fm3
....................uacccuguagauUcgaauuugu......................................................................   2397    1   Fm3
....................uacccCguagauccgaauuugu......................................................................   618     1   Fm3
....................uacccuguagauccgaaCuugu......................................................................   31807   1   Fm3
....................uaccAuguagauccgaauuugu......................................................................   3006    1   Fm3
....................uacccuguagauccgaauuuCu......................................................................   146     1   Fm3
....................Aacccuguagauccgaauuugu......................................................................   421     1   Fm3
....................uacccuguagGuccgaauuugu......................................................................   5297    1   Fm3
....................uacccuNuagauccgaauuugu......................................................................   313     1   Fm3
....................uacccuguagauccgaauNugu......................................................................   3       1   Fm3
....................uacccuguagauccgCauuugu......................................................................   282     1   Fm3
....................uacccuguagaucUgaauuugu......................................................................   3748    1   Fm3
....................uacccuguagaucAgaauuugu......................................................................   2092    1   Fm3
....................uacccuguagauccgUauuugu......................................................................   1256    1   Fm3
```

Mature Star

agaguucgcccaucagcagauacccuguagauccgaauuuguuugugaucgauaugacaaauuugaauuugcagcgggaucugaaguuggguauaaucaacugacaggagga			
....................uacccuguagauccgaauuugu..	5108096	0	Fm3
....................uacccNguagauccgaauuugu..	859	1	Fm3
....................uacccuguagauGcgaauuugu..	227	1	Fm3
....................uacccuguagaucGgaauuugu..	429	1	Fm3
....................uacUcuguagauccgaauuugu..	1149	1	Fm3
....................uacccuguagauccgaauuCgu..	502	1	Fm3
....................uacccugGagauccgaauuugu..	112	1	Fm3
....................uacccuguagauccgaauuugA..	4396	1	Fm3
....................uacccuguagauccAaauuugu..	1007	1	Fm3
....................uacGcuguagauccgaauuugu..	516	1	Fm3
....................uaGccuguagauccgaauuugu..	1439	1	Fm3
....................uacccuguagauccgaauGugu..	152	1	Fm3
....................uacccuguagaAccgaauuugu..	508	1	Fm3
....................Nacccuguagauccgaauuugu..	2577	1	Fm3
....................uacccuguagauccgaauuAgu..	336	1	Fm3
....................uacccuUuagauccgaauuugu..	448	1	Fm3
....................uacccuguagauccgaauCugu..	558	1	Fm3
....................uacccuguagauccgaNuuugu..	7	1	Fm3
....................uacccuguagauccgaUuuugu..	8001	1	Fm3
....................uacccuguUgauccgaauuugu..	1174	1	Fm3
....................uacccuguagCuccgaauuugu..	4869	1	Fm3
....................Cacccuguagauccgaauuugu..	1099	1	Fm3
....................uacccuguagauccgGauuugu..	620	1	Fm3
....................uNcccuguagauccgaauuugu..	39	1	Fm3
....................uacccuguagauccgaGuuugu..	9534	1	Fm3
....................uacccuguagauccgaauuGgu..	175	1	Fm3
....................uacccuguagUuccgaauuugu..	4200	1	Fm3
....................uacccuguagauccgaauAugu..	205	1	Fm3
....................uacccuguagauccgaaGuugu..	27198	1	Fm3
....................uaNccuguagauccgaauuugu..	23	1	Fm3
....................uaccGuguagauccgaauuugu..	377	1	Fm3
....................uacccuguaCauccgaauuugu..	166	1	Fm3
....................uaccUuguagauccgaauuugu..	841	1	Fm3
....................uacccuguagauccgaauuugC..	421	1	Fm3
....................uaAccuguagauccgaauuugu..	1176	1	Fm3
....................uaUccuguagauccgaauuugu..	1220	1	Fm3
....................uacccuguaAauccgaauuugu..	285	1	Fm3
....................uacccuguagauccgaaAuugu..	24287	1	Fm3
....................uacccuguagauccgaCuuugu..	12700	1	Fm3
....................uacccuguagauccCaauuugu..	209	1	Fm3
....................Gacccuguagauccgaauuugu..	107	1	Fm3
....................uacccuCuagauccgaauuugu..	642	1	Fm3
....................uacccuguCgauccgaauuugu..	194	1	Fm3
....................uacccuguagaCccgaauuugu..	790	1	Fm3
....................uacccAguagauccgaauuugu..	322	1	Fm3
....................uacccuguagauccgaauuuUu..	437	1	Fm3
....................uGcccuguagauccgaauuugu..	1199	1	Fm3
....................uCcccuguagauccgaauuugu..	263	1	Fm3
....................uacccuguagauccgaaNuugu..	8	1	Fm3
....................uacccuguagauccgaauuuAu..	451	1	Fm3
....................uacccugAagauccgaauuugu..	420	1	Fm3
....................uacccuguagauccgaauuugG..	119	1	Fm3
....................uacccuguagauccUaauuugu..	736	1	Fm3
....................uacccuguagaGccgaauuugu..	125	1	Fm3
....................uacAcuguagauccgaauuugu..	3086	1	Fm3
....................uacccuguGgauccgaauuugu..	889	1	Fm3
....................uacccuguagauAcgaauuugu..	567	1	Fm3
....................uUcccuguagauccgaauuugu..	839	1	Fm3
....................uacccuguaUauccgaauuugu..	549	1	Fm3
....................uacccGguagauccgaauuugu..	362	1	Fm3
....................uacccugCagauccgaauuugu..	756	1	Fm3
....................uacccuAuagauccgaauuugu..	927	1	Fm3
....................uacccuNuagauccgaauuuguu...	2	1	Fm3
....................uacccuguagauccgaauuGguu...	5	1	Fm3
....................uacccugCagauccgaauuuguu...	5	1	Fm3
....................uacccuguagauccgaauuuguG...	26	1	Fm3
....................Nacccuguagauccgaauuuguu...	19	1	Fm3
....................uacccuguagauccgaauuuAuu...	2	1	Fm3
....................Cacccuguagauccgaauuuguu...	8	1	Fm3
....................uacccuguaAauccgaauuuguu...	3	1	Fm3

```
                    Mature                                      Star
agaguucgcccaucagcagauacccuguagauccgaauuuguuugugaucgauaugacaaauuugaauuugcagcgggaucugaaguuggguauaaucaacugacaggagga
....................uaAccuguagauccgaauuuguu.....................................................................   11      1   Fm3
....................uacccGguagauccgaauuuguu.....................................................................   4       1   Fm3
....................uacAcuguagauccgaauuuguu.....................................................................   26      1   Fm3
....................uacccuAuagauccgaauuuguu.....................................................................   3       1   Fm3
....................uacccuguagauccgaauuugGu.....................................................................   1       1   Fm3
....................uacccuguagaucAgaauuuguu.....................................................................   17      1   Fm3
....................uacccuguaCauccgaauuuguu.....................................................................   1       1   Fm3
....................uacccuguagauccgaauuugCu.....................................................................   5       1   Fm3
....................uacccuCuagauccgaauuuguu.....................................................................   11      1   Fm3
....................uacccuguagauGcgaauuuguu.....................................................................   3       1   Fm3
....................uacccuguagauccgaauuuguA.....................................................................   13632   1   Fm3
....................uacccuguagauccgaauuugAu.....................................................................   13      1   Fm3
....................uacGcuguagauccgaauuuguu.....................................................................   1       1   Fm3
....................uacUcuguagauccgaauuuguu.....................................................................   6       1   Fm3
....................uacccuguagauccgaauAuguu.....................................................................   1       1   Fm3
....................uaccUuguagauccgaauuuguu.....................................................................   7       1   Fm3
....................uacccuguagauccgaaGuuguu.....................................................................   249     1   Fm3
....................uaGccuguagauccgaauuuguu.....................................................................   9       1   Fm3
....................uacccuguagauccgUauuuguu.....................................................................   10      1   Fm3
....................uacccuguagauccgaaCuuguu.....................................................................   292     1   Fm3
....................uacccuguagauAcgaauuuguu.....................................................................   4       1   Fm3
....................uacccuguGgauccgaauuuguu.....................................................................   11      1   Fm3
....................uaccAuguagauccgaauuuguu.....................................................................   22      1   Fm3
....................uacccuguagauccgaCuuuguu.....................................................................   100     1   Fm3
....................uacccAguagauccgaauuuguu.....................................................................   4       1   Fm3
....................uacccuguagauccgaaAuuguu.....................................................................   212     1   Fm3
....................uacccuguagauccgaauCuguu.....................................................................   11      1   Fm3
....................uacccuguagaGccgaauuuguu.....................................................................   3       1   Fm3
....................uacccuguagauccgaauuCguu.....................................................................   5       1   Fm3
....................uacccuguagauccAaauuuguu.....................................................................   8       1   Fm3
....................uCcccuguagauccgaauuuguu.....................................................................   1       1   Fm3
....................uacccuguCgauccgaauuuguu.....................................................................   1       1   Fm3
....................uacccuguagCuccgaauuuguu.....................................................................   43      1   Fm3
....................uaccGuguagauccgaauuuguu.....................................................................   8       1   Fm3
....................uGcccuguagauccgaauuuguu.....................................................................   10      1   Fm3
....................uacccuguUgauccgaauuuguu.....................................................................   10      1   Fm3
....................uacccuguagaAccgaauuuguu.....................................................................   5       1   Fm3
....................uacccuguagauccgaauuuguu.....................................................................   46028   0   Fm3
....................uacccNguagauccgaauuuguu.....................................................................   11      1   Fm3
....................uacccuguagauccgaGuuuguu.....................................................................   60      1   Fm3
....................uacccuguagGuccgaauuuguu.....................................................................   47      1   Fm3
....................uaUccuguagauccgaauuuguu.....................................................................   18      1   Fm3
....................uacccuguagaucUgaauuuguu.....................................................................   111     1   Fm3
....................Aacccuguagauccgaauuuguu.....................................................................   8       1   Fm3
....................uacccuguagauUcgaauuuguu.....................................................................   32      1   Fm3
....................uacccuguagauccgGauuuguu.....................................................................   4       1   Fm3
....................uUcccuguagauccgaauuuguu.....................................................................   7       1   Fm3
....................uNcccuguagauccgaauuuguu.....................................................................   1       1   Fm3
....................uacccuguagauccUaauuuguu.....................................................................   5       1   Fm3
....................uacccugAagauccgaauuuguu.....................................................................   6       1   Fm3
....................uacccuguagauccgCauuuguu.....................................................................   1       1   Fm3
....................uacccuguagauccgaauGuguu.....................................................................   1       1   Fm3
....................uacccuguagaucGgaauuuguu.....................................................................   5       1   Fm3
....................uacccuguagaCccgaauuuguu.....................................................................   8       1   Fm3
....................uacccCguagauccgaauuuguu.....................................................................   4       1   Fm3
....................uacccuguagUuccgaauuuguu.....................................................................   37      1   Fm3
....................uacccuguagauccgaauuuguC.....................................................................   31      1   Fm3
....................uacccuUuagauccgaauuuguu.....................................................................   5       1   Fm3
....................uacccuguagauccgaUuuuguu.....................................................................   73      1   Fm3
....................uacccuguaUauccgaauuuguu.....................................................................   7       1   Fm3
....................uacccuguagauccgaaCuuguuu....................................................................   11      1   Fm3
....................uacccuguagauccgaauuuguGu....................................................................   17      1   Fm3
....................uacccuguagauccgaauuuguuG....................................................................   8       1   Fm3
....................uacccuguagaucUgaauuuguuu....................................................................   8       1   Fm3
....................uacccuguagauccgaCuuuguuu....................................................................   5       1   Fm3
....................uacccuguagGuccgaauuuguuu....................................................................   1       1   Fm3
....................uacUcuguagauccgaauuuguuu....................................................................   1       1   Fm3
....................uacccuguagaucAgaauuuguuu....................................................................   1       1   Fm3
....................uacccuguagauccgaUuuuguuu....................................................................   2       1   Fm3
....................uaccAuguagauccgaauuuguuu....................................................................   2       1   Fm3
```

```
                    Mature                                Star
agaguucgcccaucagcagauacccuguagauccgaauuuguuugugaucgauaugacaaauuugaauuugcagcgggaucugaaguuggguauaaucaacugacaggagga
....................uacccuguagauccgaauuugAuu....................................................................	1	1	Fm3
....................uacccuguagauUcgaauuuguuu....................................................................	2	1	Fm3
....................uacccuguagauccgaauuuguuu....................................................................	998	0	Fm3
....................uacccuguagauccgaaAuuguuu....................................................................	2	1	Fm3
....................uaUccuguagauccgaauuuguuu....................................................................	1	1	Fm3
....................uacccuNuagauccgaauuuguuu....................................................................	1	1	Fm3
....................Nacccuguagauccgaauuuguuu....................................................................	1	1	Fm3
....................uacccuguagauccgaauuuguuC....................................................................	4	1	Fm3
....................uacccuguagUuccgaauuuguuu....................................................................	1	1	Fm3
....................uacccuguGgauccgaauuuguuu....................................................................	1	1	Fm3
....................uacccuguagauccgaaGuuguuu....................................................................	6	1	Fm3
....................uacccuguagauccgaauuuguCu....................................................................	44	1	Fm3
....................uacccuguagauccgaauuuguAu....................................................................	1626	1	Fm3
....................uacccuguagauccgaGuuuguuu....................................................................	1	1	Fm3
....................uacccuguagauccgaauuuguuA....................................................................	239	1	Fm3
....................uacccuguagauccgaauuuguuug...................................................................	1	0	Fm3
....................uacccuguagauccgaauuuguuuA...................................................................	1	1	Fm3
....................uacccuguagauccgaauuuguuuU...................................................................	8	1	Fm3
....................uacccuguagauccgaauuuUuuugu..................................................................	1	1	Fm3
....................uacccuguagauccgaauuuguuugu..................................................................	24	0	Fm3
....................uacccuguagauccgaaCuuguuugu..................................................................	1	1	Fm3
....................uacccuguagauccgaauuuguuuUu..................................................................	1	1	Fm3
....................uacccuguagauccgaauuuguuugug.................................................................	2	0	Fm3
....................uacccuguagauccgaauuuguuuguga................................................................	3	0	Fm3
....................uacccuguagauccgaauuuguuugugU................................................................	2	1	Fm3
....................uacccuguagauccgaauuuguuugugau...............................................................	2	0	Fm3
.....................acccuguagauccgaauA.........................................................................	5	1	Fm3
.....................acccuguagauccgaaAu.........................................................................	1	1	Fm3
.....................acccuguagCuccgaauu.........................................................................	1	1	Fm3
.....................acccuguagauccgaCuu.........................................................................	1	1	Fm3
.....................acccuguagauccgaauu.........................................................................	84	0	Fm3
.....................acccuguagaucAgaauuu........................................................................	1	1	Fm3
.....................acccuguagauccgaauuu........................................................................	108	0	Fm3
.....................acccuguagauccgaauGu........................................................................	1	1	Fm3
.....................acccuguagauccgaauAu........................................................................	6	1	Fm3
.....................acccuguagaucUgaauuu........................................................................	1	1	Fm3
.....................acccCguagauccgaauuug.......................................................................	1	1	Fm3
.....................acccuguagauccgaauCug.......................................................................	1	1	Fm3
.....................acccuguGgauccgaauuug.......................................................................	1	1	Fm3
.....................acccuguagauccgaauuuU.......................................................................	1	1	Fm3
.....................acccuguagaucAgaauuug.......................................................................	1	1	Fm3
.....................acccuguagauccgaauuCg.......................................................................	1	1	Fm3
.....................acccuguaAauccgaauuug.......................................................................	2	1	Fm3
.....................acccuguCgauccgaauuug.......................................................................	1	1	Fm3
.....................acccuguagaucUgaauuug.......................................................................	1	1	Fm3
.....................acccuguagauccgaCuuug.......................................................................	1	1	Fm3
.....................acccuguagauccgaauuug.......................................................................	330	0	Fm3
.....................acAcuguagauccgaauuug.......................................................................	1	1	Fm3
.....................acccuCuagauccgaauuug.......................................................................	2	1	Fm3
.....................acccuguagauccgaauuuA.......................................................................	1	1	Fm3
.....................acccugAagauccgaauuug.......................................................................	1	1	Fm3
.....................Ucccuguagauccgaauuug.......................................................................	1	1	Fm3
.....................acccuguagGuccgaauuug.......................................................................	1	1	Fm3
.....................acccugGagauccgaauuugu......................................................................	15	1	Fm3
.....................acccuguagauUcgaauuugu......................................................................	42	1	Fm3
.....................acccuguagGuccgaauuugu......................................................................	18	1	Fm3
.....................acccuguagauccgaauuuCu......................................................................	46	1	Fm3
.....................acccuguagaGccgaauuugu......................................................................	100	1	Fm3
.....................acccuguagCuccgaauuugu......................................................................	36	1	Fm3
.....................acccuguagauccgaaGuugu......................................................................	29	1	Fm3
.....................acccuguagauccgaauuugC......................................................................	8	1	Fm3
.....................acccuguagauccgaauuuAu......................................................................	26	1	Fm3
.....................accGuguagauccgaauuugu......................................................................	7	1	Fm3
.....................acccuguagauccgaauuuUu......................................................................	64	1	Fm3
.....................aAccuguagauccgaauuugu......................................................................	30	1	Fm3
.....................acccugCagauccgaauuugu......................................................................	45	1	Fm3
.....................acccugNagauccgaauuugu......................................................................	3	1	Fm3
.....................acccuguagUuccgaauuugu......................................................................	24	1	Fm3
.....................acccuguagauccgaauuugu......................................................................	36410	0	Fm3
.....................acccuguagauccgUauuugu......................................................................	27	1	Fm3
```

```
                    Mature                               Star
agaguucgcccaucagcagauacccuguagauccgaauuuguuugugaucgauaugacaaauuugaauuugcagcgggaucugaaguuggguauaaucaacugacaggagga
.....................acccuguagauccgCauuugu......................................................................    59    1    Fm3
.....................acccuguagauccAaauuugu......................................................................    17    1    Fm3
.....................acccuguagauccgaCuuugu......................................................................    89    1    Fm3
.....................acccuguagauccgaUuuugu......................................................................    25    1    Fm3
.....................acccuguagauccgaauuCgu......................................................................    14    1    Fm3
.....................acccuguagaAccgaauuugu......................................................................    96    1    Fm3
.....................acccuguagauccgGauuugu......................................................................    22    1    Fm3
.....................acccuguagauccgaauuAgu......................................................................    19    1    Fm3
.....................acccuguagauccgaauAugu......................................................................    100   1    Fm3
.....................acccuguagauccgaaAuugu......................................................................    20    1    Fm3
.....................acccuguagauccCaauuugu......................................................................    2     1    Fm3
.....................acUcuguagauccgaauuugu......................................................................    13    1    Fm3
.....................acccuguGgauccgaauuugu......................................................................    7     1    Fm3
.....................acccuguUgauccgaauuugu......................................................................    13    1    Fm3
.....................acAcuguagauccgaauuugu......................................................................    33    1    Fm3
.....................acccuguagaCccgaauuugu......................................................................    45    1    Fm3
.....................acccuguagaucAgaauuugu......................................................................    37    1    Fm3
.....................accUuguagauccgaauuugu......................................................................    14    1    Fm3
.....................acccuguagauAcgaauuugu......................................................................    21    1    Fm3
.....................acccuguaAauccgaauuugu......................................................................    19    1    Fm3
.....................acccCguagauccgaauuugu......................................................................    21    1    Fm3
.....................Ucccuguagauccgaauuugu......................................................................    25    1    Fm3
.....................acccuguagauccgaauGugu......................................................................    113   1    Fm3
.....................acccuUuagauccgaauuugu......................................................................    15    1    Fm3
.....................acccuguagauccUaauuugu......................................................................    7     1    Fm3
.....................acccAguagauccgaauuugu......................................................................    12    1    Fm3
.....................acccuguaCauccgaauuugu......................................................................    27    1    Fm3
.....................acccuguagauccgaauCugu......................................................................    215   1    Fm3
.....................acccuCuagauccgaauuugu......................................................................    6     1    Fm3
.....................acccuguagauccgaauuGgu......................................................................    24    1    Fm3
.....................accAuguagauccgaauuugu......................................................................    38    1    Fm3
.....................acccuguagauccgaaCuugu......................................................................    17    1    Fm3
.....................Ncccuguagauccgaauuugu......................................................................    11    1    Fm3
.....................acccuguagaucGgaauuugu......................................................................    17    1    Fm3
.....................acccuguagauccgaGuuugu......................................................................    30    1    Fm3
.....................acccuguCgauccgaauuugu......................................................................    18    1    Fm3
.....................aUccuguagauccgaauuugu......................................................................    5     1    Fm3
.....................aGccuguagauccgaauuugu......................................................................    9     1    Fm3
.....................acccuNuagauccgaauuugu......................................................................    6     1    Fm3
.....................acccuguagauGcgaauuugu......................................................................    3     1    Fm3
.....................acccuguaUauccgaauuugu......................................................................    37    1    Fm3
.....................acccuguagauccgaauuugG......................................................................    4     1    Fm3
.....................acccuguagauccgaauuugA......................................................................    20    1    Fm3
.....................acGcuguagauccgaauuugu......................................................................    9     1    Fm3
.....................acccuguagaucUgaauuugu......................................................................    46    1    Fm3
.....................acccuAuagauccgaauuugu......................................................................    16    1    Fm3
.....................Gcccuguagauccgaauuugu......................................................................    7     1    Fm3
.....................acccugAagauccgaauuugu......................................................................    22    1    Fm3
.....................acccGguagauccgaauuugu......................................................................    9     1    Fm3
.....................acccuguagauccgaauGuguu.....................................................................    1     1    Fm3
.....................acccuguagauccgaaCuuguu.....................................................................    1     1    Fm3
.....................acccuguagauccgaaGuuguu.....................................................................    2     1    Fm3
.....................Ucccuguagauccgaauuuguu.....................................................................    2     1    Fm3
.....................acccuguagaAccgaauuuguu.....................................................................    1     1    Fm3
.....................acccuguaAauccgaauuuguu.....................................................................    1     1    Fm3
.....................acccuguagauccgaauAuguu.....................................................................    1     1    Fm3
.....................acccugGagauccgaauuuguu.....................................................................    1     1    Fm3
.....................acccuguCgauccgaauuuguu.....................................................................    1     1    Fm3
.....................acccuNuagauccgaauuuguu.....................................................................    1     1    Fm3
.....................accGuguagauccgaauuuguu.....................................................................    1     1    Fm3
.....................acccuguagUuccgaauuuguu.....................................................................    1     1    Fm3
.....................acccugCagauccgaauuuguu.....................................................................    1     1    Fm3
.....................acccuguagauccgaaAuuguu.....................................................................    1     1    Fm3
.....................acccuAuagauccgaauuuguu.....................................................................    1     1    Fm3
.....................acccuguagGuccgaauuuguu.....................................................................    1     1    Fm3
.....................aAccuguagauccgaauuuguu.....................................................................    2     1    Fm3
.....................acccuguagaucAgaauuuguu.....................................................................    3     1    Fm3
.....................acccuguagauccgaauuAguu.....................................................................    1     1    Fm3
.....................acccuguagauccgaauCuguu.....................................................................    1     1    Fm3
.....................acccuguagauccgaauuuguA.....................................................................    100   1    Fm3
```

```
                    Mature                                Star
agaguucgcccaucagcagauacccuguagauccgaauuuguuugugaucgauaugacaaauuugaauuugcagcgggaucugaaguuggguauaaucaacugacaggagga
.....................acccuguagaGccgaauuuguu.....................................................................    1      1      Fm3
.....................accAuguagauccgaauuuguu.....................................................................    1      1      Fm3
.....................acccuguagaCccgaauuuguu.....................................................................    2      1      Fm3
.....................Gcccuguagauccgaauuuguu.....................................................................    1      1      Fm3
.....................acccuguagaucUgaauuuguu.....................................................................    1      1      Fm3
.....................acccuguagauccgaauuuguu.....................................................................    530    0      Fm3
.....................acccuguagauccgaauuuUuu.....................................................................    3      1      Fm3
.....................acccuguagauccgaauuuguCu....................................................................    1      1      Fm3
.....................acccuguagauccgaauuuguuA....................................................................    13     1      Fm3
.....................acccuguagauccgaauuuguuu....................................................................    14     0      Fm3
.....................acccuguagauccgaauuuguAu....................................................................    25     1      Fm3
.....................acccuguagauccgaauuugAuu....................................................................    1      1      Fm3
.....................acccuguagaucUgaauuuguuu....................................................................    2      1      Fm3
.....................acccuguagauccgaaAuuguuu....................................................................    1      1      Fm3
.....................acccuguagauccgaauuuguuugugau...............................................................    1      0      Fm3
......................cccuguagauccgGauuu........................................................................    1      1      Fm3
......................cccuguagauccgaauuu........................................................................    3      0      Fm3
......................cccuguagaucUgaauuu........................................................................    1      1      Fm3
......................cccuguagauccgaauuug.......................................................................    13     0      Fm3
......................cccuguagauAcgaauuug.......................................................................    1      1      Fm3
......................cccuguCgauccgaauuugu......................................................................    1      1      Fm3
......................cccuguagauccgaauuGgu......................................................................    11     1      Fm3
......................cccuguagauccgaauuugu......................................................................    1734   0      Fm3
......................cccuguagUuccgaauuugu......................................................................    24     1      Fm3
......................cccuguUgauccgaauuugu......................................................................    1      1      Fm3
......................cccuAuagauccgaauuugu......................................................................    2      1      Fm3
......................cccugCagauccgaauuugu......................................................................    1      1      Fm3
......................cccuguaAauccgaauuugu......................................................................    11     1      Fm3
......................cccuguagauccgaauuAgu......................................................................    2      1      Fm3
......................cccGguagauccgaauuugu......................................................................    1      1      Fm3
......................cccCguagauccgaauuugu......................................................................    2      1      Fm3
......................cccuguagCuccgaauuugu......................................................................    1      1      Fm3
......................Accuguagauccgaauuugu......................................................................    1      1      Fm3
......................ccUuguagauccgaauuugu......................................................................    3      1      Fm3
......................cccuguGgauccgaauuugu......................................................................    2      1      Fm3
......................cccuguagaAccgaauuugu......................................................................    1      1      Fm3
......................Ucccuguagauccgaauuugu.....................................................................    8      1      Fm3
......................cccuguagauccAaauuugu......................................................................    5      1      Fm3
......................cccuguagGuccgaauuugu......................................................................    1      1      Fm3
......................cccuguagauccgaauuugA......................................................................    1      1      Fm3
......................cccuguagauccgaUuuugu......................................................................    28     1      Fm3
......................cccuguagaucGgaauuugu......................................................................    2      1      Fm3
......................cccuguagauccgaauAugu......................................................................    1      1      Fm3
......................ccGuguagauccgaauuugu......................................................................    1      1      Fm3
......................cccuguagauccgaauuCgu......................................................................    9      1      Fm3
......................cccuguagauccgaauGugu......................................................................    1      1      Fm3
......................cccuguagauccgaauuuAu......................................................................    1      1      Fm3
......................cccugAagauccgaauuugu......................................................................    21     1      Fm3
......................cccuguagaCccgaauuugu......................................................................    2      1      Fm3
......................cccuguagauccUaauuugu......................................................................    1      1      Fm3
......................cccuguagauccgaauuuUu......................................................................    88     1      Fm3
......................cccuguagauAcgaauuugu......................................................................    2      1      Fm3
......................ccAuguagauccgaauuugu......................................................................    2      1      Fm3
......................Gccuguagauccgaauuugu......................................................................    1      1      Fm3
......................cAcuguagauccgaauuugu......................................................................    1      1      Fm3
......................cccuguagUuccgaauuuguu.....................................................................    1      1      Fm3
......................cccuguagauccgaauuuUuu.....................................................................    3      1      Fm3
......................cccuguagauccgaauuuguu.....................................................................    34     0      Fm3
......................cccuguagauccgaauuuguA.....................................................................    12     1      Fm3
......................cccuUuagauccgaauuuguu.....................................................................    1      1      Fm3
......................cccuguagauccgaauuuguuu....................................................................    2      0      Fm3
......................cccuguagauccgaauuuguuA....................................................................    1      1      Fm3
......................cccuguagauccgaauuuguAu....................................................................    1      1      Fm3
.......................ccuguagauccgGauuug.......................................................................    1      1      Fm3
.......................ccuguagauccgaauuug.......................................................................    1      0      Fm3
.......................Ucuguagauccgaauuugu......................................................................    2      1      Fm3
.......................ccuguagauccUaauuugu......................................................................    1      1      Fm3
.......................ccuguagauccgGauuugu......................................................................    2      1      Fm3
.......................ccuguagaAccgaauuugu......................................................................    2      1      Fm3
.......................ccuguagauccgaauuuUu......................................................................    1      1      Fm3
```

```
                    Mature                                Star
agaguucgcccaucagcagauacccuguagauccgaauuuguuugugaucgauaugacaaauuugaauuugcagcgggaucugaaguuggguauaaucaacugacaggagga
.......................ccuAuagauccgaauuugu......................................................................   1      1    Fm3
.......................ccuguagauccgaauuCgu......................................................................   2      1    Fm3
.......................ccuguagauUcgaauuugu......................................................................   1      1    Fm3
.......................ccuguagauccgaauuugu......................................................................   195    0    Fm3
.......................ccuguagauccgaauuuCu......................................................................   1      1    Fm3
.......................ccuguagauccgaauuuguu.....................................................................   4      0    Fm3
........................cuguagauccgaauuugu......................................................................   21     0    Fm3
........................cCguagauccgaauuugu......................................................................   1      1    Fm3
........................cuguagauccgaauuugA......................................................................   1      1    Fm3
.........................uguagauccgaauuuguu.....................................................................   2      0    Fm3
.........................................uuugugaucgauaugaca.....................................................   4      0    Fm3
...........................................ugugaucgauaugacaaauuugaauu...........................................   2      0    Fm3
..........................................................aaauuugaauuugcagcgggau................................   1      0    Fm3

..................Cauacccuguagauccgaauuugu......................................................................   2      1    Mm3
...................auacccuguagaAccgaa...........................................................................   7      1    Mm3
...................Cuacccuguagauccgaau..........................................................................   1      1    Mm3
...................auacccuguagauccgaau..........................................................................   3      0    Mm3
...................auacccuguagaAccgaau..........................................................................   5      1    Mm3
...................auacccuguagaAccgaauu.........................................................................   3      1    Mm3
...................Cuacccuguagauccgaauu.........................................................................   1      1    Mm3
...................auacccuguagauccgaauu.........................................................................   5      0    Mm3
...................Cuacccuguagauccgaauuu........................................................................   8      1    Mm3
...................auacccuguagauccgaauuu........................................................................   3      0    Mm3
...................auacccuguagaAccgaauuu........................................................................   3      1    Mm3
...................auacccuguagauccgaauuug.......................................................................   16     0    Mm3
...................Cuacccuguagauccgaauuug.......................................................................   5      1    Mm3
...................auacccuguagaAccgaauuug.......................................................................   2      1    Mm3
...................Cuacccuguagauccgaauuugu......................................................................   1156   1    Mm3
...................auacccCguagauccgaauuugu......................................................................   1      1    Mm3
...................auacccuguagauccgaaCuugu......................................................................   6      1    Mm3
...................auacccuguagaucAgaauuugu......................................................................   1      1    Mm3
...................Uuacccuguagauccgaauuugu......................................................................   2      1    Mm3
...................auacccuUuagauccgaauuugu......................................................................   1      1    Mm3
...................auacccuguagauccgaauuugu......................................................................   2153   0    Mm3
...................auacccuguagauccgUauuugu......................................................................   4      1    Mm3
...................auacAcuguagauccgaauuugu......................................................................   1      1    Mm3
...................auacccuguagauccgaauuugG......................................................................   5      1    Mm3
...................auacGcuguagauccgaauuugu......................................................................   1      1    Mm3
...................auacccuguaCauccgaauuugu......................................................................   2      1    Mm3
...................auacccuguagauccAaauuugu......................................................................   1      1    Mm3
...................auacccuguagauUcgaauuugu......................................................................   1      1    Mm3
...................auacccuAuagauccgaauuugu......................................................................   1      1    Mm3
...................auacccuguagaucUgaauuugu......................................................................   1      1    Mm3
...................auacccuguagauccgaGuuugu......................................................................   9      1    Mm3
...................aGacccuguagauccgaauuugu......................................................................   1      1    Mm3
...................Guacccuguagauccgaauuugu......................................................................   35     1    Mm3
...................auacccuguCgauccgaauuugu......................................................................   1      1    Mm3
...................auaccUuguagauccgaauuugu......................................................................   1      1    Mm3
...................auacccuguagaAccgaauuugu......................................................................   280    1    Mm3
...................auacccuguagauccgaCuuugu......................................................................   16     1    Mm3
...................auacccuguagauccgCauuugu......................................................................   7      1    Mm3
...................auacccuguagauccgaUuuugu......................................................................   5      1    Mm3
...................auacccuguagauccgaauuuguA.....................................................................   9      1    Mm3
...................auacccuguagaAccgaauuuguu.....................................................................   3      1    Mm3
...................Cuacccuguagauccgaauuuguu.....................................................................   9      1    Mm3
...................auacccuguaAauccgaauuuguu.....................................................................   1      1    Mm3
...................auacccuguagauccgaauuuguu.....................................................................   15     0    Mm3
...................auacccuguagauccgaGuuuguu.....................................................................   1      1    Mm3
...................Cuacccuguagauccgaauuuguuu....................................................................   2      1    Mm3
...................auacccuguagauccgaauuuguuu....................................................................   1      0    Mm3
....................uacccuguagauccgaau..........................................................................   2243   0    Mm3
....................uacccuguagauccgaaG..........................................................................   9      1    Mm3
....................uGcccuguagauccgaau..........................................................................   1      1    Mm3
....................uacccuguagauccUaau..........................................................................   1      1    Mm3
....................uacccuguagauccgaaA..........................................................................   66     1    Mm3
....................uacccAguagauccgaau..........................................................................   1      1    Mm3
....................uaUccuguagauccgaau..........................................................................   1      1    Mm3
....................uacccugAagauccgaau..........................................................................   1      1    Mm3
....................uacccuguagGuccgaau..........................................................................   3      1    Mm3
```

Mature Star

agaguucgcccaucagcagauacccuguagauccgaauuuguuugugaucgauaugacaaauuugaauuugcagcgggaucugaaguuggguauaaucaacugacaggagga			
....................uacccuguagauccgaGu..	4	1	Mm3
....................uacccuguagaAccgaau..	3	1	Mm3
....................uacccuguagauccgaUu..	3	1	Mm3
....................uacccuguagauccgaaC..	13	1	Mm3
....................uacUcuguagauccgaau..	1	1	Mm3
....................uacccuguagUuccgaau..	2	1	Mm3
....................uacccuguagaucUgaau..	3	1	Mm3
....................uacGcuguagauccgaau..	1	1	Mm3
....................uacccuguagauccgaCu..	4	1	Mm3
....................uacccuguagauccgUau..	1	1	Mm3
....................uUcccuguagauccgaau..	1	1	Mm3
....................uaGccuguagauccgaau..	1	1	Mm3
....................uacAcuguagauccgaau..	1	1	Mm3
....................uacccuguagaCccgaau..	1	1	Mm3
....................uaccAuguagauccgaau..	1	1	Mm3
....................uacccuguaCauccgaau..	2	1	Mm3
....................uaAccuguagauccgaau..	1	1	Mm3
....................uacccuguagCuccgaau..	2	1	Mm3
....................uacccuguagauUcgaau..	3	1	Mm3
....................uacccuguagCuccgaauu...	3	1	Mm3
....................uacccuUuagauccgaauu...	1	1	Mm3
....................uacccuguagauccgaaGu...	16	1	Mm3
....................uacccuguagauccgaUuu...	3	1	Mm3
....................uUcccuguagauccgaauu...	1	1	Mm3
....................uacccuguagauUcgaauu...	2	1	Mm3
....................uacccuguagaucUgaauu...	4	1	Mm3
....................uacccuguagauccgaCuu...	9	1	Mm3
....................uacccuguagauccgUauu...	1	1	Mm3
....................uacUcuguagauccgaauu...	2	1	Mm3
....................uacccuguagauccgaaCu...	24	1	Mm3
....................uacccuguUgauccgaauu...	1	1	Mm3
....................uaAccuguagauccgaauu...	1	1	Mm3
....................uacccuguagGuccgaauu...	5	1	Mm3
....................uacccuguagauccgaauA...	117	1	Mm3
....................uacccuNuagauccgaauu...	1	1	Mm3
....................uacccuguagauccgGauu...	2	1	Mm3
....................uacccuguagauccgaauG...	3	1	Mm3
....................uacccuguagaCccgaauu...	1	1	Mm3
....................uCcccuguagauccgaauu...	3	1	Mm3
....................Aacccuguagauccgaauu...	1	1	Mm3
....................uacAcuguagauccgaauu...	2	1	Mm3
....................uacccuguagauccgaaAu...	42	1	Mm3
....................uacccuguagauccgaauu...	4112	0	Mm3
....................uaccUuguagauccgaauu...	1	1	Mm3
....................Cacccuguagauccgaauu...	1	1	Mm3
....................Nacccuguagauccgaauu...	4	1	Mm3
....................uacccuguagUuccgaauu...	4	1	Mm3
....................uacccuguagauccgaGuu...	7	1	Mm3
....................uGcccuguagauccgaauu...	1	1	Mm3
....................uacccugCagauccgaauu...	2	1	Mm3
....................uaUccuguagauccgaauu...	1	1	Mm3
....................uacccuguagaAccgaauuu..	6	1	Mm3
....................uacccuguagauccgaGuuu..	10	1	Mm3
....................Cacccuguagauccgaauuu..	3	1	Mm3
....................uacccGguagauccgaauuu..	1	1	Mm3
....................uacccAguagauccgaauuu..	2	1	Mm3
....................uacccuguagauccgaaAuu..	47	1	Mm3
....................uaccGuguagauccgaauuu..	1	1	Mm3
....................uacccuguagauccgCauuu..	2	1	Mm3
....................uacccuguagauUcgaauuu..	8	1	Mm3
....................uacccuguagaucUgaauuu..	8	1	Mm3
....................uacccuguaUauccgaauuu..	1	1	Mm3
....................uacccuguagauccgaauuG..	2	1	Mm3
....................uacccuguagauccgaaCuu..	49	1	Mm3
....................uacccuguagauccgaaGuu..	52	1	Mm3
....................uacccuguagauccgaUuuu..	17	1	Mm3
....................uacccuguagGuccgaauuu..	5	1	Mm3
....................uacccuAuagauccgaauuu..	2	1	Mm3
....................uacccuUuagauccgaauuu..	4	1	Mm3
....................uacccuguaAauccgaauuu..	1	1	Mm3

```
                    Mature                              Star
agaguucgcccaucagcagauacccuguagauccgaauuuguuugugaucgauaugacaaauuugaauuugcagcgggaucugaaguuggguauaaucaacugacaggagga
....................uacccuguagauccgaauAu.......................................................................   20      1   Mm3
....................uacccuguagauccgaauuA.......................................................................   50      1   Mm3
....................uacGcuguagauccgaauuu.......................................................................   1       1   Mm3
....................uacccuguagaucGgaauuu.......................................................................   2       1   Mm3
....................uacccuguagauccgaauuC.......................................................................   2       1   Mm3
....................uaAccuguagauccgaauuu.......................................................................   1       1   Mm3
....................uacccuguagUuccgaauuu.......................................................................   6       1   Mm3
....................uaccUuguagauccgaauuu.......................................................................   3       1   Mm3
....................uacccuguagauccAaauuu.......................................................................   1       1   Mm3
....................uaUccuguagauccgaauuu.......................................................................   1       1   Mm3
....................uacccuguagCuccgaauuu.......................................................................   8       1   Mm3
....................uacccuguUgauccgaauuu.......................................................................   1       1   Mm3
....................uacccuguagaucAgaauuu.......................................................................   1       1   Mm3
....................Aacccuguagauccgaauuu.......................................................................   4       1   Mm3
....................Nacccuguagauccgaauuu.......................................................................   1       1   Mm3
....................uacccuguagauccgaauuu.......................................................................   9025    0   Mm3
....................uacccuguagauAcgaauuu.......................................................................   1       1   Mm3
....................uGcccuguagauccgaauuu.......................................................................   2       1   Mm3
....................uacccuNuagauccgaauuu.......................................................................   1       1   Mm3
....................uacAcuguagauccgaauuu.......................................................................   5       1   Mm3
....................uacccuguagauccgaCuuu.......................................................................   20      1   Mm3
....................uacccuguaAauccgaauuug......................................................................   1       1   Mm3
....................uacccuguagauccgaauuug......................................................................   21600   0   Mm3
....................Aacccuguagauccgaauuug......................................................................   9       1   Mm3
....................uacccuguagauccgaaGuug......................................................................   112     1   Mm3
....................Nacccuguagauccgaauuug......................................................................   8       1   Mm3
....................uacccuguagauccgGauuug......................................................................   3       1   Mm3
....................uacccuguagauUcgaauuug......................................................................   5       1   Mm3
....................uacccuNuagauccgaauuug......................................................................   1       1   Mm3
....................uacccuguagauccgaauuAg......................................................................   1       1   Mm3
....................uacccuguagCuccgaauuug......................................................................   26      1   Mm3
....................uacccuguUgauccgaauuug......................................................................   3       1   Mm3
....................uaAccuguagauccgaauuug......................................................................   2       1   Mm3
....................uaccGuguagauccgaauuug......................................................................   2       1   Mm3
....................uacccuguagauccgaGuuug......................................................................   32      1   Mm3
....................uacccAguagauccgaauuug......................................................................   1       1   Mm3
....................uacccuguagauccgaCuuug......................................................................   67      1   Mm3
....................uacccuguagauGcgaauuug......................................................................   1       1   Mm3
....................uacccuguagauccgaUuuug......................................................................   27      1   Mm3
....................uaGccuguagauccgaauuug......................................................................   4       1   Mm3
....................uacccuguagaucUgaauuug......................................................................   7       1   Mm3
....................uacccuguagGuccgaauuug......................................................................   30      1   Mm3
....................uaccUuguagauccgaauuug......................................................................   5       1   Mm3
....................uacccuguCgauccgaauuug......................................................................   3       1   Mm3
....................uacccuguGgauccgaauuug......................................................................   2       1   Mm3
....................uacccuguagauccgCauuug......................................................................   4       1   Mm3
....................Cacccuguagauccgaauuug......................................................................   4       1   Mm3
....................uacccuguagaAccgaauuug......................................................................   10      1   Mm3
....................uacccCguagauccgaauuug......................................................................   2       1   Mm3
....................uacccuguagauccgaaCuug......................................................................   151     1   Mm3
....................uacccuguagauccgUauuug......................................................................   3       1   Mm3
....................uacccuguagauccgaauuuA......................................................................   164     1   Mm3
....................uacccuguagauccgaauuCg......................................................................   2       1   Mm3
....................uacccNguagauccgaauuug......................................................................   2       1   Mm3
....................uacccuguagauccgaauuuU......................................................................   131     1   Mm3
....................uacccuguagaCccgaauuug......................................................................   1       1   Mm3
....................uGcccuguagauccgaauuug......................................................................   5       1   Mm3
....................uacccuguagauccAaauuug......................................................................   5       1   Mm3
....................uacccuguagauccgaauuuC......................................................................   3       1   Mm3
....................uacAcuguagauccgaauuug......................................................................   10      1   Mm3
....................uUcccuguagauccgaauuug......................................................................   5       1   Mm3
....................uCcccuguagauccgaauuug......................................................................   1       1   Mm3
....................uacccuguaUauccgaauuug......................................................................   1       1   Mm3
....................uacccuguagauccgaauuGg......................................................................   3       1   Mm3
....................uaccAuguagauccgaauuug......................................................................   2       1   Mm3
....................uacccuguagauccgaauCug......................................................................   7       1   Mm3
....................uacccuguagauccgaaAuug......................................................................   107     1   Mm3
....................uacccugAagauccgaauuug......................................................................   1       1   Mm3
....................uacccuguagUuccgaauuug......................................................................   25      1   Mm3
....................uacccugCagauccgaauuug......................................................................   2       1   Mm3
```

Mature Star

agaguucgcccaucagcagauacccuguagauccgaauuuguuugugaucgauaugacaaauuugaauuugcagcgggaucugaaguuggguauaaucaacugacaggagga			
....................uacccuguagauccUaauuug...	1	1	Mm3
....................uaUccuguagauccgaauuug...	1	1	Mm3
....................uacccuguagaucAgaauuug...	4	1	Mm3
....................uacccuguagauccCaauuug...	3	1	Mm3
....................uacccugGagauccgaauuug...	1	1	Mm3
....................uacUcuguagauccgaauuug...	6	1	Mm3
....................uacccuguagaGccgaauuug...	1	1	Mm3
....................uacccuAuagauccgaauuugu..	190	1	Mm3
....................uacccugGagauccgaauuugu..	53	1	Mm3
....................uacccuguagauccgaauAugu..	83	1	Mm3
....................uacccuguagauccgaauuugC..	1113	1	Mm3
....................uUccuguagauccgaauuugu..	501	1	Mm3
....................uacccuguagauccgaauuuAu..	225	1	Mm3
....................uacccuguagauccgaaAuugu..	11725	1	Mm3
....................uaGccuguagauccgaauuugu..	524	1	Mm3
....................uacccugAagauccgaauuugu..	223	1	Mm3
....................uacccuguUgauccgaauuugu..	629	1	Mm3
....................uacccugCagauccgaauuugu..	282	1	Mm3
....................uacccuguagauccgaaCuugu..	15844	1	Mm3
....................uacAcuguagauccgaauuugu..	943	1	Mm3
....................uacccuguagaCccgaauuugu..	278	1	Mm3
....................uacccNguagauccgaauuugu..	376	1	Mm3
....................uacccuguagauccgGauuugu..	293	1	Mm3
....................uacccuguagauccgUauuugu..	775	1	Mm3
....................Cacccuguagauccgaauuugu..	396	1	Mm3
....................uacccuguagCuccgaauuugu..	2366	1	Mm3
....................uacccuguagauccAaauuugu..	282	1	Mm3
....................uacccuguagauccgaauuCgu..	254	1	Mm3
....................uacccuguagauccgaauuugA..	1982	1	Mm3
....................uacccuguagGuccgaauuugu..	2600	1	Mm3
....................uacccuguagauccgaNuuugu..	6	1	Mm3
....................uacccuguagauccgaauCugu..	225	1	Mm3
....................uaccGuguagauccgaauuugu..	168	1	Mm3
....................uacccuguagauccgaaNuugu..	3	1	Mm3
....................Nacccuguagauccgaauuugu..	1213	1	Mm3
....................uacccuguaUauccgaauuugu..	180	1	Mm3
....................uCcccuguagauccgaauuugu..	149	1	Mm3
....................uacccuUuagauccgaauuugu..	175	1	Mm3
....................uacccuguagUuccgaauuugu..	2046	1	Mm3
....................uNcccuguagauccgaauuugu..	18	1	Mm3
....................uacccuguagauccgaauuuUu..	187	1	Mm3
....................uacccuguagauccgaauGugu..	55	1	Mm3
....................uacccCguagauccgaauuugu..	293	1	Mm3
....................uacccuguagauccgaauuugG..	3109	1	Mm3
....................Aacccuguagauccgaauuugu..	729	1	Mm3
....................uacccuguagauccgaauuGgu..	76	1	Mm3
....................uacccuguagauUcgaauuugu..	830	1	Mm3
....................uaNccuguagauccgaauuugu..	11	1	Mm3
....................uacccAguagauccgaauuugu..	153	1	Mm3
....................uacccuguagauccgaauuugu..	2516388	0	Mm3
....................uacccuguagauccgaUuuugu..	4069	1	Mm3
....................uacccuguagauccCaauuugu..	99	1	Mm3
....................uacGcuguagauccgaauuugu..	194	1	Mm3
....................uacccuguagaAccgaauuugu..	744	1	Mm3
....................uacccuguagauccUaauuugu..	170	1	Mm3
....................uacccuguagaucAgaauuugu..	513	1	Mm3
....................uacccGguagauccgaauuugu..	114	1	Mm3
....................uaUccuguagauccgaauuugu..	505	1	Mm3
....................uacccuguagauccgaauNugu..	5	1	Mm3
....................uacccuguagauccgaauuuCu..	68	1	Mm3
....................uacccuguagauccgCauuugu..	208	1	Mm3
....................uacccuguagauGcgaauuugu..	59	1	Mm3
....................uacccuguagaucGgaauuugu..	144	1	Mm3
....................uGcccuguagauccgaauuugu..	587	1	Mm3
....................uaAccuguagauccgaauuugu..	562	1	Mm3
....................uacccuguagaGccgaauuugu..	59	1	Mm3
....................uacccuguagauccgaCuuugu..	6473	1	Mm3
....................uacccuguagauccgaauuAgu..	170	1	Mm3
....................uacccuguaAauccgaauuugu..	133	1	Mm3
....................uacccuguagauccgaGuuugu..	4970	1	Mm3

```
                    Mature                                Star
agaguucgcccaucagcagauacccuguagauccgaauuuguuugugaucgauaugacaaauuugaauuugcagcgggaucugaaguuggguauaaucaacugacaggagga
....................uaccAuguagauccgaauuugu......................................................................   305     1   Mm3
....................uacccuguGgauccgaauuugu......................................................................   373     1   Mm3
....................uaccUuguagauccgaauuugu......................................................................   400     1   Mm3
....................uacccuguaCauccgaauuugu......................................................................   76      1   Mm3
....................uacccuNuagauccgaauuugu......................................................................   146     1   Mm3
....................uacccuguagauccgaaGuugu......................................................................   13209   1   Mm3
....................uacccuguCgauccgaauuugu......................................................................   105     1   Mm3
....................uacccuguagaucUgaauuugu......................................................................   1296    1   Mm3
....................uacccuCuagauccgaauuugu......................................................................   56      1   Mm3
....................uacccuguagauAcgaauuugu......................................................................   97      1   Mm3
....................uacUcuguagauccgaauuugu......................................................................   563     1   Mm3
....................Gacccuguagauccgaauuugu......................................................................   61      1   Mm3
....................uacccuguagauccgUauuuguu.....................................................................   17      1   Mm3
....................uNcccuguagauccgaauuuguu.....................................................................   2       1   Mm3
....................uacccuguagauccgaauuuguG.....................................................................   62      1   Mm3
....................Cacccuguagauccgaauuuguu.....................................................................   5       1   Mm3
....................uacccuguagauccgGauuuguu.....................................................................   3       1   Mm3
....................uaccUuguagauccgaauuuguu.....................................................................   3       1   Mm3
....................uacccuguagauccgaauuuguC.....................................................................   31      1   Mm3
....................uacccuguCgauccgaauuuguu.....................................................................   2       1   Mm3
....................uacccuguagaAccgaauuuguu.....................................................................   9       1   Mm3
....................uacccuguagauccgaauGuguu.....................................................................   1       1   Mm3
....................uacccuguagauccUaauuuguu.....................................................................   8       1   Mm3
....................uacccuguagaucAgaauuuguu.....................................................................   8       1   Mm3
....................uacccugAagauccgaauuuguu.....................................................................   3       1   Mm3
....................uacccNguagauccgaauuuguu.....................................................................   2       1   Mm3
....................uacccuguagauccgaauuugGu.....................................................................   1       1   Mm3
....................uacccuguagauccgaaGuuguu.....................................................................   148     1   Mm3
....................uacccuguagauccgaaCuuguu.....................................................................   173     1   Mm3
....................Aacccuguagauccgaauuuguu.....................................................................   7       1   Mm3
....................uacccuguagauUcgaauuuguu.....................................................................   6       1   Mm3
....................uacGcuguagauccgaauuuguu.....................................................................   3       1   Mm3
....................uacccuCuagauccgaauuuguu.....................................................................   2       1   Mm3
....................uacccuguagauccgaauAuguu.....................................................................   2       1   Mm3
....................uacUcuguagauccgaauuuguu.....................................................................   7       1   Mm3
....................uaAccuguagauccgaauuuguu.....................................................................   5       1   Mm3
....................uacccuguagaCccgaauuuguu.....................................................................   6       1   Mm3
....................uacccuguagGuccgaauuuguu.....................................................................   30      1   Mm3
....................uacccugCagauccgaauuuguu.....................................................................   1       1   Mm3
....................uacccuguagauccgaauuuAuu.....................................................................   5       1   Mm3
....................uacccuguagaucUgaauuuguu.....................................................................   36      1   Mm3
....................uacccuguUgauccgaauuuguu.....................................................................   5       1   Mm3
....................uacccuguagCuccgaauuuguu.....................................................................   31      1   Mm3
....................uacccuguagauGcgaauuuguu.....................................................................   2       1   Mm3
....................uacccuguagauccgaauuGguu.....................................................................   1       1   Mm3
....................Nacccuguagauccgaauuuguu.....................................................................   13      1   Mm3
....................uacccuguagauccgaauuugCu.....................................................................   5       1   Mm3
....................uacccuguagauccgaGuuuguu.....................................................................   54      1   Mm3
....................uUcccuguagauccgaauuuguu.....................................................................   6       1   Mm3
....................uacccuguagauccgaUuuuguu.....................................................................   46      1   Mm3
....................uacccuguagauccgaauuugAu.....................................................................   6       1   Mm3
....................uacccuguGgauccgaauuuguu.....................................................................   2       1   Mm3
....................uacccuguagauccgaauCuguu.....................................................................   2       1   Mm3
....................uCcccuguagauccgaauuuguu.....................................................................   2       1   Mm3
....................uacccuguagauccgaauuuguA.....................................................................   12603   1   Mm3
....................uacccuguagUuccgaauuuguu.....................................................................   25      1   Mm3
....................uGcccuguagauccgaauuuguu.....................................................................   5       1   Mm3
....................uacccCguagauccgaauuuguu.....................................................................   3       1   Mm3
....................uacccuUuagauccgaauuuguu.....................................................................   1       1   Mm3
....................uacccuguagauccgaauuCguu.....................................................................   1       1   Mm3
....................uacAcuguagauccgaauuuguu.....................................................................   11      1   Mm3
....................uaccGuguagauccgaauuuguu.....................................................................   2       1   Mm3
....................uaGccuguagauccgaauuuguu.....................................................................   6       1   Mm3
....................uacccuguaUauccgaauuuguu.....................................................................   5       1   Mm3
....................uacccuNuagauccgaauuuguu.....................................................................   2       1   Mm3
....................uacccuguagauccgaauuuguu.....................................................................   26666   0   Mm3
....................uaUccuguagauccgaauuuguu.....................................................................   4       1   Mm3
....................uaccAuguagauccgaauuuguu.....................................................................   5       1   Mm3
....................uacccuguagauccAaauuuguu.....................................................................   3       1   Mm3
....................Gacccuguagauccgaauuuguu.....................................................................   1       1   Mm3
```

```
                    Mature                                    Star
agaguucgcccaucagcagauacccuguagauccgaauuuguuugugaucgauaugacaaauuugaauuugcagcgggaucugaaguuggguauaaucaacugacaggagga
....................uacccuAuagauccgaauuuguu..................................................................... 1     1  Mm3
....................uacccuguagauccgaCuuuguu..................................................................... 67    1  Mm3
....................uacccuguagauccgaauuuUuu..................................................................... 4     1  Mm3
....................uacccuguagauccgaaAuuguu..................................................................... 116   1  Mm3
....................uacccuguagauccgaauuuguuC.................................................................... 1     1  Mm3
....................uacccuguagauccgaGuuuguuu.................................................................... 2     1  Mm3
....................uacccuguagauccgaUuuuguuu.................................................................... 3     1  Mm3
....................uacccuguagauccgaauuuguGu.................................................................... 7     1  Mm3
....................uacccuguagauccgaauuuguuA.................................................................... 294   1  Mm3
....................uacccuguagaucUgaauuuguuu.................................................................... 4     1  Mm3
....................Nacccuguagauccgaauuuguuu.................................................................... 1     1  Mm3
....................uacccuguagauccgaauuuguuu.................................................................... 664   0  Mm3
....................uacccuguagauccgaauuGguuu.................................................................... 1     1  Mm3
....................uacccuguagaAccgaauuuguuu.................................................................... 1     1  Mm3
....................uGcccuguagauccgaauuuguuu.................................................................... 1     1  Mm3
....................uacccuguagauccgaauuuguuG.................................................................... 9     1  Mm3
....................uacccuguagauccgaauuuguAu.................................................................... 1350  1  Mm3
....................uacccuguagauccgaaGuuguuu.................................................................... 2     1  Mm3
....................uacccuguagauccgaCuuuguuu.................................................................... 2     1  Mm3
....................uacAcuguagauccgaauuuguuu.................................................................... 1     1  Mm3
....................uacccuguagauccgaauuuguCu.................................................................... 14    1  Mm3
....................uacccuguagauccgaaCuuguuu.................................................................... 6     1  Mm3
....................uacccuguagauccgaaAuuguuu.................................................................... 7     1  Mm3
....................uacccuguagauccgaauuuguuug................................................................... 7     0  Mm3
....................uacccuguagauccgaauuuguuuA................................................................... 1     1  Mm3
....................uacccuguagauccgaaGuuguuugu.................................................................. 1     1  Mm3
....................uacccuguagauccgaauuuguuugu.................................................................. 20    0  Mm3
....................uacccuguagauccgaaAuuguuugu.................................................................. 1     1  Mm3
....................uacccuguagauccgaauuuguuuUu.................................................................. 1     1  Mm3
....................uacccuguagGuccgaauuuguuugug................................................................. 1     1  Mm3
....................uacccuguagauccgaauuuguuugug................................................................. 24    0  Mm3
....................uacccuguagauccgaauuuguuugugG................................................................ 1     1  Mm3
....................uacccuguagauccgaauuuguuugugU................................................................ 1     1  Mm3
....................uacccuguagauccgaauuuguuuguga................................................................ 1     0  Mm3
....................uacccuguagauccgaauuuguuugugUu............................................................... 1     1  Mm3
....................uacccuguagauccgaUuuuguuugugau............................................................... 1     1  Mm3
....................uacccuguagauccgaauuuguuugugau............................................................... 20    0  Mm3
.....................acccuguagauccgaauA......................................................................... 3     1  Mm3
.....................acccuguagauccgaauu......................................................................... 15    0  Mm3
.....................accUuguagauccgaauuu........................................................................ 2     1  Mm3
.....................acccuguagauccgaauCu........................................................................ 1     1  Mm3
.....................acccugGagauccgaauuu........................................................................ 2     1  Mm3
.....................acccuguagauccgaauuu........................................................................ 46    0  Mm3
.....................acccuguagauccgaaCuu........................................................................ 1     1  Mm3
.....................acGcuguagauccgaauuu........................................................................ 1     1  Mm3
.....................acccuguagauccgaauuuU....................................................................... 1     1  Mm3
.....................acccuguagaGccgaauuug....................................................................... 1     1  Mm3
.....................acccuguagauccgaauuCg....................................................................... 1     1  Mm3
.....................acccuguagauccgaauAug....................................................................... 1     1  Mm3
.....................acccuguagauccgaauCug....................................................................... 2     1  Mm3
.....................acccuguagauccgaauuug....................................................................... 77    0  Mm3
.....................acccuguagGuccgaauuug....................................................................... 1     1  Mm3
.....................Ucccuguagauccgaauuug....................................................................... 1     1  Mm3
.....................acGcuguagauccgaauuugu...................................................................... 7     1  Mm3
.....................acccuguagauccgaauAugu...................................................................... 35    1  Mm3
.....................acccuguagauccgaaAuugu...................................................................... 6     1  Mm3
.....................acccuguaCauccgaauuugu...................................................................... 5     1  Mm3
.....................acAcuguagauccgaauuugu...................................................................... 6     1  Mm3
.....................acccuguagauAcgaauuugu...................................................................... 1     1  Mm3
.....................acccuguagauUcgaauuugu...................................................................... 9     1  Mm3
.....................accAuguagauccgaauuugu...................................................................... 8     1  Mm3
.....................acccuguagauccCaauuugu...................................................................... 1     1  Mm3
.....................acccuguagCuccgaauuugu...................................................................... 10    1  Mm3
.....................acccuguaUauccgaauuugu...................................................................... 10    1  Mm3
.....................acccuguagauccgaauuAgu...................................................................... 5     1  Mm3
.....................aAccuguagauccgaauuugu...................................................................... 19    1  Mm3
.....................acccuAuagauccgaauuugu...................................................................... 8     1  Mm3
.....................Ncccuguagauccgaauuugu...................................................................... 3     1  Mm3
.....................acccuguGgauccgaauuugu...................................................................... 5     1  Mm3
.....................acccuguagauccgaauuuAu...................................................................... 7     1  Mm3
```

```
                    Mature                                  Star
agaguucgcccaucagcagauacccuguagauccgaauuuguuugugaucgauaugacaaauuugaauuugcagcgggaucugaaguuggguauaaucaacugacaggagga
.....................acccuguagauccgaauCugu......................................................................   63      1   Mm3
.....................acccGguagauccgaauuugu......................................................................   4       1   Mm3
.....................acccuguagauccgGauuugu......................................................................   13      1   Mm3
.....................acccuguagauccgaaCuugu......................................................................   5       1   Mm3
.....................acccuguagauccgaCuuugu......................................................................   30      1   Mm3
.....................acccuguCgauccgaauuugu......................................................................   7       1   Mm3
.....................acccuguagauccgaauuugG......................................................................   23      1   Mm3
.....................acccuguaAauccgaauuugu......................................................................   9       1   Mm3
.....................acccuguagaucGgaauuugu......................................................................   3       1   Mm3
.....................acccuguagaAccgaauuugu......................................................................   38      1   Mm3
.....................acccAguagauccgaauuugu......................................................................   4       1   Mm3
.....................acccugCagauccgaauuugu......................................................................   22      1   Mm3
.....................acccuguagauccgaGuuugu......................................................................   9       1   Mm3
.....................acccuguagaGccgaauuugu......................................................................   30      1   Mm3
.....................acccuguagaucAgaauuugu......................................................................   14      1   Mm3
.....................acccuguagauccgaauuugA......................................................................   3       1   Mm3
.....................acccuguUgauccgaauuugu......................................................................   4       1   Mm3
.....................acccuguagaucUgaauuugu......................................................................   12      1   Mm3
.....................acccuguagauccgaUuuugu......................................................................   14      1   Mm3
.....................acccuguagaCccgaauuugu......................................................................   8       1   Mm3
.....................acccCguagauccgaauuugu......................................................................   13      1   Mm3
.....................accUuguagauccgaauuugu......................................................................   6       1   Mm3
.....................acccugAagauccgaauuugu......................................................................   19      1   Mm3
.....................acccuguagauccgaauGugu......................................................................   27      1   Mm3
.....................acccuguagauccgaauuCgu......................................................................   10      1   Mm3
.....................acccuguagauccgaauuugC......................................................................   8       1   Mm3
.....................acccuguagauccUaauuugu......................................................................   3       1   Mm3
.....................acccuguagauccAaauuugu......................................................................   5       1   Mm3
.....................acccuguagauccgaaGuugu......................................................................   4       1   Mm3
.....................acccuguagauGcgaauuugu......................................................................   2       1   Mm3
.....................acccuguagGuccgaauuugu......................................................................   6       1   Mm3
.....................aUccuguagauccgaauuugu......................................................................   1       1   Mm3
.....................acccuguagauccgaauuugu......................................................................   12245   0   Mm3
.....................acccugGagauccgaauuugu......................................................................   4       1   Mm3
.....................acccuguagauccgaauuGgu......................................................................   8       1   Mm3
.....................Ccccuguagauccgaauuugu......................................................................   1       1   Mm3
.....................Ucccuguagauccgaauuugu......................................................................   9       1   Mm3
.....................acccuUuagauccgaauuugu......................................................................   3       1   Mm3
.....................acccuguagauccgCauuugu......................................................................   21      1   Mm3
.....................Gcccuguagauccgaauuugu......................................................................   4       1   Mm3
.....................acccuguagauccgaauuuCu......................................................................   14      1   Mm3
.....................acccuguagUuccgaauuugu......................................................................   2       1   Mm3
.....................acccuguagauccgaauuuUu......................................................................   20      1   Mm3
.....................acUcuguagauccgaauuugu......................................................................   5       1   Mm3
.....................accGuguagauccgaauuugu......................................................................   1       1   Mm3
.....................acccuguagauccgUauuugu......................................................................   8       1   Mm3
.....................acccugGagauccgaauuuguu.....................................................................   1       1   Mm3
.....................acccuguagauccgaauAuguu.....................................................................   1       1   Mm3
.....................acccuguagauccgaauuuguA.....................................................................   55      1   Mm3
.....................acccuguagauccgaauGuguu.....................................................................   2       1   Mm3
.....................acccuguagauccgaauuuUuu.....................................................................   1       1   Mm3
.....................acccuguagauccgaauuuguC.....................................................................   1       1   Mm3
.....................acccuguagauccgaauuuguu.....................................................................   190     0   Mm3
.....................acccuguagauccgaauCuguu.....................................................................   2       1   Mm3
.....................acccuguagaGccgaauuuguu.....................................................................   1       1   Mm3
.....................acccuguaCauccgaauuuguu.....................................................................   2       1   Mm3
.....................aAccuguagauccgaauuuguu.....................................................................   1       1   Mm3
.....................acccuguagauccgaauuuguAu....................................................................   3       1   Mm3
.....................acccuguagauccgaauuuguuA....................................................................   2       1   Mm3
.....................acccuguagauccgaUuuuguuu....................................................................   1       1   Mm3
.....................acccuguagauccgaauuuguuu....................................................................   2       0   Mm3
.....................acccuguagauccgaauuuguuG....................................................................   1       1   Mm3
.....................acccuguagauccgaauuuguuugug.................................................................   1       0   Mm3
......................cccuguagauccgaauuu........................................................................   4       0   Mm3
......................cccuguagauccgaauuug.......................................................................   3       0   Mm3
......................cccugAagauccgaauuug.......................................................................   1       1   Mm3
......................cccuguagauccgaUuuugu......................................................................   4       1   Mm3
......................cccuguagUuccgaauuugu......................................................................   11      1   Mm3
......................cccuguagauccgaauuGgu......................................................................   1       1   Mm3
......................cccuguagauccgaauuugu......................................................................   349     0   Mm3
```

Mature Star

```
agaguucgcccaucagcagauacccuguagauccgaauuuguuugugaucgauaugacaaauuugaauuugcagcgggaucugaaguuggguauaaucaacugacaggagga
......................cccuguagauccgaauuugA......................................................................   1   1   Mm3
......................cccuAuagauccgaauuugu......................................................................   1   1   Mm3
......................cccugAagauccgaauuugu......................................................................   7   1   Mm3
......................cccuguagauccgaauuCgu......................................................................   1   1   Mm3
......................cccuguagaucGgaauuugu......................................................................   1   1   Mm3
......................cccuguaUauccgaauuugu......................................................................   1   1   Mm3
......................cccuguagauccgaauuuUu......................................................................   14  1   Mm3
......................cccuguagauccgaauuAgu......................................................................   3   1   Mm3
......................cccuguagauGcgaauuugu......................................................................   1   1   Mm3
......................cccuguagauccgaauAugu......................................................................   1   1   Mm3
......................cccuguaAauccgaauuugu......................................................................   3   1   Mm3
......................cccuguagauccgaauuugC......................................................................   1   1   Mm3
......................cccuguagUuccgaauuuguu.....................................................................   1   1   Mm3
......................cccuguagauccgaauuuguu.....................................................................   8   0   Mm3
......................cccuguagauccgaauuuguA.....................................................................   1   1   Mm3
.......................ccuguagauccgaauuug.......................................................................   2   0   Mm3
.......................ccuguagauccgaauuAgu......................................................................   2   1   Mm3
.......................ccuguagauccgaauuCgu......................................................................   1   1   Mm3
.......................ccuguagauccgaauuugu......................................................................   50  0   Mm3
.......................ccuguagauccgaauuuguu.....................................................................   1   0   Mm3
........................cuguagauccgaauuugu......................................................................   3   0   Mm3
........................cCguagauccgaauuugu......................................................................   2   1   Mm3
........................cCguagauccgaauuuguu.....................................................................   1   1   Mm3
.........................................................caaauuugaauuugcagcgggau................................   5   0   Mm3
..........................................................aaauuugaauuugcagcgggau................................   4   0   Mm3
..........................................................aaauuugaauuugcagcgggauc...............................   13  0   Mm3
..........................................................aaauuugaauuugcagcgggGuc...............................   1   1   Mm3
..........................................................aaauuugaauuugcagcgggauU...............................   1   1   Mm3
..........................................................aaauuugaauuugcagcgggaucu..............................   3   0   Mm3
...........................................................aauuugaauuugcagcgggauc...............................   2   0   Mm3
```
